# Synthesis and biological activity study of tanshinone I-pyridinium salt derivatives

**DOI:** 10.1007/s13659-025-00534-7

**Published:** 2025-08-08

**Authors:** Huimin Zhao, Yuyang Wang, Zining Liu, Lin Lin, Jiasi Xiang, Zihao Zhu, Xiongli Yang, Yongsheng Fang, Lingmei Kong, Yan Li

**Affiliations:** Key Laboratory of Medicinal Chemistry for Natural Resource, Ministry of Education; Yunnan Key Laboratory of Research and Development for Natural Products; School of Pharmacy, Yunnan University, Kunming, 650500 People’s Republic of China

**Keywords:** Tanshinone I, Pyridinium salts, Structural modification, Antitumor activity, Structure–activity relationship

## Abstract

Natural product tanshinone I exhibits weak potency and poor drug-like properties, which have restricted its clinical development as an anticancer agent. Herein, twenty novel tanshinone I-pyridinium salt derivatives and a pyridinium salt precursor were designed and synthesized, and their antitumor activities were evaluated. Among these tanshinone I-pyridinium salts, compound **a4**, bearing a 4-bromobenzoylmethyl substituent at the *N*-1 position of the pyridine ring, showed the most potent cytotoxicity against breast cancer (MDA-MB-231), hepatocellular carcinoma (HepG2), and prostate cancer (22RV1) cell lines, with IC_50_ values of 1.40–1.63 μM. Preliminary mechanistic studies suggest that **a4** targets PI3Kα with the IC_50_ of 9.24 ± 0.20 μM and exerts effective inhibition of the phosphorylation of key PI3K/Akt/mTOR signaling proteins. Besides, **a4** significantly downregulates the expression of the immune checkpoint protein PD-L1, indicating its potential to activate tumor immunity. These findings demonstrate that tanshinone I-pyridinium salt derivative **a4** is a novel PI3Kα inhibitor, providing a solid foundation for further development of antitumor agents.

**Graphical Abstract:**

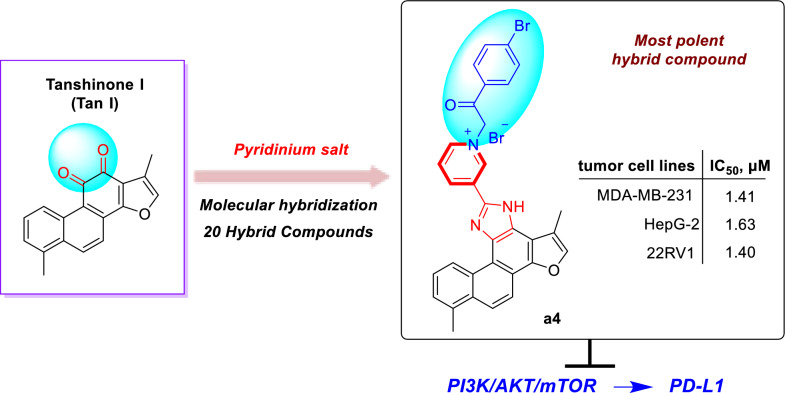

**Supplementary Information:**

The online version contains supplementary material available at 10.1007/s13659-025-00534-7.

## Introduction

Cancer is a complex genomic disorder characterized by significant heterogeneity and drug resistance, greatly complicating its treatment [1]. Although current therapeutic strategies, including chemotherapy, targeted therapy, and immunotherapy, have improved patient survival to some extent, their clinical effectiveness remains limited by poor selectivity, significant toxicity, and acquired drug resistance [2]. Thus, developing novel anticancer agents with high selectivity and low toxicity remains in great demand for current medicinal chemistry research [3, 4].

**Fig. 1:**
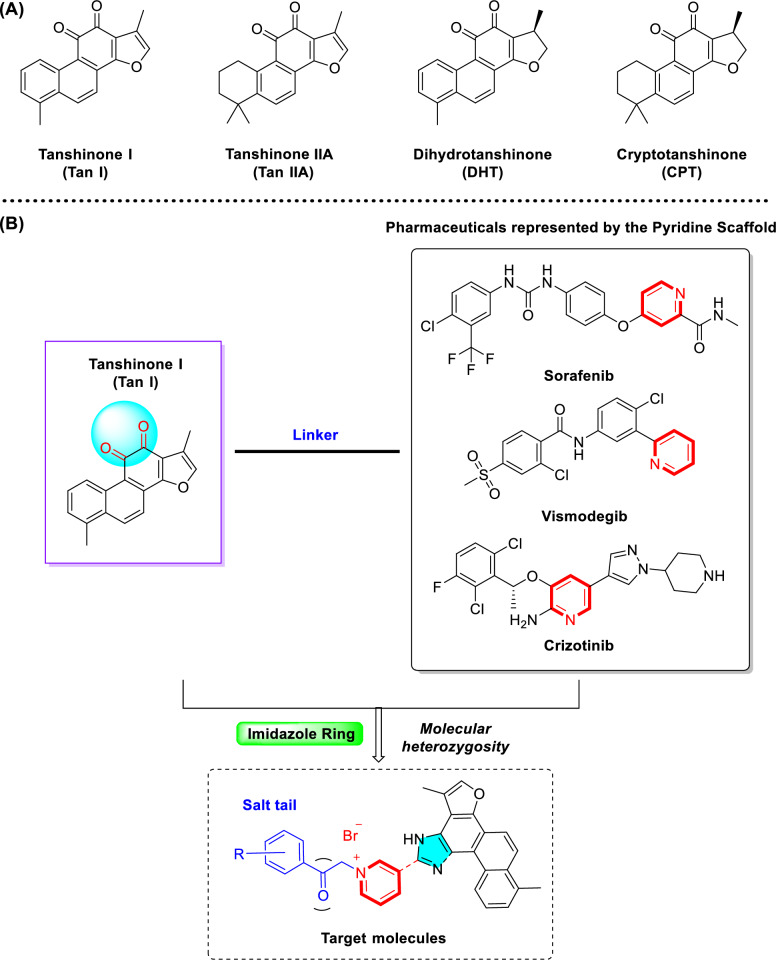
Tanshinone molecular heterozygous design of tanshinone I. **A** Representative tanshinones extracted from *Salvia miltiorrhiza*. **B** Molecular heterozygous design of the natural product tanshinone I with pyridinium salt for drug discovery

Natural products, particularly those derived from traditional Chinese medicines, have long served as valuable sources for anticancer drug discovery due to their structural diversity and multi-targeted effects [5, 6]. *Salvia miltiorrhiza*, a widely-used traditional Chinese medicinal herb for cardiovascular diseases, contains bioactive constituents known as tanshinones that have exhibited potential anticancer activity [7]. Tanshinones, such as tanshinone I (Tan I), tanshinone IIA (Tan IIA), dihydrotanshinone (DHT) and cryptotanshinone (CPT), exhibit multiple anticancer mechanisms, including anti-proliferative effects, apoptosis induction, immune modulation, and angiogenesis inhibition [8–14] (Fig. 1A). Among these, tanshinone I (T1), despite its relatively low abundance and limited research, has demonstrated prominent inhibitory effects against various cancers, including breast, liver, and prostate cancer [15–18]. However, the clinical application of tanshinone I has been restricted by its low aqueous solubility, poor bioavailability, and unfavorable pharmacokinetic properties [19–21].

To enhance its solubility, bioavailability, anticancer activity, and selectivity, structural modification of tanshinone through molecular hybridization by incorporating pharmacophores such as pyridinium salt has been performed in the study [22–25]. Pyridine moieties are widely present in numerous FDA-approved anticancer drugs due to their favorable electronic properties and hydrogen bond acceptor capabilities, which can enhance pharmacokinetic profiles and molecular interactions [26–28]. It has been reported that over 20% of small-molecule anticancer agents contain a pyridine ring or its derivatives. Representative examples include Sorafenib, Vismodegib, and Crizotinib (Fig. 1B). Based on this rationale, we introduced an imidazole linker into ring C of Tan I and synthesized a series of novel tanshinone I-pyridinium salt derivatives, whose anticancer activities and preliminary mechanisms were systematically investigated [29, 30]. This study not only expands structural diversity and biological applications of tanshinone derivatives but also provides new directions for the development of novel anticancer agents.

## Results and discussion

### Chemistry

**Scheme 1:**
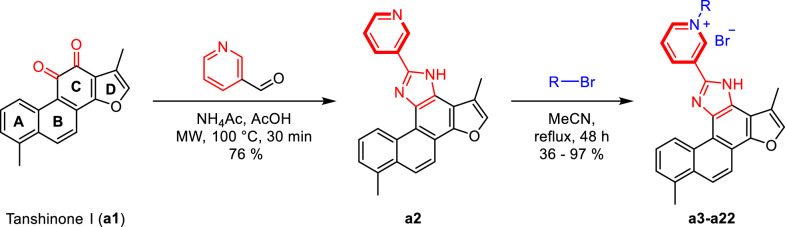
Synthesis of tanshinone I-pyridinium salts Derivatives **a3-a22**

**Fig. 2:**
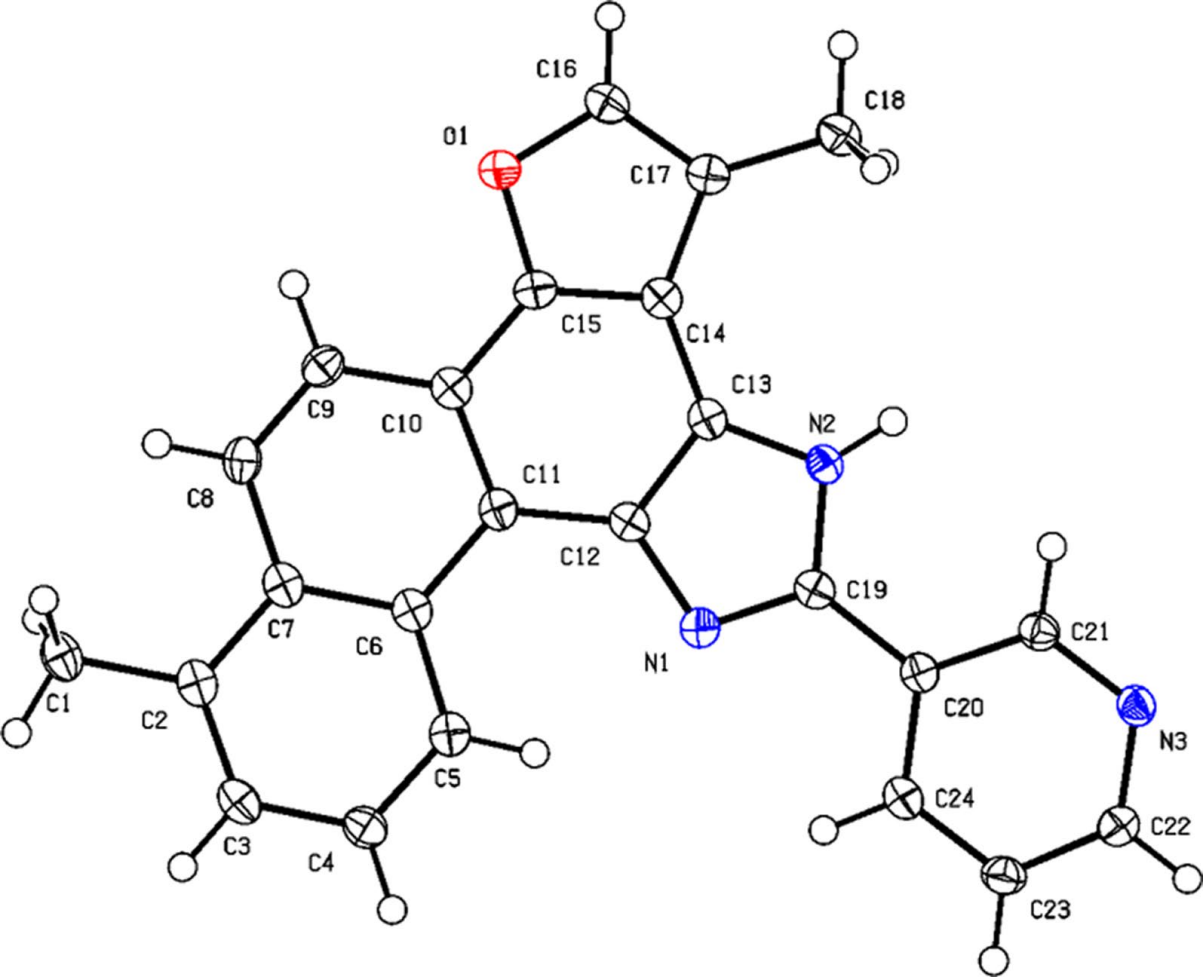
X-ray crystal structure of compound** a2**

As shown in Scheme 1, tanshinone I (**a1**) is structurally characterized by a naphthalene ring A/B, an ortho-quinone ring C, and a furan ring D. To enhance aqueous solubility and molecular stability, structural modification of tanshinone I was performed by introducing an imidazole moiety as a linker onto ring C and a pyridine ring to form the salt, using commercially available tanshinone I (**a1**) as the starting material. Specifically, pyridine derivative **a2** was synthesized via the Debus-Radziszewski reaction of **a1** with 3-pyridinecarboxaldehyde in acetic acid and ammonium acetate under microwave irradiation (100 °C). Single crystals of compound **a2** were obtained by solvent crystallization, and the structure of **a2** was confirmed by single-crystal X-ray diffraction analysis (CCDC 2447458, Fig. 2). Subsequent quaternization of pyridine derivative **a2** with various alkyl bromides in acetonitrile afforded a series of tanshinone I-pyridinium salts (**a3-a22**) in yields ranging from 36 to 97%. To summarize, the structures and yields of all new tanshinone I-pyridinium salts derivatives were shown in Table 1.

**Table 1:** Structures and yields of tanshinone I-pyridinium salts Derivatives **a3-a22**

No	Compounds	R	Molecular Formula	Yields
1	**a3**		C_32_H_24_BrN_3_O_2_	85%
2	**a4**		C_31_H_23_Br_2_N_3_O_2_	76%
3	**a5**		C_33_H_26_BrN_3_O_3_	90%
4	**a6**		C_36_H_26_BrN_3_O_2_	36%
5	**a7**		C_32_H_26_BrN_3_O	67%
6	**a8**		C_32_H_23_BrN_4_O	84%
7	**a9**		C_31_H_26_Br_2_N_3_O	69%
8	**a10**		C_31_H_23_Br_2_N_3_O	89%
9	**a11**		C_31_H_23_BrClN_3_O	92%
10	**a12**		C_31_H_23_BrFN_3_O	97%
11	**a13**		C_32_H_23_BrF_3_N_3_O	85%
12	**a14**		C_32_H_24_BrN_3_O_2_	74%
13	**a15**		C_32_H_26_BrN_3_O	60%
14	**a16**		C_31_H_22_BrCl_2_N_3_O	79%
15	**a17**		C_31_H_22_BrFN_4_O_3_	61%
16	**a18**		C_32_H_25_BrN_4_O_3_	47%
17	**a19**		C_31_H_22_Br_2_N_4_O_3_	40%
18	**a20**		C_35_H_26_BrN_3_O	87%
19	**a21**		C_35_H_26_BrN_3_O	65%
20	**a22**		C_28_H_24_BrN_3_O	73%

### Biological evaluation and structure–activity relationship analysis

#### Biological assay procedures and results

Twenty synthesized tanshinone I-pyridinium salt derivatives (**a3-a22**), together with tanshinone I (**a1**) and pyridine derivative (**a2**), were evaluated in vitro for their cytotoxic activity against three human cancer cell lines, including breast cancer (MDA-MB-231), hepatocellular carcinoma (HepG2), and prostate cancer (22RV1), by using the MTS assay. Cisplatin (DDP) and paclitaxel were chosen as positive controls. The results are summarized in Table 2.

**Table 2:** Cytotoxic activities of tanshinone I-pyridinium salts Derivatives **a3**-**a22** in *vitro*^b^ (IC_50_, µM^a^)


No	Compounds	IC_50_ (μM)		
		**MDA-MB-231**	**HepG2**	**22RV1**
**1**	**a1**	7.17 ± 0.14	6.34 ± 0.02	10.61 ± 2.49
**2**	**a2**	> 20	> 20	> 20
**3**	**a3**	1.06 ± 0.12	1.30 ± 0.06	2.19 ± 0.44
**4**	**a4**	1.41 ± 0.04	1.63 ± 0.04	1.40 ± 0.66
**5**	**a5**	1.19 ± 0.12	1.21 ± 0.01	2.82 ± 0.34
**6**	**a6**	4.13 ± 0.12	6.87 ± 0.85	13.91 ± 2.13
**7**	**a7**	1.35 ± 0.07	2.08 ± 0.01	1.62 ± 0.02
**8**	**a8**	4.10 ± 0.08	8.72 ± 0.14	11.62 ± 3.61
**9**	**a9**	5.56 ± 0.33	7.08 ± 0.20	5.84 ± 1.18
**10**	**a10**	2.32 ± 0.09	7.62 ± 0.24	10.14 ± 0.23
**11**	**a11**	2.01 ± 0.28	5.35 ± 0.32	6.97 ± 1.42
**12**	**a12**	4.08 ± 1.15	7.38 ± 1.38	12.51 ± 3.09
**13**	**a13**	4.21 ± 0.06	7.86 ± 0.52	9.05 ± 2.66
**14**	**a14**	> 20	> 20	> 20
**15**	**a15**	4.68 ± 0.61	5.48 ± 0.75	11.07 ± 3.26
**16**	**a16**	7.11 ± 0.49	9.06 ± 1.42	14.86 ± 1.88
**17**	**a17**	4.95 ± 0.15	7.19 ± 0.32	11.17 ± 1.46
**18**	**a18**	6.44 ± 0.41	9.78 ± 1.04	12.86 ± 0.73
**19**	**a19**	> 20	> 20	> 20
**20**	**a20**	1.70 ± 0.09	2.39 ± 0.15	2.76 ± 1.10
**21**	**a21**	5.41 ± 0.08	5.50 ± 1.01	11.66 ± 0.28
**22**	**a22**	5.66 ± 0.21	8.05 ± 0.29	6.77 ± 1.37
**23**	**DDP**	3.47 ± 0.41	4.60 ± 0.14	5.10 ± 0.14
**24**	**Taxol**	< 0.008	< 0.008	< 0.008

^a^ Cytotoxicity as IC_50_ for each cell line, is the concentration of compound which reduced by 50% the optical density of treated cells with respect to untreated cells using the MTS assay

^b^ Data represent the mean values of three independent determinations

#### Aqueous solubility analysis

The aqueous solubility of selected analogues exhibiting enhanced antitumor activity (**a2**, **a4**, and **a16**) was evaluated by a previously reported ultraviolet–visible (UV–Vis) spectrophotometric method [31]. As anticipated, the conversion of these derivatives into their corresponding pyridinium salts significantly improved their aqueous solubility compared to the parent compound **a1**. Specifically, the pyridinium salts **a4** and **a16** demonstrated notably higher solubilities (85 ± 4 μg/mL and 86 ± 5 μg/mL, respectively) relative to their non-salt precursor **a2** (32 ± 5 μg/mL). These findings highlight the beneficial impact of pyridinium salt formation on enhancing the aqueous solubility of the synthesized compounds. By comparison, the aqueous solubility of compound **a1** was too low to be accurately determined by this method (literature-reported solubility < 0.1 μg/mL [21]).

#### Structure–activity relationship analysis

As presented in Table 2, the majority of tanshinone I-pyridinium salt derivatives showed the potential inhibitory activity. A series of newly synthesized tanshinone I-pyridinium salt derivatives displayed markedly higher in vitro cytotoxic activity than the unmodified parent compound (**a1**), indicating that incorporation of a pyridinium moiety is critical for enhancing the anticancer activity of tanshinone I. Specifically, the nature of the substituent at the *N*-1 position of the pyridine ring profoundly influenced antitumor potency. Pyridinium salts bearing an acyl-linked substituent at *N*-1 (e.g., a benzoylmethyl group) were significantly more potent than those with benzyl or alkyl linkers. Notably, the pyridinium salt derivative with a 4-bromobenzoylmethyl substituent (**a4**) exhibited the best potent cytotoxic activity (IC_50_ = 1.40–1.63 µM). Moreover, the electron-withdrawing 4-bromobenzoylmethyl group (**a4**) conferred greater potency than the electron-donating 4-methoxybenzoylmethyl substituent (**a5**), demonstrating that electron-withdrawing substituent was more favorable for cytotoxic activity. In addition, compounds with a single aromatic-ring substituent generally showed higher activity than those with disubstituted rings, and the substituent’s position had a pronounced effect. For example, the 2-bromobenzyl derivative (**a9**) exhibited higher potency than the 2-bromo-4-nitrobenzyl derivative (**a19**). Furthermore, the meta-substituted analog (**a7**) was significantly more active than the ortho-substituted counterpart (**a15**), this highlights the importance of substituent position. Additionally, naphthalene analogs featuring an acylmethyl linker were more active than those lacking this moiety, further highlighting the key influence of linker structure on cytotoxic potency. Conversely, an aliphatic substituent (isobutenyl in **a22**) yielded much lower activity than aromatic substituents, indicating that such chain substituents provide limited benefit for antitumor potency.

**Scheme 2:**
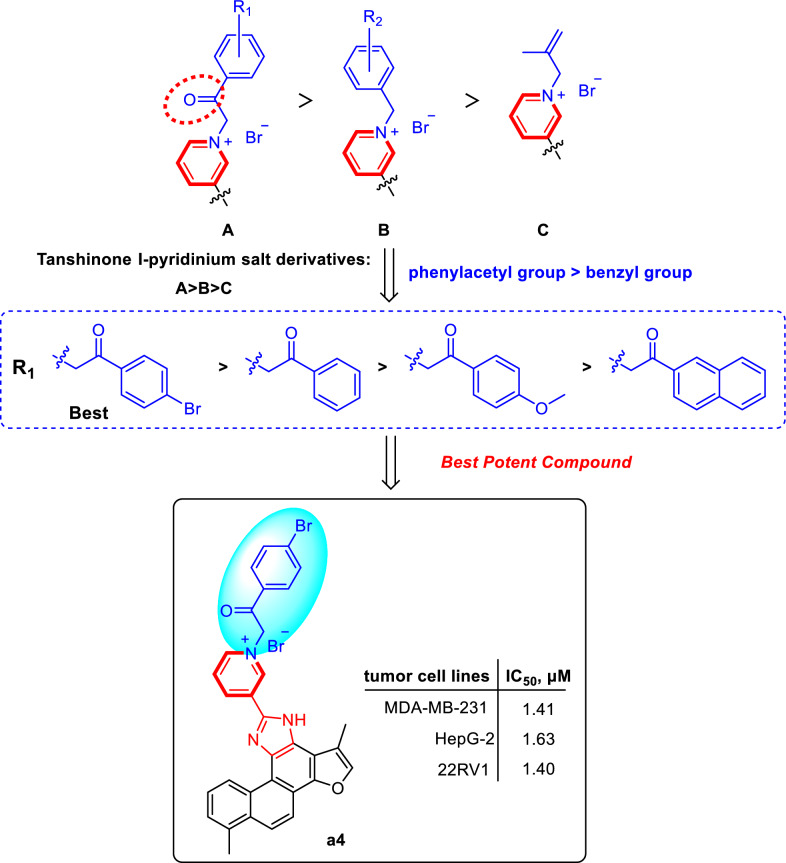
Structure–activity relationships (*SARs*) of tanshinone I Derivatives

In summary, *SAR* analysis demonstrates that introducing a pyridinium fragment is pivotal for boosting the activity of tanshinone I derivatives. The linkage mode of the N-1 substituent, the electronic nature and position of aromatic substituents significantly impact in vitro cytotoxic activity. These results offer an important theoretical basis for further structural optimization. The preliminary structure activity relationships (*SARs*) of the pyridinium salt derivatives were summarized in Scheme 2.

### Compound a4 induces apoptosis and migration

**Fig. 3:**
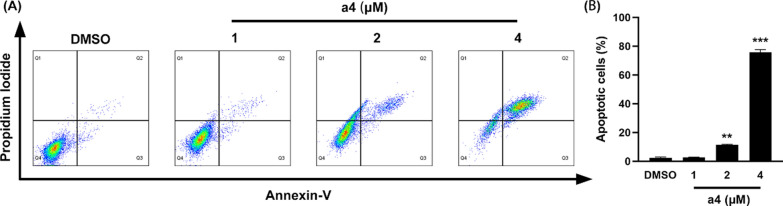
Compound **a4** induced apoptosis of MDA-MB-231 cells. **A** Cells were treated with 1, 2 and 4 μM compound **a4** for 48 h. Cell apoptosis was determined by Annexin V-FITC/Propidium Iodide staining analysis. **B** The quantification of apoptotic cells

To elucidate the potential mechanism underlying the antiproliferative effect of compound **a4**, cell cycle progression and apoptosis were analyzed by flow cytometry. Compound **a4** induced apoptosis by was assessed using Annexin V-FITC/PI double staining assay. As shown in Fig. 3, treatment of MDA-MB-231 cells with compound **a4** at 1, 2, and 4 μM for 48 h significantly increased apoptosis rates to 2.69%, 11.48%, and 75.81%, respectively. These results indicate that compound **a4** induced apoptosis in MDA-MB-231 cells in a dose-dependent manner.

**Fig. 4:**
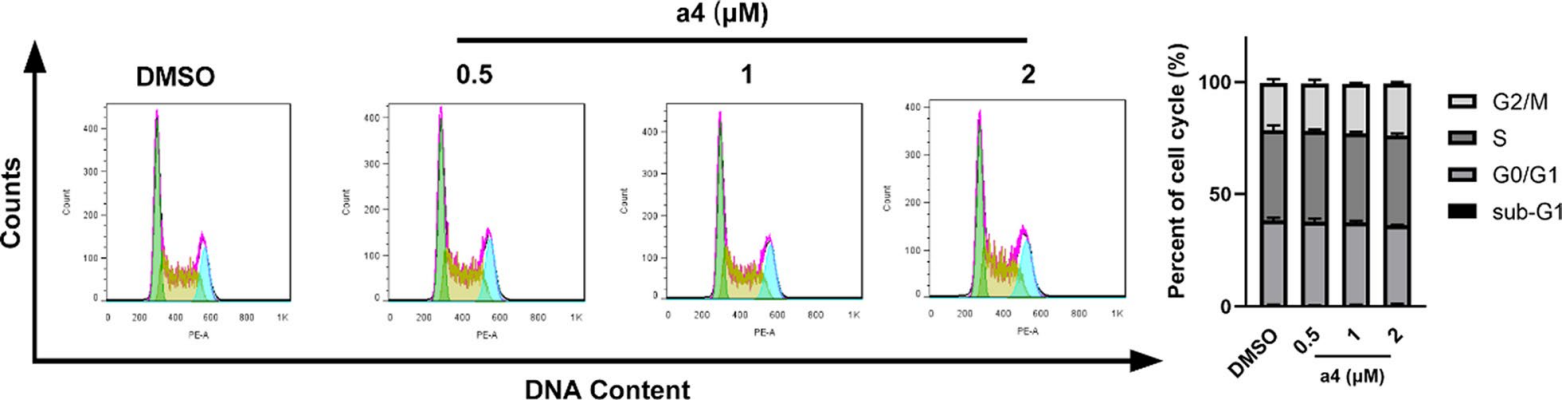
Compound **a4** did not induce significant cell cycle arrest in MDA-MB-231 cells. **A** Cells were treated with different concentrations of compound **a4** (0.5 μM, 1 μM, 2 μM) for 24 h, and cell cycle was determined by cell cytometry with PI staining. **B** The percentages of cells in different phases were quantified

Further, the effect of compound **a4** on the cell cycle distribution of MDA-MB-231 cells was investigated at concentrations of 0.5, 1, and 2 μM. Cell cycle analysis by PI staining and flow cytometry revealed that compound **a4** caused no significant cell cycle arrest in MDA-MB-231 cells (Fig. 4). Collectively, apoptosis induction rather than cell cycle arrest primarily contributed to its anticancer activity of compound **a4**.

**Fig. 5:**
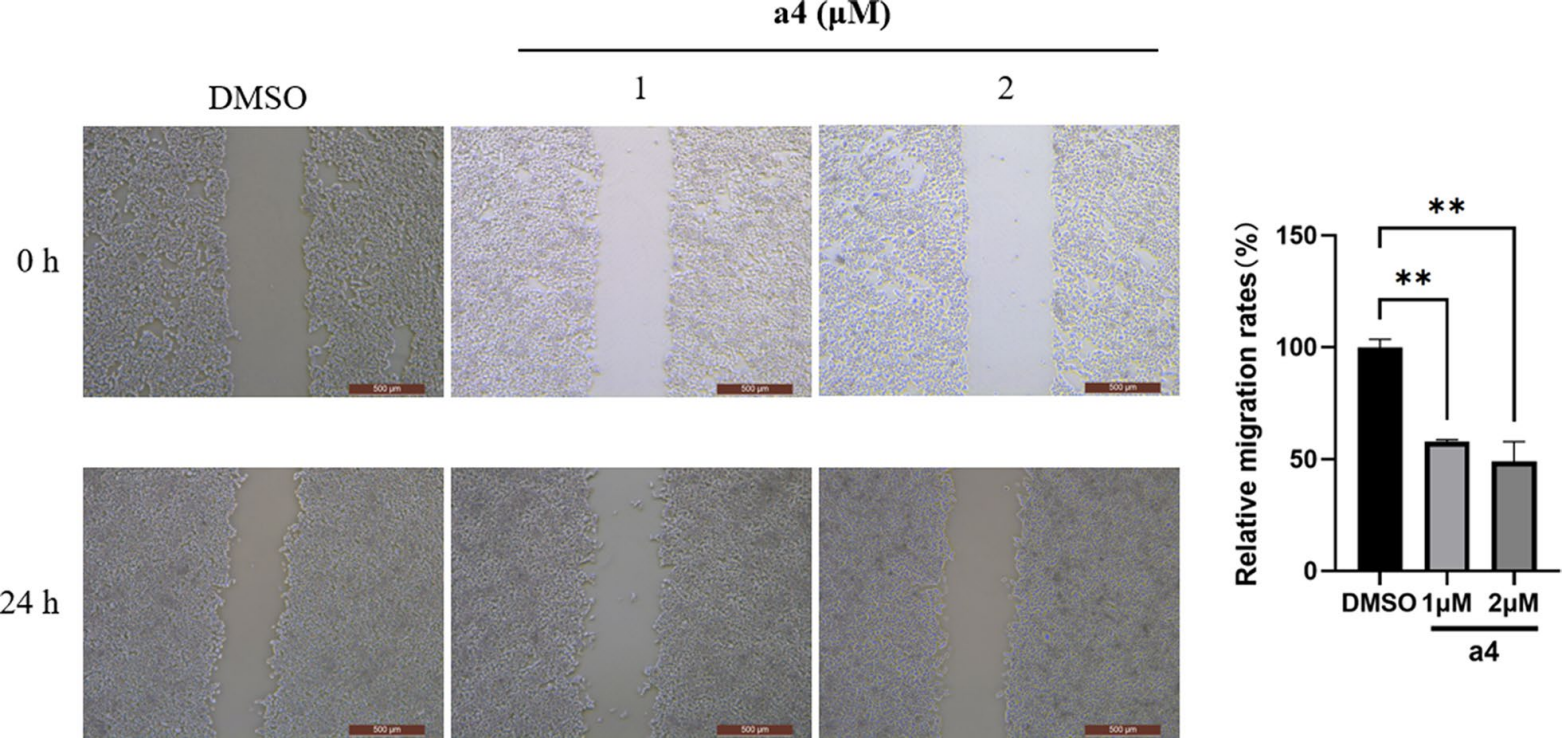
Compound **a4** inhibited migration of MDA-MB-231 cells. MDA-MB-231 cells were exposed to **a4**, and the migration was evaluated with wound healing assay, with the representative images were taken and relative migration rates were calculated respectively. Scale indicates 500 μm

Scratch wound healing assay was conducted to test whether **a4** impaired the migration of MDA-MB-231 cells. The migration rates at 24 h of MDA-MB-231 cells decreased with the increasing **a4** in Fig. 5.

### Compound a4 suppresses PI3K/mTOR and PD-L1

The PI3K/Akt/mTOR signaling ranks one of the most frequently dysregulated signaling pathways in cancer by promoting tumor initiation and progression [32, 33]. Further, abnormal PI3K/AKT/mTOR pathway activation resulted PD-L1 increase facilitates the escape of cancer cells from the immunosurveillance of immune system [34]. Thus, the PI3K/Akt/mTOR signaling components such as PI3K and mTOR are potential therapeutic targets in cancer, and the identification of PI3K/Akt/mTOR signaling inhibitors have drawn extensive attention. In particular, the PI3K has four subtypes (α/β/γ/δ), while overexpression and mutation resulted abnormal activation of PI3Kα is closely to the progression of solid cancers, therefore, PI3Kα has become a key target for the development of anticancer drugs. To date, alpelisib is the only PI3Kα inhibitor approved for the treatment of breast cancer, and several other PI3Kα inhibitors are under evaluation in clinical trials [35], which give us proposal for the development of novel PI3Kα inhibitors for tumor therapy.

**Fig. 6:**
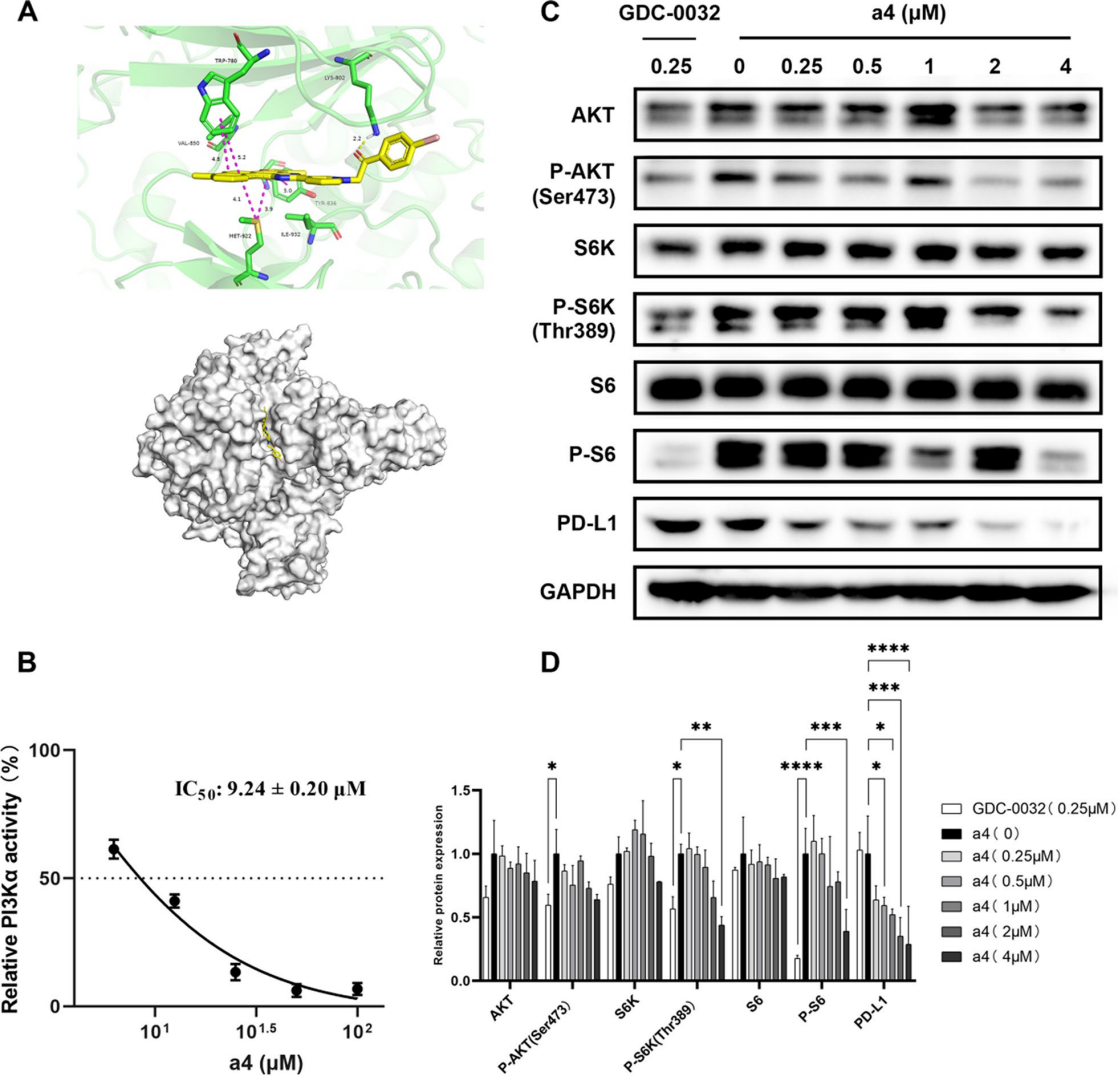
Compound **a4 of** mechanistic studies. (**A**) Molecular docking of PI3Kα protein (PDB ID: 4JPS). (**B**) The compound **a4** inhibits the PI3Kα activity. (**C**) PI3K regulates downstream signaling pathways and PD-L1 protein expression. (**D**) Statistical analysis of the Western blot experimental section, The asterisk (*) represents a statistically significant difference when compared to indicated sample (*p < 0.05, **p < 0.01, ***p < 0.001 and ****p < 0.0001). Error bars represent the standard deviation (SD) of the mean

To further elucidate the underlying mechanism of compound **a4**, molecular docking analysis was performed to identify potential target-proteins and subsequent biological experiments were conducted to confirm the precise target predicted. Molecular docking analysis of compound **a4** with putative targets revealed strong binding affinities toward PI3Kα (Fig. 6A). The compound binds within the PI3Kα active site, forming a hydrogen bond (2.2 Å) between its benzoyl oxygen and the LYS802 residue. The conjugated polycyclic framework of compound **a4** contributes significantly to binding through multiple π-π stacking interactions: two with the benzene ring of TPR780 (4.8 Å and 5.2 Å) and one with TYR836 involving the compound’s furan ring (5.0 Å). Additionally, hydrophobic π-sulfur interactions are observed between the conjugated system of compound **a4** and the sulfur atom of MET922 (4.1 Å and 3.9 Å).

To further confirm the interaction of **a4** and PI3Kα, the PI3Kα inhibitory of **a4** was performed. As shown in Fig. 6B, **a4** inhibited PI3Kα in a dose dependent manner, with an IC_50_ of 9.24 ± 0.20 μM, indicating **a4** was a novel PI3Kα inhibitor. Subsequent western blot analysis demonstrated that compound **a4** exerts dual regulatory effects on the PI3K/Akt/mTOR pathway and tumor immune microenvironment by analyzing key signaling proteins and immune checkpoint molecules (Fig. 6C, D). Using the PI3K inhibitor GDC0032 as a positive control, Western blot results demonstrated that **a4**, while showing no impact on total protein levels of AKT, S6K, or S6, exerted concentration-dependent inhibition on phospho-AKT (Ser473), phospho-S6K (Thr389), and phospho-S6 (Ser235/236), indicating **a4** effectively blocks PI3K/AKT/mTOR signaling. Notably, **a4** exhibited enhanced PD-L1 downregulation. These findings establish a “pathway inhibition-immunoactivation” synergy, offering a novel therapeutic strategy against tumor immune evasion.

## Conclusion

In summary, we have successfully designed and synthesized a series of novel tanshinone I-pyridinium salt derivatives. This method enabled the rapid construction of structurally diverse tanshinone I-pyridinium hybrid molecules, and in vitro activity evaluation demonstrated that these derivatives exhibit potential antitumor activity. Notably, *SARs* revealed that the pyridinium moiety was identified as the critical structural feature responsible for the enhanced in vitro cytotoxic activity of the tanshinone I derivatives. Compound **a4**, featuring a 4-bromobenzoylmethyl substituent at the *N*-1 position of the pyridine ring, was the most active derivative in the series, with IC_50_ values of 1.41, 1.63 and 1.40 μM against human cancer cell lines MDA-MB-231, HepG2 and 22RV1, respectively, indicating that **a4** exhibited the most potent antitumor activity among all derivatives. Meanwhile, compound **a4** demonstrated markedly improved aqueous solubility (85 μg/mL). Furthermore, cell cycle, apoptosis and migration studies indicated that **a4** induces cell cycle arrest, apoptosis and migration inhibition in the MDA-MB-231 breast cancer cell line in a markedly dose-dependent manner. Preliminary mechanistic studies suggest that compound **a4** is a novel PI3Kα inhibitor and exerts its antitumor effects through a dual mechanism. On one hand, it effectively inhibits the phosphorylation of downstream key proteins in the PI3K/Akt/mTOR signaling pathway; on the other hand, it significantly downregulates the expression of the immune checkpoint protein PD-L1. These findings not only provide an efficient structural optimization strategy for the development of tanshinone I-based anticancer agents, but also suggest that compound **a4** is a promising candidate for further antitumor studies.

## Methods and materials

### Chemistry experimental materials

Melting points were obtained and uncorrected on a Haineng melting-point apparatus. Proton nuclear magnetic resonance (^1^H-NMR) spectra were recorded on a Bruker Avance 400 and Bruker Avance 600 spectrometers at 400 MHz and 600 MHz. Carbon-13 nuclear magnetic resonance (^13^C-NMR) was recorded on Bruker Avance 400 spectrometer at 100 MHz. Carbon-13 nuclear magnetic resonance (^13^C-NMR) was recorded on Bruker Avance 600 spectrometer at 150 MHz. Chemical shifts are reported as δ values in parts per million (ppm) relative to tetramethylsilane (TMS) for all recorded NMR spectra. High Resolution Mass spectra were taken on Thermo Fisher LC-MSO/TOR mass spectrometer. Silica gel (200–300 mesh) for column chromatography and silica GF254 for TLC were produced by Qingdao Marine Chemical Company (China). All air- or moisture- sensitive reactions were conducted under an argon atmosphere. Starting materials and reagents used in reactions were obtained commercially from TCI, Adamas, Aladdin, Bidepharm and were used without purification, unless otherwise indicated. Purity of all compounds was determined by high-performance liquid chromatography (HPLC) using an Agilent instrument.

### Synthesis of compounds a2-a22

#### Synthesis of compounds a2

Compound **a1** (0.10 g, 0.36 mmol) was dissolved in 20 mL of analytical-grade acetic acid in a 50 mL microwave tube. Ammonium acetate (0.11 g, 1.45 mmol) was added, followed by the dropwise addition of 3-pyridinecarbaldehyde (0.051 mL, 0.54 mmol) at room temperature. The mixture was stirred for 10 min, then heated to 100 °C in a microwave reactor and maintained for 1 h. After cooling to room temperature, the solution was transferred to a 100 mL round-bottom flask and neutralized to pH 7 using saturated aqueous NaOH. The neutralized mixture was extracted with ethyl acetate (3 × 30 mL), and the aqueous phase was further extracted with ethyl acetate (3 × 30 mL). The combined organic layers were dried over anhydrous Na_2_SO_4_, filtered, and concentrated under reduced pressure. The crude product was purified by column chromatography (silica gel, eluent: dichloromethane/ethyl acetate = 10:1 → 5:1, v/v, containing 1% (v/v) formic acid) to afford **a2** as yellow powder in 76% yield. (Experimental findings & Structural elucidation of **a2** are included in the Supporting information)

*4.2.1.1 1,6-dimethyl-11-(pyridin-3-yl)-12H-furo[2',3':1,2]phenanthro[3,4-d] imidazole (****a2****).* Yield 76%. Yellow powder, m.p. 288–289 °C. ^**1**^**H NMR** (600 MHz, DMSO-*d*_6_) δ 10.74 (d, *J* = 9.0 Hz, 1H), 9.58 (d, *J* = 1.8 Hz, 1H), 8.79 (d, *J* = 7.8 Hz, 1H), 8.62 (dd, *J* = 4.8, 1.2 Hz, 1H), 8.29 (d, *J* = 9.0 Hz, 1H), 8.06 (d, *J* = 9.0 Hz, 1H), 7.96 (d, *J* = 1.8 Hz, 1H), 7.63 (dd, *J* = 8.4, 6.6 Hz, 1H), 7.57 (dd, *J* = 8.4, 4.8 Hz, 1H), 7.45 (d, *J* = 7.2 Hz, 1H), 2.70 (s, 3H), 2.60 (d, *J* = 1.2 Hz, 3H) ppm (one resonance was not observed due to active N–H of imidazole); ^**13**^**C NMR** (150 MHz, DMSO-*d*_6_) δ 150.0, 149.0, 148.2, 146.8, 146.7, 142.1, 136.7, 134.5, 134.0, 130.6, 130.3, 127.2, 126.8, 126.6, 126.5, 123.9, 122.1, 119.2, 119.0, 115.9, 115.6, 112.9, 20.0, 10.0 ppm. **HRMS** (ESI-TOF) m/z Calcd for C_24_H_18_N_3_O [M + H]^+^ 364.1444, found 364.1447. HPLC purity: 98.6%.

#### Synthesis of compounds a3-a22

Compound **a2** (0.05 g, 0.14 mmol) was charged into a 50 mL round-bottom flask and dissolved in ultra-dry acetonitrile (20 mL). The mixture was heated under reflux at 100 °C for 12 h until complete dissolution of **a2** was achieved. Subsequently, various bromides (5.0 equiv relative to **a2**) were introduced sequentially, each dissolved in ultra-dry acetonitrile (20 mL per 50 mg substrate). The reaction mixtures were refluxed with stirring for an additional 24–48 h and monitored by TLC. Upon completion, solvents were removed under reduced pressure, and the precipitated solid was collected by filtration, thoroughly washed with ethyl acetate (3 × 60 mL), and dried under vacuum at 60 °C to afford tanshinone I-pyridinium derivatives **a3-a22** as yellow powder in 36–97% yields. (Structural determination for representative derivative **a4** is detailed in the Supporting information)

*4.2.2.1 3-(1,6-dimethyl-12H-furo[2′,3′:1,2]phenanthro[3,4-d]imidazol-11-yl)-1-(2-oxo-2-phenylethyl) pyridin-1-ium bromide (****a3****).* Yield 85%. Yellow powder, m.p. 287–288 °C. IR *ν*_max_ (cm^−1^): 3429, 3048, 2959, 1703, 1624, 1449, 1369, 1341, 1319, 1218, 1087, 798, 780, 704, 685, 590. ^**1**^**H NMR** (400 MHz, DMSO-*d*_6_) δ 10.67 (d, *J* = 8.8 Hz, 1H), 9.96 (s, 1H), 9.49 (d, *J* = 8.4 Hz, 1H), 9.07 (d, *J* = 6.0 Hz, 1H), 8.49 (dd, *J* = 8.0, 6.0 Hz, 1H), 8.34 (d, *J* = 9.2 Hz, 1H), 8.20—8.13 (m, 3H), 8.04 (d, *J* = 1.2 Hz, 1H), 7.89—7.82 (m, 1H), 7.75 – 7.66 (m, 3H), 7.51 (d, *J* = 6.8 Hz, 1H), 6.71 (s, 2H), 2.76 (s, 3H), 2.61 (s, 3H) ppm (one resonance was not observed due to active N–H of imidazole); ^**13**^**C NMR** (100 MHz, DMSO-*d*_6_) δ 190.8, 149.6, 145.4, 144.4, 142.4, 142.4, 142.3, 136.7, 135.0, 134.1, 133.6 130.6, 130.2, 130.0, 129.2, 128.4, 127.8, 127.3, 126.9, 126.8, 126.7, 122.7, 119.2, 118.8, 116.4, 115.4, 112.5, 66.9, 19.9, 9.8 ppm. **HRMS** (ESI-TOF) m/z Calcd for C_32_H_24_N_3_O_2_ [M]^+^ 482.1863, found 482.1867. HPLC purity: 97.7%.

*4.2.2.2 1-(2-(4-bromophenyl)-2-oxoethyl)-3-(1,6-dimethyl-12H-furo[2',3':1,2]phenanthro[3,4-d]imidazol-11-yl) pyridin-1-ium bromide (****a4****)*. Yield 76%. Yellow powder, m.p. 268–269 °C. IR *ν*_max_ (cm^−1^): 3429,3040, 2951, 1702, 1584, 1396, 1336, 1216, 1098, 998, 821, 727, 705, 688, 591. ^**1**^**H NMR** (600 MHz, DMSO-*d*_6_) δ 13.48 (s, 1H), 10.68 (d, *J* = 8.4 Hz, 1H), 9.96 (s, 1H), 9.52 (d, *J* = 8.4 Hz, 1H), 9.06 (d, *J* = 6.0 Hz, 1H), 8.50 (dd, *J* = 8.4, 6.0 Hz, 1H), 8.36 (d, *J* = 9.0 Hz, 1H), 8.17 (d, *J* = 9.0 Hz, 1H), 8.09 (d, *J* = 8.4 Hz, 2H), 8.06 (d, *J* = 1.2 Hz, 1H), 7.95 (d, *J* = 8.4 Hz, 2H), 7.69 (dd, *J* = 8.4, 7.2 Hz, 1H), 7.52 (d, *J* = 7.2 Hz, 1H), 6.69 (s, 2H), 2.77 (s, 3H), 2.63 (s, 3H) ppm (one resonance was not observed due to active N–H of imidazole); ^**13**^**C NMR** (150 MHz, DMSO-*d*_6_) δ 190.2, 149.6, 145.4, 144.4, 142.5, 142.4, 142.4, 136.7, 134.1, 132.7, 132.4, 130.6, 130.4, 130.3, 130.0, 129.0, 127.8, 127.3, 127.0, 126.7, 126.6, 122.7, 119.2, 118.8, 116.4, 115.4, 112.5, 66.8, 19.9, 9.8 ppm. **HRMS** (ESI-TOF) m/z Calcd for C_32_H_23_O_2_N_3_Br [M]^+^ 560.0968, found 560.0973. HPLC purity: 98.1%.

*4.2.2.3 3-(1,6-dimethyl-12H-furo[2′,3′:1,2]phenanthro[3,4-d]imidazol-11-yl)-1-(2-(4-methoxyphenyl)-2-oxoethyl) pyridin-1-ium bromide (****a5****).* Yield 90%. Yellow powder, m.p. 326–327 °C. IR *ν*_max_ (cm^−1^): 3419, 3048, 2943, 1688, 1623, 1598, 1263, 1174, 1022, 838, 704, 678, 593. ^**1**^**H NMR** (600 MHz, DMSO-*d*_6_) δ 13.53 (s, 1H), 10.71 (d, *J* = 9.0 Hz, 1H), 9.97 (s, 1H), 9.54 (d, *J* = 8.4 Hz, 1H), 9.08 (d, *J* = 6.0 Hz, 1H), 8.50 (dd, *J* = 7.8, 6.0 Hz, 1H), 8.37 (d, *J* = 9.0 Hz, 1H), 8.18 (d, *J* = 9.0 Hz, 1H), 8.13 (d, *J* = 8.4 Hz, 2H), 8.08 (d, *J* = 1.8 Hz, 1H), 7.71—7.67 (m, 1H), 7.53 (d, *J* = 7.2 Hz, 1H), 7.24 (d, *J* = 8.4 Hz, 2H), 6.65 (s, 2H), 3.93 (s, 3H), 2.78 (s, 3H), 2.64 (d, *J* = 1.2 Hz, 3H) ppm (one resonance was not observed due to active N–H of imidazole); ^**13**^**C NMR** (150 MHz, DMSO-*d*_6_) δ 189.0, 164.4, 149.6, 145.5, 144.5, 142.6, 142.5, 142.3, 142.1, 136.7, 134.2, 130.9, 130.6, 130.3, 130.0, 127.8, 127.3, 127.0, 126.8, 126.6, 126.4, 126.4, 122.7, 119.2, 118.8, 116.5, 115.4, 114.5, 112.6, 66.7, 55.9, 19.9, 9.8 ppm. **HRMS** (ESI-TOF) m/z Calcd for C_33_H_26_N_3_O_3_ [M]^+^ 512.1969, found 512.1965. HPLC purity: 96.1%.

*4.2.2.4 3-(1,6-dimethyl-12H-furo[2',3':1,2]phenanthro[3,4-d]imidazol-11-yl)-1-(2-(naphthalen-2-yl)-2-oxoethyl) pyridin-1-ium bromide (****a6****).* Yield 36%. Yellow powder, m.p. 292–293 °C. IR *ν*_max_ (cm^−1^): 3423, 3051, 2922, 1698, 1624, 1467, 1357, 1316, 1278, 1176, 1146, 866, 821, 705, 677. ^**1**^**H NMR** (600 MHz, DMSO-*d*_6_) δ 13.63 (s, 1H), 10.74 (d, *J* = 9.0 Hz, 1H), 10.05 (s, 1H), 9.61 (d, *J* = 7.8 Hz, 1H), 9.14 (d, *J* = 6.0 Hz, 1H), 8.94 (d, *J* = 1.8 Hz, 1H), 8.56 (dd, *J* = 8.4, 6.0 Hz, 1H), 8.40 (d, *J* = 9.0 Hz, 1H), 8.29 (d, *J* = 7.8 Hz, 1H), 8.21 (d, *J* = 9.0 Hz, 2H), 8.14—8.11 (m, 3H), 7.81—7.78 (m, 1H), 7.76—7.73 (m, 1H), 7.68 (dd, *J* = 8.4, 7.2 Hz, 1H), 7.53 (d, *J* = 7.2 Hz, 1H), 6.83 (s, 2H), 2.78 (s, 3H), 2.68 (d, *J* = 1.2 Hz, 3H) ppm (one resonance was not observed due to active N–H of imidazole); ^**13**^**C NMR** (150 MHz, DMSO-*d*_6_) δ 191.1, 150.1, 146.0, 145.1, 143.2, 143.0, 137.3, 136.2, 134.7, 132.5, 131.4, 131.3, 131.1, 130.9, 130.5, 130.3, 130.1, 129.4, 128.5, 128.4, 128.1, 127.8, 127.5, 127.2, 127.0, 123.8, 123.2, 119.7, 119.3, 117.0, 115.9, 113.1, 67.4, 20.3, 10.2 ppm. **HRMS** (ESI-TOF) m/z Calcd for C_36_H_26_N_3_O_2_ [M]^+^ 532.2020, found 532.2020. HPLC purity: 95.9%.

*4.2.2.5 3-(1,6-dimethyl-12H-furo[2′,3′:1,2]phenanthro[3,4-d]imidazol-11-yl)-1-(3-methylbenzyl) pyridin-1-ium bromide (****a7****).* Yield 67%. Yellow powder, m.p. 280–281 °C. IR *ν*_max_ (cm^−1^): 3377, 3006, 1590, 1397, 1302, 1161, 819, 712, 593. ^**1**^**H NMR** (600 MHz, DMSO-*d*_6_) δ 10.57 (s, 1H), 10.06 (s, 1H), 9.30 (s, 1H), 9.21 (s, 1H), 8.34 (d, *J* = 7.2 Hz, 1H), 8.27 (d, *J* = 9.0 Hz, 1H), 8.08 (d, *J* = 9.0 Hz, 1H), 7.96 (s, 1H), 7.67 (t, *J* = 7.8 Hz, 1H), 7.56 (s, 1H), 7.53 (d, *J* = 7.2 Hz, 1H), 7.49 (d, *J* = 6.6 Hz, 1H), 7.45 (t, *J* = 7.2 Hz, 1H), 7.33 (d, *J* = 6.0 Hz, 1H), 6.05 (s, 2H), 2.74 (s, 3H), 2.57 (s, 3H), 2.38 (s, 3H) ppm (one resonance was not observed due to active N–H of imidazole); ^**13**^**C NMR** (150 MHz, DMSO) δ 149.8, 144.0, 142.9, 142.6, 142.1, 139.2, 136.9, 134.4, 134.4, 131.2, 130.9, 130.6, 130.3, 130.1, 129.7, 128.7, 127.6, 127.0, 126.9, 126.7, 122.8, 119.4, 119.2, 116.7, 115.8, 113.0, 64.0, 21.4, 20.3, 10.3. ppm; **HRMS** (ESI-TOF) m/z Calcd for C_32_H_26_N_3_O [M]^+^ 468.2070, found 468.2074. HPLC purity: 97.6%.

*4.2.2.6 1-(2-cyanobenzyl)-3-(1,6-dimethyl-12H-furo[2′,3′:1,2]phenanthro[3,4-d]imidazol-11-yl) pyridin-1-ium bromide (****a8****).* Yield 84%. Yellow powder, m.p. 285–286 °C. IR *ν*_max_ (cm^−1^): 3426, 3069, 3028, 2961, 2919, 2854, 2223, 1878, 1624, 1489, 1456, 1314, 1146, 816, 773, 702, 686. ^**1**^**H NMR** (600 MHz, DMSO-*d*_6_) δ 13.54 (s, 1H), 10.68 (d, *J* = 8.4 Hz, 1H), 10.06 (d, *J* = 1.8 Hz, 1H), 9.51 (d, *J* = 7.8 Hz, 1H), 9.22 (d, *J* = 6.0 Hz, 1H), 8.47 (dd, *J* = 7.8, 6.0 Hz, 1H), 8.36 (d, *J* = 9.0 Hz, 1H), 8.18 (d, *J* = 9.0 Hz, 1H), 8.11 (dd, *J* = 7.8, 1.2 Hz, 1H), 8.07 (d, *J* = 1.8 Hz, 1H), 7.88 (td, *J* = 7.8, 1.2 Hz, 1H), 7.74—7.67 (m, 3H), 7.55 (d, *J* = 7.2 Hz, 1H), 6.37 (s, 2H), 2.78 (s, 3H), 2.63 (d, *J* = 1.2 Hz, 3H) ppm (one resonance was not observed due to active N–H of imidazole); ^**13**^**C NMR** (150 MHz, DMSO-*d*_6_) δ 149.6, 144.2, 143.6, 142.5, 142.4, 142.2, 137.0, 136.7, 134.3, 134.1, 134.0, 130.9, 130.6, 130.2, 130.0, 129.8, 128.7, 127.3, 127.0, 126.7, 126.6, 122.8, 119.1, 118.8, 117.1, 116.5, 115.4, 112.6, 111.5, 61.9, 19.9, 9.8 ppm. **HRMS** (ESI-TOF) m/z Calcd for C_32_H_23_N_4_O [M]^+^ 479.1866, found 479.1870. HPLC purity: 97.9%.

*4.2.2.7 1-(2-bromobenzyl)-3-(1,6-dimethyl-12H-furo[2′,3′:1,2]phenanthro[3,4-d]imidazol-11-yl) pyridin-1-ium bromide (****a9****).* Yield 69%. Yellow powder, m.p. 260–261 °C. IR *ν*_max_ (cm^−1^): 3423, 3083, 3006, 2933, 2860, 1805, 1627, 1491, 1473, 1371, 1165, 1048, 1027, 812, 757, 679. ^**1**^**H NMR** (600 MHz, DMSO-*d*_6_) δ 13.55 (s, 1H), 10.62 (d, *J* = 8.4 Hz, 1H), 9.98 (s, 1H), 9.51 (d, *J* = 8.4 Hz, 1H), 9.19 (d, *J* = 6.0 Hz, 1H), 8.46 (t, *J* = 7.2 Hz, 1H), 8.37 (d, *J* = 9.0 Hz, 1H), 8.19 (d, *J* = 9.0 Hz, 1H), 8.08 (s, 1H), 7.89 (d, *J* = 7.8 Hz, 1H), 7.70 (t, *J* = 7.8 Hz, 1H), 7.63—7.55 (m, 3H), 7.51 (d, *J* = 7.2 Hz, 1H), 6.19 (s, 2H), 2.79 (s, 3H), 2.64 (s, 3H) ppm (one resonance was not observed due to active N–H of imidazole); ^**13**^**C NMR** (150 MHz, DMSO-*d*_6_) δ 149.6, 144.2, 143.1, 142.5, 142.4, 142.1, 136.7, 134.2, 133.6, 133.1, 131.8, 131.6, 130.7, 130.6, 129.9, 128.9, 128.6, 127.4, 127.0, 126.7, 126.5, 123.6, 122.8, 119.1, 118.8, 116.5, 115.4, 112.6, 63.7, 19.9, 9.7 ppm. **HRMS** (ESI-TOF) m/z Calcd for C_31_H_23_N_3_OBr [M]^+^ 532.1019, found 532.1024. HPLC purity: 98.9%.

*4.2.2.8 1-(4-bromobenzyl)-3-(1,6-dimethyl-12H-furo[2′,3′:1,2]phenanthro[3,4-d]imidazol-11-yl) pyridin-1-ium bromide (****a10****).* Yield 89%. Yellow powder, m.p. 285–286 °C. IR *ν*_max_ (cm^−1^): 3382, 3091, 3037, 2993, 2927, 1904, 1584, 1484, 1442, 1351, 1169, 1071, 838, 814, 649. ^**1**^**H NMR** (400 MHz, DMSO-*d*_6_) δ 10.57 (d, *J* = 8.4 Hz, 1H), 10.07 (s, 1H), 9.30 (d, *J* = 8.0 Hz, 1H), 9.21 (d, *J* = 6.0 Hz, 1H), 8.35 (dd, *J* = 8.4, 6.0 Hz, 1H), 8.27 (d, *J* = 9.2 Hz, 1H), 8.09 (d, *J* = 9.2 Hz, 1H), 8.00—7.94 (m, 1H), 7.78 (d, *J* = 8.4 Hz, 2H), 7.72 (d, *J* = 8.4 Hz, 2H), 7.68 (dd, *J* = 8.8, 7.2 Hz, 1H), 7.49 (d, *J* = 6.8 Hz, 1H), 6.09 (s, 2H), 2.74 (s, 3H), 2.56 (s, 3H) ppm (one resonance was not observed due to active N–H of imidazole); ^**13**^**C NMR** (100 MHz, DMSO-*d*_6_) δ 149.5, 143.7, 142.7, 142.2, 141.7, 136.5, 134.0, 133.3, 132.3, 131.6, 130.6, 130.5, 129.9, 128.4, 127.2, 126.7, 126.6, 123.2, 122.5, 119.0, 118.7, 116.3, 115.3, 112.4, 62.8, 19.9, 9.9 ppm. **HRMS** (ESI-TOF) m/z Calcd for C_31_H_23_N_3_OBr [M]^+^ 532.1019, found 532.1022. HPLC purity: 98.0%.

*4.2.2.9 1-(4-chlorobenzyl)-3-(1,6-dimethyl-12H-furo[2′,3′:1,2]phenanthro[3,4-d]imidazol-11-yl) pyridin-1-ium bromide (****a11****).* Yield 92%. Yellow powder, m.p. 261–262 °C. IR *ν*_max_ (cm^−1^): 3422, 3092, 3038, 2923, 2853, 1629, 1594, 1489, 1399, 1283, 1143, 1088, 842, 776, 700, 677. ^**1**^**H NMR** (400 MHz, DMSO-*d*_6_) δ 10.59 (d, *J* = 8.4 Hz, 1H), 10.08 (s, 1H), 9.32 (d, *J* = 8.4 Hz, 1H), 9.21 (d, *J* = 6.0 Hz, 1H), 8.36 (dd, *J* = 8.0, 2.0 Hz, 1H), 8.28 (d, *J* = 9.2 Hz, 1H), 8.10 (d, *J* = 9.2 Hz, 1H), 7.99 (d, *J* = 1.6 Hz, 1H), 7.83—7.76 (m, 2H), 7.71—7.66 (m, 1H), 7.66—7.62 (m, 2H), 7.50 (d, *J* = 6.8 Hz, 1H), 6.11 (s, 2H), 2.75 (s, 3H), 2.58 (s, 3H) ppm (one resonance was not observed due to active N–H of imidazole); ^**13**^**C NMR** (101 MHz, DMSO-*d*_6_) δ 149.5, 143.7, 142.7, 142.3, 141.8, 136.5, 134. 5, 134.0, 132.9, 131.4, 130.6, 130.5, 129.9, 129.4, 128.4, 127.2, 126.8, 126.6, 122.6, 119.0, 118.7, 116.4, 115.3, 112.5, 62.7, 19.9, 9.9 ppm. **HRMS** (ESI-TOF) m/z Calcd for C_31_H_23_ON_3_Cl [M]^+^ 488.1524, found 488.1526. HPLC purity: 96.2%.

*4.2.2.10 3-(1,6-dimethyl-12H-furo[2′,3′:1,2]phenanthro[3,4-d]imidazol-11-yl)-1-(4-fluorobenzyl) pyridin-1-ium bromide (****a12****).* Yield 97%. Yellow powder, m.p. 276–277 °C. IR *ν*_max_ (cm^−1^): 3426, 3009, 2941, 1627, 1591, 1526, 1318, 1228, 1160, 1144, 818, 773, 707, 684. ^**1**^**H NMR** (400 MHz, DMSO-*d*_6_) δ 10.63 (d, *J* = 8.4 Hz, 1H), 10.08 (s, 1H), 9.38 (d, *J* = 8.4 Hz, 1H), 9.22 (d, *J* = 5.6 Hz, 1H), 8.38 (dd, *J* = 8.4, 6.0 Hz, 1H), 8.33 (d, *J* = 9.2 Hz, 1H), 8.15 (d, *J* = 9.2 Hz, 1H), 8.04 (d, *J* = 1.6 Hz, 1H), 7.87—7.81 (m, 2H), 7.71 (dd, *J* = 8.8, 6.8 Hz, 1H), 7.54 (d, *J* = 6.8 Hz, 1H), 7.45—7.39 (m, 2H), 6.09 (s, 2H), 2.77 (s, 3H), 2.63—2.60 (m, 3H) ppm (one resonance was not observed due to active N–H of imidazole); ^**13**^**C NMR** (100 MHz, DMSO-*d*_6_) δ 162.79 (d, ^1^*J*_C-F_ = 244.9 Hz), 149.6, 143.7, 142.7, 142.44, 142.38, 141.8, 136.6, 134.1, 132.0 (d, ^3^*J*_C-F_ = 8.5 Hz), 130.6, 130.60, 130.2 (d, ^4^*J*_C-F_ = 3.1 Hz), 130.0, 128.4, 127.3, 126.8, 126.6, 122.7, 119.1, 118.8, 116.3 (d, ^2^*J*_C-F_ = 21.8 Hz), 115.4, 112.5, 62.8, 19.9, 9.9 ppm. **HRMS** (ESI-TOF) m/z Calcd for C_31_H_23_ON_3_F [M]^+^ 472.1820, found 472.1818. HPLC purity: 98.6%.

*4.2.2.11 3-(1,6-dimethyl-12H-furo[2′,3′:1,2]phenanthro[3,4-d]imidazol-11-yl)-1-(4-(trifluoromethyl) benzyl)pyridin-1-ium bromide (****a13****).* Yield 85%. Yellow powder, m.p. 273–274 ℃. ^**1**^**H NMR** (600 MHz, DMSO-*d*_6_) δ 13.55 (s, 1H), 10.70 (d, *J* = 8.4 Hz, 1H), 10.16 (s, 1H), 9.48 (d, *J* = 8.4 Hz, 1H), 9.23 (d, *J* = 6.0 Hz, 1H), 8.43 (dd, *J* = 8.4, 6.0 Hz, 1H), 8.37 (d, *J* = 9.0 Hz, 1H), 8.19 (d, *J* = 9.0 Hz, 1H), 8.08 (d, *J* = 1.8 Hz, 1H), 7.95—7.88 (m, 4H), 7.72 (dd, *J* = 8.4, 6.6 Hz, 1H), 7.56 (d, *J* = 6.6 Hz, 1H), 6.22 (s, 2H), 2.79 (s, 3H), 2.65 (d, *J* = 1.2 Hz, 3H) ppm (one resonance was not observed due to active N–H of imidazole); ^**13**^**C NMR** (150 MHz, DMSO-*d*_6_) δ 149.6, 144.1, 143.3, 142.6, 142.5, 142.2, 138.6, 136.7, 134.2, 130.6 (q, ^2^*J*_C-F_ = 35.9 Hz), 129.7, 130.0, 129.9, 128.6, 127.4, 126.9, 126.7 (q, ^3^*J*_C-F_ = 9.15 Hz), 126.2 (q, ^4^*J*_C-F_ = 3.9 Hz), 124.0 (q, ^1^*J*_C-F_ = 270.8 Hz), 122.7, 119.2, 118.8, 116.5, 115.4, 112.6, 62.9, 19.9, 9.8 ppm; **HRMS** (ESI-TOF) m/z Calcd for C_32_H_23_N_3_OF_3_ [M]^+^ 522.1788, found 522.1788. HPLC purity: 97.0%.

*4.2.2.12 3-(1,6-dimethyl-12H-furo[2′,3′:1,2]phenanthro[3,4-d]imidazol-11-yl)-1-(4-formylbenzyl) pyridin-1-ium bromide (****a14****).* Yield 74%. Yellow powder, m.p. 243–244 °C. IR *ν*_max_ (cm^−1^): 3424, 3076, 2926, 2851, 1696, 1624, 1455, 1211, 1170, 814, 778, 702, 682, 594. ^**1**^**H NMR** (600 MHz, DMSO-*d*_6_) δ 13.52 (s, 1H), 10.68 (d, *J* = 9.0 Hz, 1H), 10.15 (s, 1H), 10.08 (s, 1H), 9.47 (d, *J* = 8.4 Hz, 1H), 9.24 (d, *J* = 6.0 Hz, 1H), 8.43 (dd, *J* = 8.4, 6.0 Hz, 1H), 8.36 (d, *J* = 9.0 Hz, 1H), 8.18 (d, *J* = 9.0 Hz, 1H), 8.09—8.03 (m, 3H), 7.88 (d, *J* = 7.8 Hz, 2H), 7.71 (t, *J* = 7.8 Hz, 1H), 7.55 (d, *J* = 7.2 Hz, 1H), 6.23 (s, 2H), 2.78 (s, 3H), 2.68—2.59 (m, 3H) ppm (one resonance was not observed due to active N–H of imidazole); ^**13**^**C NMR** (150 MHz, DMSO-*d*_6_) δ 192.9, 149.6, 144.1, 143.3, 142.5, 142.4, 142.2, 140.2, 136.7, 136.6, 134.2, 130.9, 130.6, 130.3, 130.0, 129.6, 128.6, 127.3, 126.9, 126.7, 126.6, 122.7, 119.6, 118.8, 116.5, 115.4, 112.6, 63.2, 19.9, 9.8 ppm. **HRMS** (ESI-TOF) m/z Calcd for C_32_H_24_N_3_O_2_ [M]^+^ 482.1863, found 482.1866.

*4.2.2.13 3-(1,6-dimethyl-12H-furo[2′,3′:1,2]phenanthro[3,4-d]imidazol-11-yl)-1-(4-methylbenzyl) pyridin-1-ium bromide (****a15****).* Yield 60%. Yellow powder, m.p. 250–251 °C. IR *ν*_max_ (cm^−1^): 3421, 3011, 2921, 1628, 1589, 1443, 1303, 1143, 1068, 816, 774, 678, 609, 595. ^**1**^**H NMR** (600 MHz, DMSO-*d*_6_) δ 13.59 (s, 1H), 10.70 (d, *J* = 8.4 Hz, 1H), 10.07 (s, 1H), 9.46 (d, *J* = 8.4, 1H), 9.19 (d, *J* = 6.0 Hz, 1H), 8.43—8.37 (m, 2H), 8.21 (d, *J* = 9.0 Hz, 1H), 8.10 (d, *J* = 1.8 Hz, 1H), 7.73 (dd, *J* = 8.4, 6.6 Hz, 1H), 7.61—7.57 (m, 3H), 7.36 (d, *J* = 7.8 Hz, 2H), 6.04 (s, 2H), 2.80 (s, 3H), 2.66 (d, *J* = 1.2 Hz, 3H), 2.35 (s, 3H) ppm (one resonance was not observed due to active N–H of imidazole); ^**13**^**C NMR** (150 MHz, DMSO-*d*_6_) δ 149.6, 143.8, 142.8, 142.7, 142.5, 142.0, 139.2, 136.7, 134.2, 131.0, 130.7, 130.7, 130.0, 129.9, 129.3, 129.2, 128.5, 128.4, 127.4, 127.0, 126. 7, 126.6, 122.8, 119.2, 118.8, 116.5, 115.4, 112.6, 63.6, 20.8, 19.9, 9.7 ppm. **HRMS** (ESI-TOF) m/z Calcd for C_32_H_26_N_3_O [M]^+^ 468.2070, found 468.2071. HPLC purity: 95.6%.

*4.2.2.14 1-(3,4-dichlorobenzyl)-3-(1,6-dimethyl-12H-furo[2′,3′:1,2]phenanthro[3,4-d]imidazol-11-yl)pyridin-1-ium bromide (****a16****).* Yield 79%. Yellow powder, m.p. 297–298 °C. IR *ν*_max_ (cm^−1^): 3427, 3007, 1626, 1587, 1491, 1468, 1397, 1146, 817, 773, 707, 687. ^**1**^**H NMR** (400 MHz, DMSO-*d*_6_) δ 10.62 (d, *J* = 8.4 Hz, 1H), 10.11 (s, 1H), 9.32 (d, *J* = 8.0 Hz, 1H), 9.22 (d, *J* = 6.0 Hz, 1H), 8.36 (dd, *J* = 8.0, 6.0 Hz, 1H), 8.28 (d, *J* = 9.2 Hz, 1H), 8.16 (d, *J* = 2.0 Hz, 1H), 8.11 (d, *J* = 9.2 Hz, 1H), 7.99 (d, *J* = 1.6 Hz, 1H), 7.85 (d, *J* = 8.4 Hz, 1H), 7.78 (dd, *J* = 8.4, 2.0 Hz, 1H), 7.72—7.67 (m, 1H), 7.51 (d, *J* = 6.8 Hz, 1H), 6.11 (s, 2H), 2.75 (s, 3H), 2.58 (s, 3H) ppm (one resonance was not observed due to active N–H of imidazole); ^**13**^**C NMR** (100 MHz, DMSO-*d*_6_) δ 149.5, 143.7, 143.0, 142.3, 142.3, 141.9, 136.5, 134.7, 134.0, 132.5, 131.9, 131.7, 131.5, 130.7, 130.5, 129.9, 129.9, 128.4, 127.2, 126.7, 126.7, 126.6, 122.6, 119.1, 118.7, 116.4, 115.3, 112.4, 62.1, 19.9, 9.9 ppm. **HRMS** (ESI-TOF) m/z Calcd for C_31_H_22_N_3_OCl_2_ [M]^+^ 522.1134, found 522.1139. HPLC purity: 98.7%.

*4.2.2.15 3-(1,6-dimethyl-12H-furo[2′,3′:1,2]phenanthro[3,4-d]imidazol-11-yl)-1-(3-fluoro-4-nitrobenzyl) pyridin-1-ium bromide (****a17****).* Yield 61%. Yellow powder, m.p. 241–242 °C. IR *ν*_max_ (cm^−1^): 3417, 3015, 2922, 1603, 1528, 1348, 1277, 1090, 841, 818, 746, 711, 677, 538. ^**1**^**H NMR** (600 MHz, DMSO-*d*_6_) δ 13.62 (s, 1H), 10.76 (d, *J* = 8.4 Hz, 1H), 10.18 (s, 1H), 9.53 (dt, *J* = 7.8, 1.8 Hz, 1H), 9.21 (d, *J* = 6.0 Hz, 1H), 8.46 (dd, *J* = 7.8, 6.0 Hz, 1H), 8.40 (d, *J* = 9.0 Hz, 1H), 8.32 (t, *J* = 8.4 Hz, 1H), 8.22 (d, *J* = 9.0 Hz, 1H), 8.11 (d, *J* = 1.2 Hz, 1H), 7.98 (dd, *J* = 12.0, 1.8 Hz, 1H), 7.76—7.71 (m, 2H), 7.58 (d, *J* = 7.2 Hz, 1H), 6.23 (s, 2H), 2.80 (s, 3H), 2.68 (d, *J* = 1.2 Hz, 3H) ppm (one resonance was not observed due to active N–H of imidazole); ^**13**^**C NMR** (150 MHz, DMSO-*d*_6_) δ 154.6 (d, ^1^*J*_C-F_ = 260.9 Hz), 149.6, 144.2, 143.8, 142.7, 142.6, 142.5 (d, ^3^*J*_C-F_ = 8.1 Hz), 137.2, 137.2, 136.8, 134.3, 131.9 (d, ^2^*J*_C-F_ = 54.7 Hz), 130.0, 128.7, 127.4, 127.1, 127.0, 126.7 (d, ^3^*J*_C-F_ = 9.0 Hz), 125.7 (d, ^4^*J*_C-F_ = 3.9 Hz), 122.8, 119.2, 119.1, 118.9 (d, ^2^*J*_C-F_ = 24.9 Hz), 116.5, 115.4, 112.6, 62.2, 19.9, 9.8 ppm. **HRMS** (ESI-TOF) m/z Calcd for C_31_H_22_N_4_O_3_F [M]^+^ 517.1670, found 517.1670. HPLC purity: 96.3%.

*4.2.2.16 3-(1,6-dimethyl-12H-furo[2′,3′:1,2]phenanthro[3,4-d]imidazol-11-yl)-1-(3-methyl-4-nitrobenzyl) pyridin-1-ium bromide (****a18****).* Yield 47%. Yellow powder, m.p. 275–276 °C. IR *ν*_max_ (cm^−1^): 3423, 3014, 2926, 1615, 1522, 1345, 1281, 1144, 967, 842, 820, 775, 679, 595. ^**1**^**H NMR** (600 MHz, DMSO-*d*_6_) δ 13.60 (s, 1H), 10.74 (d, *J* = 8.4 Hz, 1H), 10.16 (s, 1H), 9.51 (d, *J* = 8.4 Hz, 1H), 9.21 (d, *J* = 6.0 Hz, 1H), 8.44 (dd, *J* = 7.8, 6.0 Hz, 1H), 8.39 (d, *J* = 9.6 Hz, 1H), 8.21 (d, *J* = 9.0 Hz, 1H), 8.12 (d, *J* = 8.4 Hz, 1H), 8.10 (d, *J* = 1.2 Hz, 1H), 7.81 (d, *J* = 1.8 Hz, 1H), 7.76—7.71 (m, 2H), 7.57 (d, *J* = 6.6 Hz, 1H), 6.18 (s, 2H), 2.80 (s, 3H), 2.67 (d, *J* = 1.2 Hz, 3H), 2.55 (s, 3H) ppm (one resonance was not observed due to active N–H of imidazole); ^**13**^**C NMR** (150 MHz, DMSO-*d*_6_) δ 149.6, 149.3, 144.1, 143.5, 142.6, 142.5, 142.3, 139.1, 136.6, 134.2, 133.5, 133.1, 131.0, 130.7, 130.0, 128.7, 127.8, 127.4, 127.0, 126.7, 126.6, 125.2, 122.8, 119.2, 118.8, 116.5, 115.4, 112.6, 62.7, 19.9, 19.4, 9.8 ppm. **HRMS** (ESI-TOF) m/z Calcd for C_32_H_25_N_4_O_3_ [M]^+^ 513.1921, found 513.1917. HPLC purity: 96.9%.

*4.2.2.17 1-(2-bromo-4-nitrobenzyl)-3-(1,6-dimethyl-12H-furo[2′,3′:1,2]phenanthro[3,4-d]imidazol-11-yl) pyridin-1-ium bromide (****a19****).* Yield 40%. Yellow powder, m.p. 266–267 °C. IR *ν*_max_ (cm^−1^): 3423, 3037, 1623, 1587, 1523, 1346, 1146, 1024, 840, 816, 795, 678. ^**1**^**H NMR** (600 MHz, DMSO-*d*_6_) δ 13.58 (s, 1H), 10.69 (d, *J* = 9.0 Hz, 1H), 10.11 (s, 1H), 9.58 (d, *J* = 8.4 Hz, 1H), 9.19 (d, *J* = 6.0 Hz, 1H), 8.65 (d, *J* = 2.4 Hz, 1H), 8.50 (dd, *J* = 8.4, 6.0 Hz, 1H), 8.38 (d, *J* = 9.0 Hz, 1H), 8.34 (dd, *J* = 8.4, 2.4 Hz, 1H), 8.20 (d, *J* = 9.0 Hz, 1H), 8.09 (d, *J* = 1.8 Hz, 1H), 7.71 (dd, *J* = 8.4, 7.2 Hz, 1H), 7.61 (d, *J* = 9.0 Hz, 1H), 7.56 (d, *J* = 7.2 Hz, 1H), 6.32 (s, 2H). 2.79 (s, 3H), 2.66 (d, *J* = 1.2 Hz, 3H) ppm (one resonance was not observed due to active N–H of imidazole); ^**13**^**C NMR** (150 MHz, DMSO-*d*_6_) δ 149.7, 148.4, 144.5, 143.9, 142.6, 142.5, 142.4, 140.5, 136.7, 134.2, 131.2, 131.0, 130.7, 130.0, 128.7, 127.9, 127.4, 127.0, 126.7, 126.5, 123.4, 123.4, 122.8, 119.2, 118.8, 116.5, 115.4, 112.6, 63.3, 19.9, 9.8 ppm. **HRMS** (ESI-TOF) m/z Calcd for C_31_H_22_N_4_O_3_Br [M]^+^577.0870, found 577.0876. HPLC purity: 96.9%.

*4.2.2.18 3-(1,6-dimethyl-12H-furo[2′,3′:1,2]phenanthro[3,4-d]imidazol-11-yl)-1-(naphthalen-1-ylmethyl) pyridin-1-ium bromide (****a20****).* Yield 87%. Yellow powder, m.p. 266–267 °C. IR *ν*_max_ (cm^−1^): 3428, 3192, 3064, 3008, 2931, 2861, 1868, 1626, 1491, 1452, 1398, 1173, 814, 796, 774, 677. ^**1**^**H NMR** (400 MHz, DMSO-*d*_6_) δ 13.17 (s, 1H), 10.33 (d, *J* = 8.4 Hz, 1H), 9.85 (s, 1H), 9.23 (d, *J* = 8.0 Hz, 1H), 9.19 (d, *J* = 6.0 Hz, 1H), 8.35—8.25 (m, 2H), 8.17—8.10 (m, 2H), 8.06 (d, *J* = 8.0 Hz, 1H), 7.98 (d, *J* = 9.2 Hz, 1H), 7.84 (s, 1H), 7.71—7.66 (m, 3H), 7.64—7.54 (m, 2H), 7.42 (d, *J* = 7.2 Hz, 1H), 6.55 (s, 2H), 2.66 (s, 3H), 2.44 (s, 3H) ppm (one resonance was not observed due to active N–H of imidazole); ^**13**^**C NMR** (100 MHz, DMSO-*d*_6_) δ 149.4, 143.7, 142.3, 142.2, 142.0, 141.6, 136.44, 134.0, 133.7, 130.6, 130.5, 130.5, 130.4, 129.8, 129.2, 129.2, 129.0, 128.4, 127.7, 127.2, 126.8, 126.7, 126.5, 126.4, 125.8, 123.1, 122.5, 118.9, 118.7, 116.3, 115.3, 112.3, 61.4, 19.9, 9.8 ppm. **HRMS** (ESI-TOF) m/z Calcd for C_35_H_26_N_3_O [M]^+^ 504.2070, found 504.2074. HPLC purity: 98.0%.

*4.2.2.19 3-(1,6-dimethyl-12H-furo[2′,3′:1,2]phenanthro[3,4-d]imidazol-11-yl)-1-(naphthalen-2-ylmethyl) pyridin-1-ium bromide (****a21****).* Yield 65%. Yellow powder, m.p. 288–289 °C. IR *ν*_max_ (cm^−1^): 3420, 3041, 3004, 2918, 1805, 1589, 1393, 1370, 1282, 1160, 851, 816, 773, 678. ^**1**^**H NMR** (600 MHz, DMSO-*d*_6_) δ 13.57 (s, 1H), 10.65 (d, *J* = 8.4 Hz, 1H), 10.13 (s, 1H), 9.48 (d, *J* = 8.4 Hz, 1H), 9.27 (d, *J* = 6.0 Hz, 1H), 8.45—8.40 (m, 1H), 8.37 (d, *J* = 9.0 Hz, 1H), 8.26 (s, 1H), 8.18 (d, *J* = 9.0 Hz, 1H), 8.12—8.06 (m, 2H), 8.04—8.00 (m, 2H), 7.79 (d, *J* = 8.4 Hz, 1H), 7.64—7.58 (m, 3H), 7.55 (d, *J* = 7.2 Hz, 1H), 6.27 (s, 2H), 2.78 (s, 3H), 2.65 (s, 3H) ppm (one resonance was not observed due to active N–H of imidazole); ^**13**^**C NMR** (150 MHz, DMSO-*d*_6_) δ 149.6, 144.0, 143.0, 142.6, 142.5, 142.0, 136.7, 134.2, 133.1, 132.8, 131.4, 130.8, 130.6, 129.9, 129.2, 128.9, 128.5, 128.2, 127.8, 127.3, 127.2, 127.0, 126.9, 126.6, 126.5, 126.1, 122.8, 119.1, 118.8, 116.5, 115.4, 112.6, 63.9, 19.9, 9.8 ppm. **HRMS** (ESI-TOF) m/z Calcd for C_35_H_26_N_3_O [M]^+^ 504.2070, found 504.2069. HPLC purity: 96.4%.

*4.2.2.20 3-(1,6-dimethyl-12H-furo[2′,3′:1,2]phenanthro[3,4-d]imidazol-11-yl)-1-(2-methylallyl) pyridin-1-ium bromide (****a22****).* Yield 73%. Yellow powder, m.p. 290–291 °C. IR *ν*_max_ (cm^−1^): 3421, 2922, 1625, 1490, 1283, 1161, 1104, 1068, 815, 773, 680, 592. ^**1**^**H NMR** (600 MHz, DMSO-*d*_6_) δ 13.48 (s, 1H), 10.68 (d, *J* = 8.4 Hz, 1H), 9.96 (s, 1H), 9.47 (d, *J* = 7.8 Hz, 1H), 9.09 (d, *J* = 6.0 Hz, 1H), 8.41 (t, *J* = 7.2 Hz, 1H), 8.34 (d, *J* = 9.0 Hz, 1H), 8.16 (d, *J* = 9.0 Hz, 1H), 8.05 (s, 1H), 7.71 (t, *J* = 7.8 Hz, 1H), 7.53 (d, *J* = 6.6 Hz, 1H), 5.47 (s, 2H), 5.26 (s, 1H), 5.00 (s, 1H), 2.77 (s, 3H), 2.63 (s, 3H), 1.86 (s, 3H) ppm (one resonance was not observed due to active N–H of imidazole); ^**13**^**C NMR** (150 MHz, DMSO-*d*_6_) δ 149.6, 144.0, 142.9, 142.5, 142.4, 142.2, 139.6, 136.7, 134.2, 130.7, 130.6, 130.0, 128.4, 127.3, 126. 9, 126.7, 126.6, 122.7, 119.2, 118.8, 116.6, 116.4, 115.4, 112.6, 65.8, 19.9, 19.7, 9.9 ppm. **HRMS** (ESI-TOF) m/z Calcd for C_28_H_24_N_3_O [M]^+^ 418.1914, found 418.1917.

### Biological

#### Materials and cell culture

The human cancer cell lines, including breast cancer (MDA-MB-231), hepatocellular carcinoma (HepG2), and prostate cancer (22RV1) were obtained from Shanghai Institute of Biochemistry and Cell Biology, Chinese Academy of Sciences (Shanghai, China). Cells were cultured in medium supplemented with 10% fetal bovine serum (FBS), 100 units/mL penicillin and 100 mg/mL streptomycin (HyClone, Logan, UT, USA). All the cells were incubated at 37 °C, 5% CO_2_ in a humidified atmosphere.

#### Cytotoxicity assay

Cytotoxic activities were evaluated by the MTS assay. All the compounds tested were absolutely dissolved to 10 mM in dimethyl sulfoxide (DMSO) in stock. Cells (5 × 10^3^ cells/well) were plated into 96-well plates and cultured for 12 h before treatment and continuously exposed to 0.032, 0.16, 0.8, 4 and 20 µM test compounds for 48 h. Then MTS reagent (Promega, Madison, WI, USA) was added to each well, and cells were incubated at 37 °C for an additional 1–4 h and the optical density (OD) was measured at 492 nm using a microplate reader (Bio-Rad, Hercules, CA, USA). The IC_50_ values were calculated from dose–response curves.

#### Cell cycle analysis

For cell cycle analysis, cells were harvested and washed twice with phosphate-buffered saline (PBS), then fixed overnight at 4 °C in 70% ethanol. After fixation, cells were washed again and incubated with propidium iodide (PI, 50 µg/mL) in the presence of RNase A (50 µg/mL) at room temperature for 30 min. Cell cycle distribution was analyzed by flow cytometry using a FACSCalibur instrument (BD Biosciences, San Jose, CA, USA). Data analysis was performed using FlowJo software (version 10.9.0).

#### Apoptosis analysis

Cell apoptosis was analyzed by flow cytometry using an Annexin V-FITC/PI apoptosis detection kit (BD Biosciences, Franklin Lakes, NJ, USA), according to the manufacturer's instructions. Briefly, cells were seeded into 6-well plates at 3 × 10^5^ cells/well, and treated with indicated concentrations of test compounds for 48 h. Cells were harvested, washed twice with cold PBS, and resuspended in binding buffer containing Annexin V-FITC and PI. Following incubation at room temperature in the dark for 15 min, fluorescence intensity was quantified using a FACS Calibur flow cytometer (BD Biosciences, Franklin Lakes, NJ, USA).

#### Scratch wound healing assay

Cell migration rates following 24 h incubation with **a4** was quantitatively assessed using ImageJ software by measuring the wound closure area between the opposing edges.

#### PI3K activity assay

The inhibitory activity of **a4** on PI3Kα was evaluated as previously based on the production of PIP3 with the PI3-Kinase Activity ELISA kit (K-1000 s, Echelon) [25].

#### Western blot analysis

The cells (Breast cancer, MDA-MB-231) were treated with compounds for indicated time and subjected to western blot analysis as previously [25]. The antibodies against AKT (#4691), phosphor-AKT (Ser473) (#9271), S6K (#2708), phosphor-S6K (Thr389) (#9234), S6 (#9202), phospho-S6 (Ser240/244) (#88441) and PD-L1 (#13684) were all from the Cell signaling technology, and the antibody for GAPDH (MAB374) was provided by Sigma.

#### Statistical analysis

All data were expressed as means ± standard deviation (SD). Statistical analyses were performed using GraphPad Prism 9.5 software. Pairwise comparisons among groups were analyzed using Tukey's test. Differences were considered statistically significant at *p* < 0.05.

## Supplementary Information

Supplementary material 1.

## Data Availability

All relevant data are within the manuscript.
