# Supplementary material for: Synthesis and biological activity study of tanshinone I-pyridinium salt derivatives
Source: Nat Prod Bioprospect. 2025 Aug 8;15(1):51. doi: 10.1007/s13659-025-00534-7 (PMC12332164; doi:10.1007/s13659-025-00534-7)

**Supporting information**

**Synthesis and biological activity of Tanshinone l-pyridinium salt derivatives**

**Huimin Zhao^#^, Yuyang Wang^#^, Zining Liu, Lin Lin, Jiasi Xiang, Zihao Zhu, Xiongli Yang, Yongsheng Fang*, Lingmei Kong* and Yan Li***

*Key Laboratory of Medicinal Chemistry for Natural Resource, Ministry of Education; Yunnan Key Laboratory of Research and Development for Natural Products, School of Pharmacy; Yunnan University, Kunming, 650500, P. R. China.*

^†^ *These authors have contributed equally to this work.*

** Corresponding author.*

**Table of Contents**

1. General Experimental.......................................................................................................... S2

2. Experimental Procedures and Analytical Data............................................................. S3-S11

3. Biology....................................................................................................................... S12-S14

4. Molecular Docking............................................................................................................ S15

5. ^1^H NMR, ^13^C NMR and HR-MS (ESI) spectra of New Compounds........................ S16-S37

**1. General Experimental**

**1.1 Chemistry**

Melting points were obtained and uncorrected on a Haineng melting-point apparatus. Proton nuclear magnetic resonance (^1^H-NMR) spectra were recorded on a Bruker Avance 400 and Bruker Avance 600 spectrometers at 400 MHz and 600 MHz. Carbon-13 nuclear magnetic resonance (^13^C-NMR) was recorded on Bruker Avance 400 spectrometer at 100 MHz. And carbon-13 nuclear magnetic resonance (^13^C-NMR) was recorded on Bruker Avance 600 spectrometer at 150 MHz. Chemical shifts are reported as δ values in parts per million (ppm) relative to tetramethylsilane (TMS) for all recorded NMR spectra. High Resolution Mass spectra were taken on Thermo Fisher LC-MSO/TOR mass spectrometer. Silica gel (200–300 mesh) for column chromatography and silica GF254 for TLC were produced by Qingdao Marine Chemical Company (China). All air- or moisture- sensitive reactions were conducted under an argon atmosphere. Starting materials and reagents used in reactions were obtained commercially from TCI, Adamas, Aladdin, Bidepharm and were used without purification, unless otherwise indicated.

**2. Experimental Procedures and Analytical Data**

**Scheme 1.** Synthesis of Tanshinone I-Pyridinium Salts **a3-a22**.

Structures and yields of Tanshinone I-Pyridinium Salts.

| **Entry** | **Compound No.** | **R** | **Molecular**  **formula** | **Yields (%)** |
| --- | --- | --- | --- | --- |
| **1** | **a3** | Acetylphenyl | C_32_H_24_BrN_3_O_2_ | 85% |
| **2** | **a4** | 4-Bromoacetylphenyl | C_31_H_23_B_r2_N_3_O_2_ | 76% |
| **3** | **a5** | 4-Methoxyacetylphenyl | C_33_H_26_BrN_3_O_3_ | 90% |
| **4** | **a6** | 1-Acetylnaphthyl | C_36_H_26_BrN_3_O_2_ | 36% |
| **5** | **a7** | 3-Methylbenzyl | C_32_H_26_BrN_3_O | 67% |
| **6** | **a8** | 2-Cyanobenzyl | C_32_H_23_BrN_4_O | 84% |
| **7** | **a9** | 2-Bromobenzyl | C_31_H_26_Br_2_N_3_O | 69% |
| **8** | **a10** | 4-Bromobenzyl | C_31_H_23_Br_2_N_3_O | 89% |
| **9** | **a11** | 4-Chlorobenzyl | C_31_H_23_BrClN_3_O | 92% |
| **10** | **a12** | 4-Fluorobenzyl | C_31_H_23_BrFN_3_O | 97% |
| **11** | **a13** | 4-(Trifluoromethyl)benzyl | C_32_H_23_BrF_3_N_3_O | 85% |
| **12** | **a14** | 4-(Formyl)benzyl | C_32_H_24_BrN_3_O_2_ | 74% |
| **13** | **a15** | 4-Methylbenzyl | C_32_H_26_BrN_3_O | 60% |
| **14** | **a16** | 3,4-Dichlorobenzyl | C_31_H_22_BrCl_2_N_3_O | 79% |
| **15** | **a17** | 3-Fluoro-4-nitrobenzyl | C_31_H_22_BrFN_4_O_3_ | 61% |
| **16** | **a18** | 3-Methyl-4-nitrobenzyl | C_32_H_25_BrN_4_O_3_ | 47% |
| **17** | **a19** | 2-Bromo-4-nitrobenzyl | C_31_H_22_Br_2_N_4_O_3_ | 40% |
| **18** | **a20** | Naphthalen-1-ylmethyl | C_35_H_26_BrN_3_O | 87% |
| **19** | **a21** | Naphthalen-2-ylmethyl | C_35_H_26_BrN_3_O | 65% |
| **20** | **a22** | 2-Methylallyl | C_28_H_24_BrN_3_O | 73% |

**2.1 Synthesis of compounds a2**

Compound **a1** (0.10g, 0.36 mmol) was charged into a 50 mL microwave tube and dissolved in analytical-grade acetic acid (20 mL). Ammonium acetate (0.11 g, 1.45 mmol) was added, followed by the dropwise addition of 3-pyridinecarbaldehyde (0.051 mL, 0.54 mmol) under room temperature conditions. The mixture was stirred at room temperature for 10 min, then heated to 100 ^o^C in a microwave reactor and maintained for 1 hour. After the reaction mixture was allowed to cool to room temperature, the solution was transferred into a 100 mL round-bottom flask and adjusted to neutral pH with saturated aqueous NaOH solution. The neutralized mixture was extracted with ethyl acetate (3 × 30 mL), and the aqueous phase was further extracted with ethyl acetate (3× 30 mL). The combined organic layers were dried over anhydrous Na_2_SO_4_, filtered, and concentrated under reduced pressure. The crude product was purified by column chromatography (silica gel, eluent: dichloromethane/ethyl acetate = 10:1 → 5:1, v/v, containing 1% (v/v) formic acid) to afford **a2** as yellow powder in 76% yield.

**2.1.1 Experimental findings & Structural elucidation**

According to the Debus-Radziszewski imidazole synthesis mechanism and experimental results, both a major product and a side product were observed. After separation and purification, NMR analysis could not unambiguously confirm whether the major product was **a2** or **s1**. The final structural assignment was achieved via single-crystal X-ray diffraction, which definitively identified the compound as **a2**.


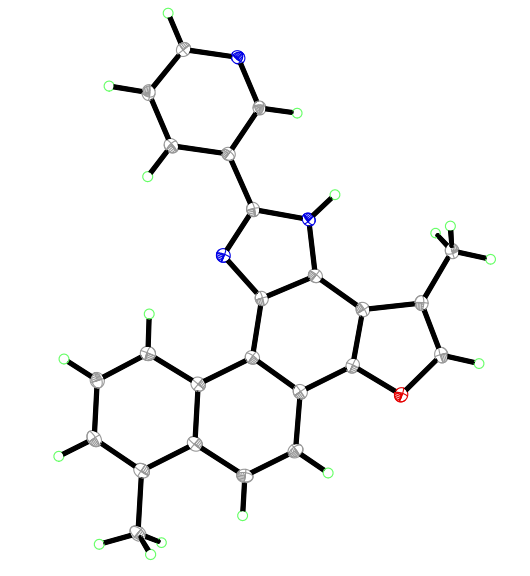


**Figure 1.** Single-crystal X-ray diffraction structure of Compound **a2**

**2.2 Synthesis of compounds a3-a22**

Compound **a2** (0.05 g, 0.14 mmol) was charged into a 50 mL round-bottom flask and dissolved in ultra-dry acetonitrile (20 mL). The mixture was heated under reflux at 100 ^o^C for 12 hours until complete dissolution of **a2** was achieved. Subsequently, various bromides (5.0 equiv relative to **a2**, molar ratio of substrate : bromide = 1:5) were added sequentially with stirring (ultra-dry acetonitrile volume: 20 mL per 50 mg substrate). The reaction was maintained under reflux with stirring for 24–48 hours and monitored by TLC. Upon completion, the solvent was removed under reduced pressure, and the precipitated solid was collected by filtration. The crude product was washed thoroughly with ethyl acetate (3 × 60 mL) and dried under vacuum at 60 ^o^C to afford Tanshinone I-pyridinium derivatives **a3–a22** as yellow powder in 36–97% yields.

**2.2.1 Structural assignment of the quaternization site**

During the quaternization reaction described above, ambiguity persisted regarding the regioselectivity of bromide coupling—specifically, whether the reaction occurred at the imidazole or pyridine ring. Taking **a4** as a representative example, this uncertainty was resolved through comprehensive analysis of **2D NMR spectra** (e.g., ^1^*H*-^13^*C* HSQC, HMBC), which conclusively demonstrated that quaternization proceeded exclusively at the pyridine ring.

In the aforementioned reaction system where compounds **a4** and **s2** could potentially form, structural elucidation was performed through comprehensive 2D NMR analysis. Initial HSQC correlation analysis identified the chemical shifts of Ha and Ca at 6.68 ppm (¹H) and 66.83 ppm (¹³C) respectively (**Figure 2**). Subsequent determination of Cb and Cc chemical shifts was required to verify salt formation between the bromide and pyridine moieties through coupling verification of the 6.68 ppm proton.

Distinctive singlet patterns for Hb and Hd in both compounds prompted HMBC analysis. A correlation between the 8.09 ppm proton and the 9.79 ppm carbon enabled assignment of Hd, leaving the 9.98 ppm singlet attributable to Hb (**Figure 3**). Subsequent HSQC correlation established Cb at 144.58 ppm. Final confirmation was achieved through HMBC analysis demonstrating: 1) a 9.98 ppm (Hb) to 66.83 ppm (Ca) correlation, and 2) a 6.68 ppm (Ha) to 144.58 ppm (Cb) correlation, thereby conclusively identifying the product as compound **a4**.


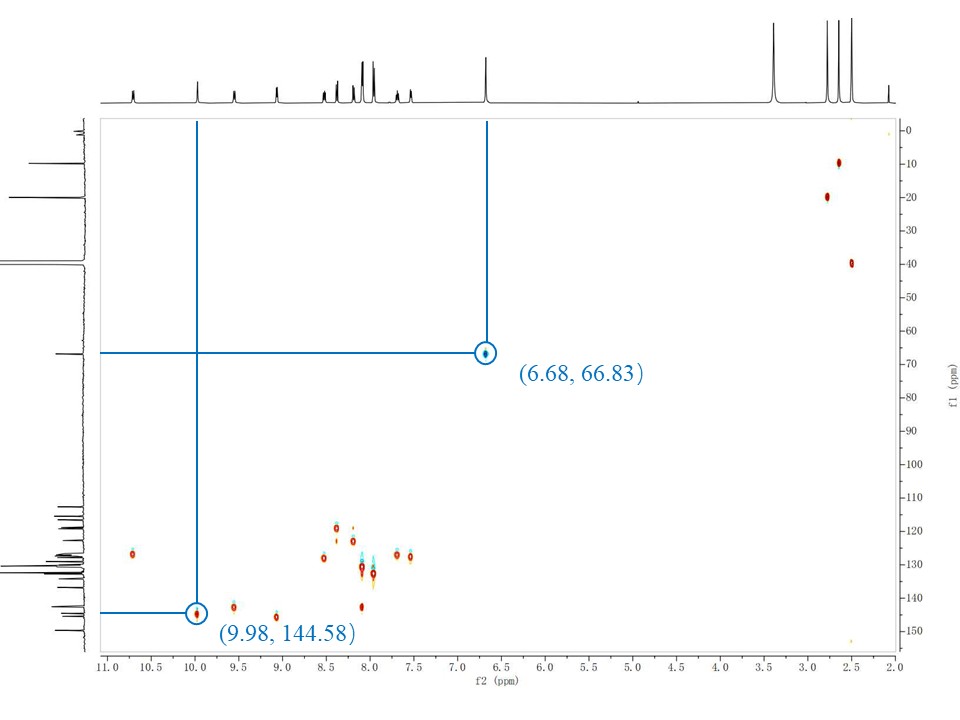


**Figure 2.** HSQC spectrum of Compound **a4**


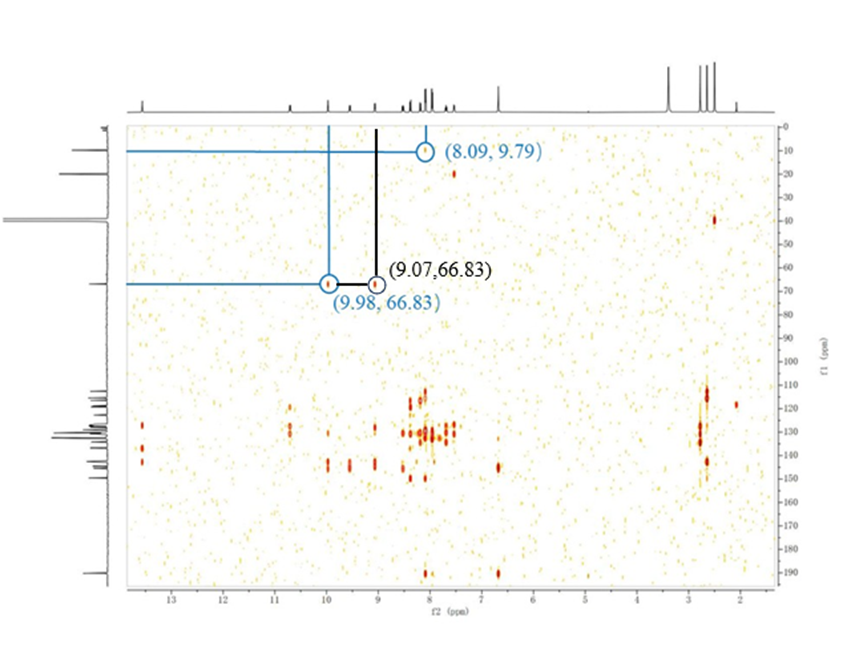


**Figure 3.** HMBC spectrum of Compound **a4**

**2.3 Preliminary Analysis of Compounds Water Solubility**

The aqueous solubility of compound **a2** was preliminarily determined using ultraviolet (UV) spectrophotometry. Initially, 2.0 mg of compound **a2** was accurately weighed and dissolved in Phosphate Buffer Saline (PBS) to prepare a standard stock solution with a concentration of 200 μg/mL. This stock solution was subsequently diluted to generate a series of standard working solutions ranging from 5 to 40 μg/mL. Using PBS as the blank, the absorbance of these standards was measured at a wavelength of 270 nm to construct a calibration curve. Next, an excess amount of compound **a2** was added to 2 mL of PBS. This mixture was sonicated for 30 minutes in a constant-temperature water bath maintained at room temperature and then allowed to stand undisturbed for 24 hours to achieve saturation equilibrium. Following this, the supernatant was carefully collected. A 1.0 mL aliquot of the supernatant was diluted 3-fold with PBS. The absorbance of this diluted saturated solution was measured at 270 nm. The measured absorbance value was interpolated into the calibration curve to determine its concentration. This concentration was then converted to calculate the aqueous solubility of the compound. The results indicated that the solubility of compound **a2** in PBS was approximately 32.0 μg/mL.

Following the identical methodology described above, the aqueous solubilities of the highly active compound **a4** and the moderately active compound **a16** were determined in PBS. The results revealed that the solubility of compound **a4** was approximately 84.5 μg/mL, while the solubility of compound **a16** was approximately 85.5 μg/mL.

Preliminary analysis of the data indicates that the solubility of the precursor compound **a2** was significantly enhanced compared to the sparingly soluble parent compound Tanshinone I. Furthermore, upon salt formation, compounds **a4** and **a16** exhibited optimal aqueous solubility. Specifically, their solubilities in PBS were approximately 2.6-fold higher than that of the precursor compound **a2**. (The entire experimental procedure was conducted with light protection, and all data points represent the average of triplicate measurements.)

**3 Biology**

**3.1 Materials and Cell Culture**

The human cancer cell lines, including breast cancer (MDA-MB-231), hepatocellular carcinoma (HepG-2), and prostate cancer (22RV1) were obtained from Shanghai Institute of Biochemistry and Cell Biology, Chinese Academy of Sciences (Shanghai, China). Cells were cultured in medium supplemented with 10% fetal bovine serum (FBS), 100 units/mL penicillin and 100 mg/mL streptomycin (HyClone, Logan, UT, USA). All the cells were incubated at 37 °C, 5% CO_2_ in a humidified atmosphere.

**3.2 Cytotoxicity assay**

Cytotoxic activities were evaluated by the MTS assay. All the compounds tested were absolutely dissolved to 10 mM in dimethyl sulfoxide (DMSO) in stock. Cells (5×10^3^ cells/well) were plated into 96-well plates and cultured for 12 h before treatment and continuously exposed to 0.032, 0.16, 0.8, 4 and 20 µM test compounds for 48 h. Then MTS reagent (Promega, Madison, WI, USA) was added to each well, and cells were incubated at 37 °C for an additional 1-4 h and the optical density (OD) was measured at 492 nm using a microplate reader (Bio-Rad, Hercules, CA, USA). The IC_50_ values were calculated from dose-response curves.

**3.3 Cell cycle analysis**

For cell cycle analysis, cells were harvested and washed twice with phosphate-buffered saline (PBS), then fixed overnight at 4 °C in 70% ethanol. After fixation, cells were washed again and incubated with propidium iodide (PI, 50 µg/mL) in the presence of RNase A (50 µg/mL) at room temperature for 30 min. Cell cycle distribution was analyzed by flow cytometry using a FACSCalibur instrument (BD Biosciences, San Jose, CA, USA). Data analysis was performed using FlowJo software (version 10.9.0).

**3.4 Apoptosis analysis**

Cell apoptosis was analyzed by flow cytometry using an Annexin V-FITC/PI apoptosis detection kit (BD Biosciences, Franklin Lakes, NJ, USA), according to the manufacturer's instructions. Briefly, cells were seeded into 6-well plates at 3×10^5^ cells/well, and treated with indicated concentrations of test compounds for 48 h. Cells were harvested, washed twice with cold PBS, and resuspended in binding buffer containing Annexin V-FITC and PI. Following incubation at room temperature in the dark for 15 min, fluorescence intensity was quantified using a FACSCalibur flow cytometer (BD Biosciences, Franklin Lakes, NJ, USA).

**3.5 Western blot analysis**

The cells were treated with compounds for indicated time and subjected to western blot analysis as previously. The antibodies against AKT, phosphor-AKT (Ser473), S6K, phosphor-S6K (Thr389), S6, phospho-S6 and PD-L1were all from the Cell signaling technology, and the antibody for GAPDH was provided by Sigma.

**3.6 Statistical analysis**

All data were expressed as means ± standard deviation (SD). Statistical analyses were performed using GraphPad Prism 9.5 software. Pairwise comparisons among groups were analyzed using Tukey's test. Differences were considered statistically significant at *p* < 0.05.

**4 Molecular Docking**

In order to provide a reasonable explanation for the excellent activity of compound **a4**, a molecular docking study was performed using Autodock vina.


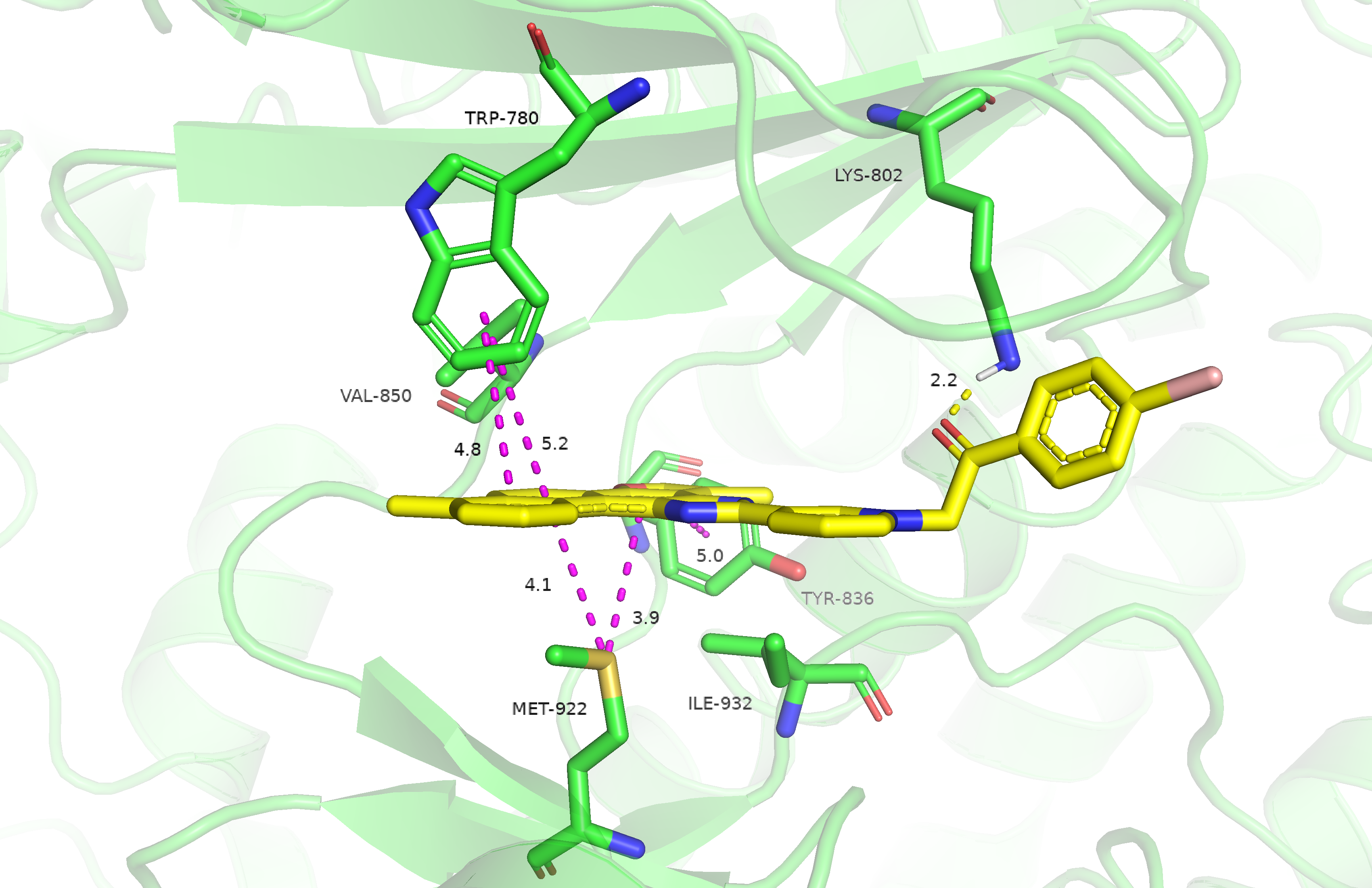

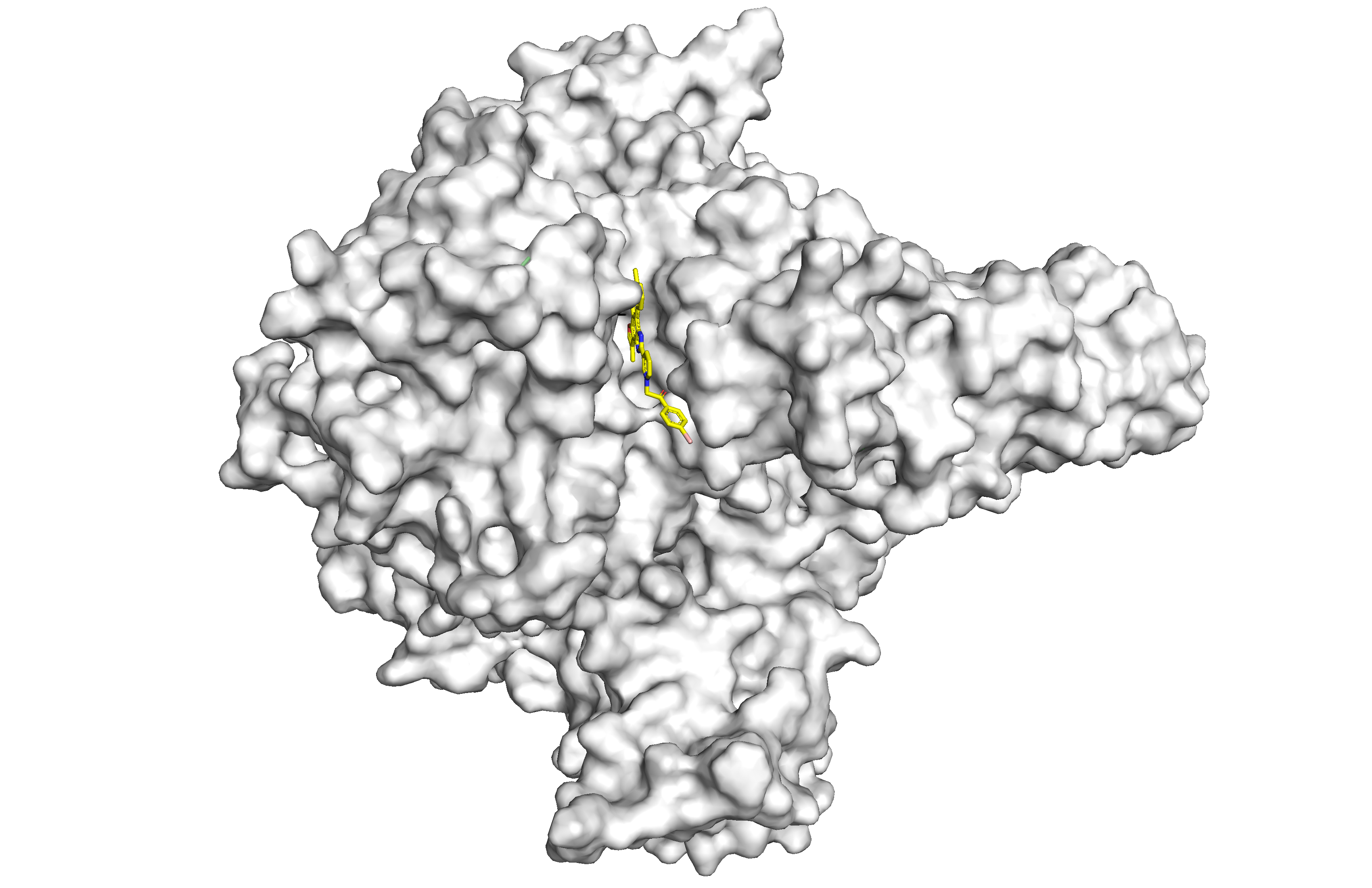


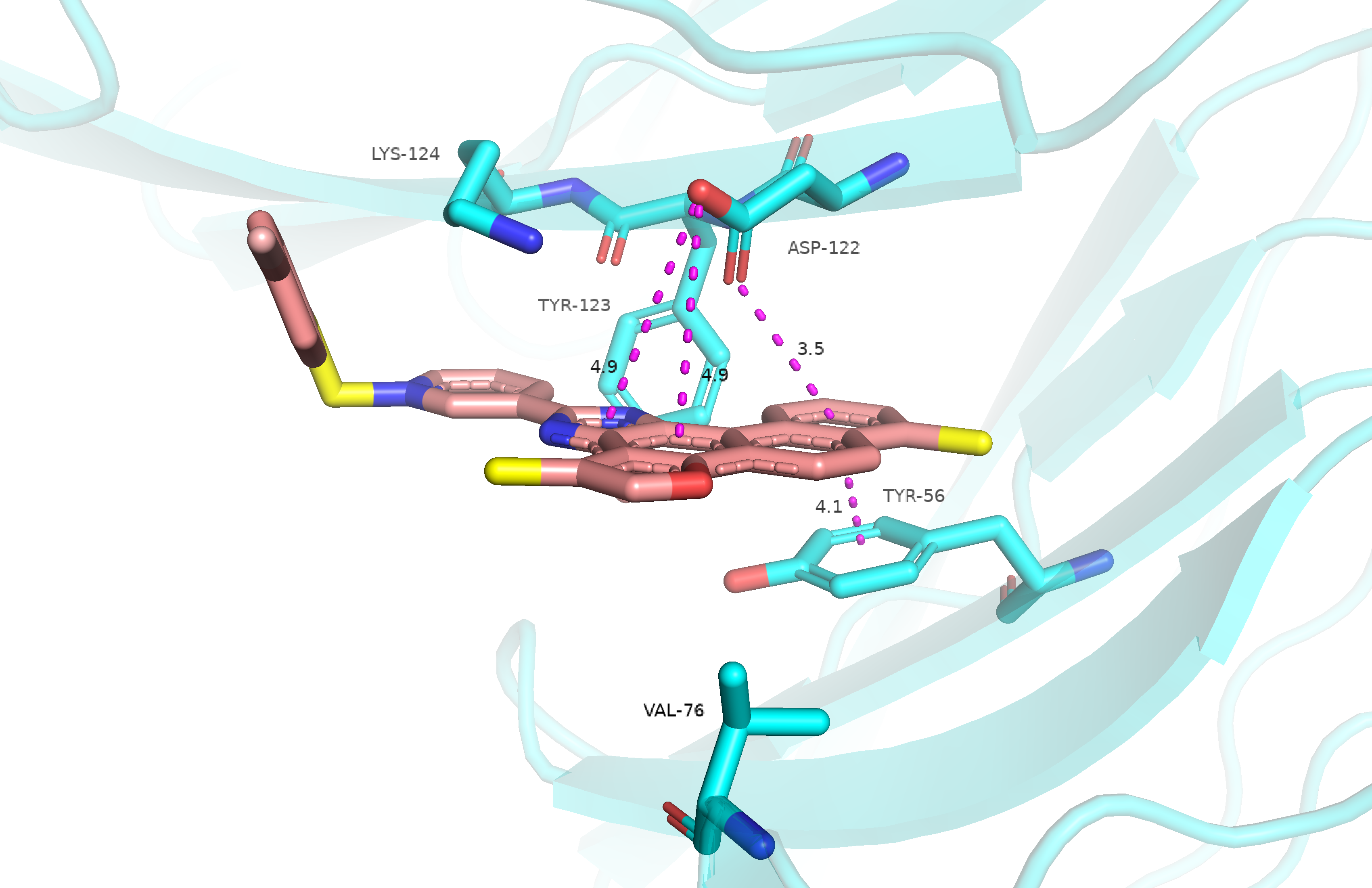

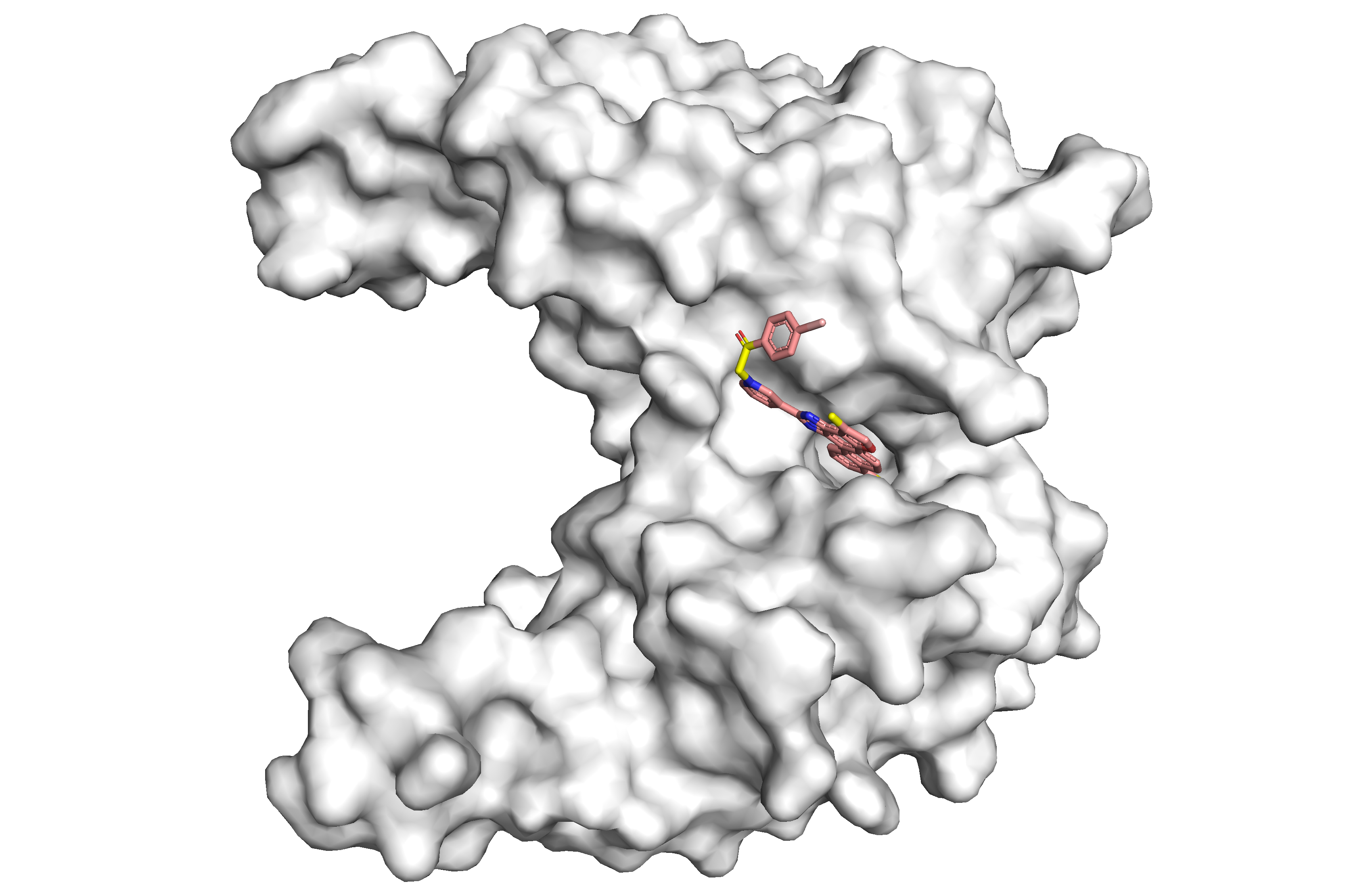


Figure showed the binding site of compound **a4** in PI3Kα protein (PDB ID: **4JPS**), we can also find a hydrogen bond in interaction diagram. The conjugated polycyclic structure of the compound **a4** plays a significant role in molecular binding, under the interaction with the conjugated structures of the protein residues, three types of π-π stacking interactions were observed. Additionally, the sulfur atoms within the residues also play a role in binding through hydrophobic interaction.

**5. Copies of 1H NMR, 13C NMR and HR-MS (ESI) spectra**

^1^H NMR, ^13^C NMR spectra of compound **a2**


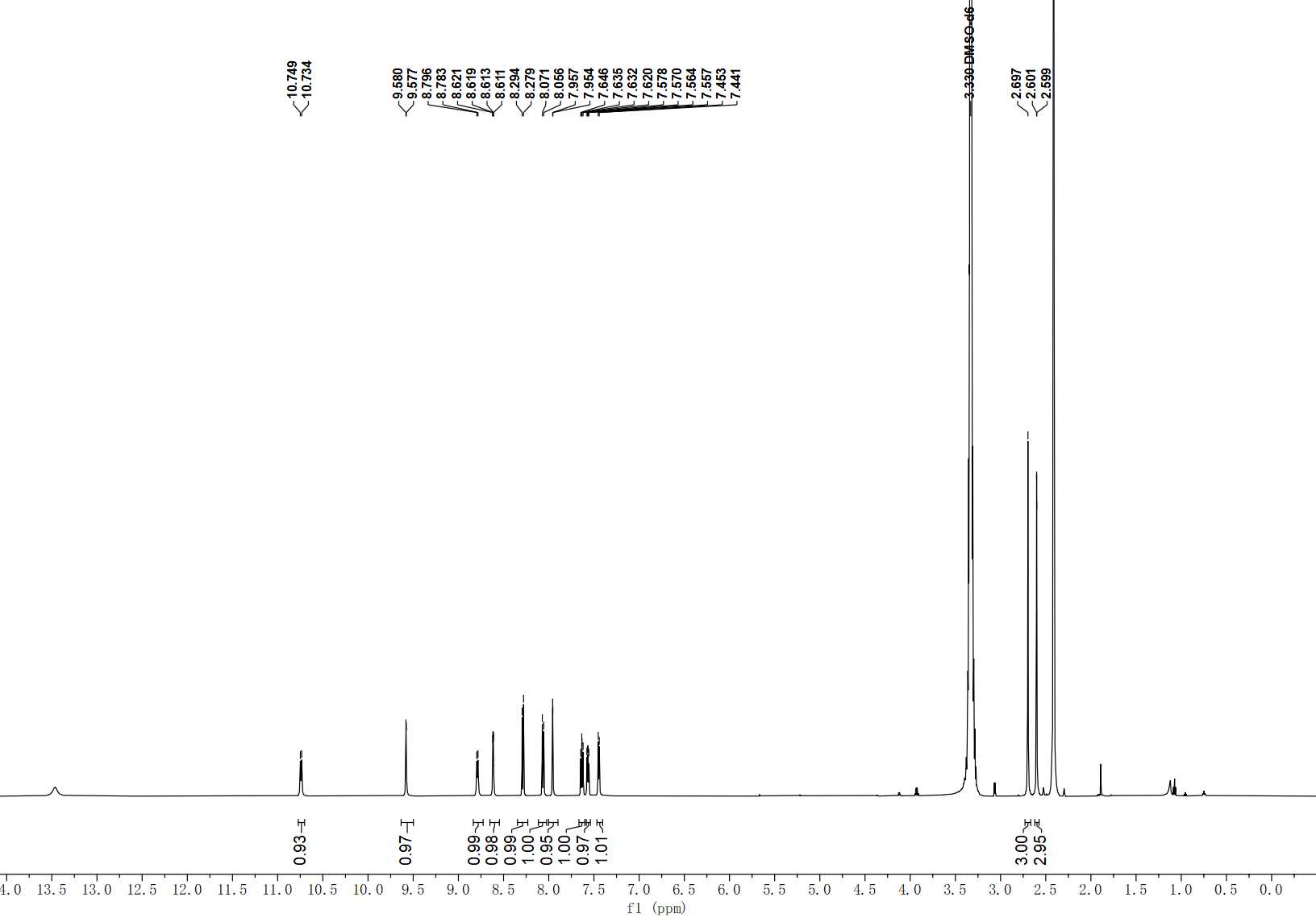


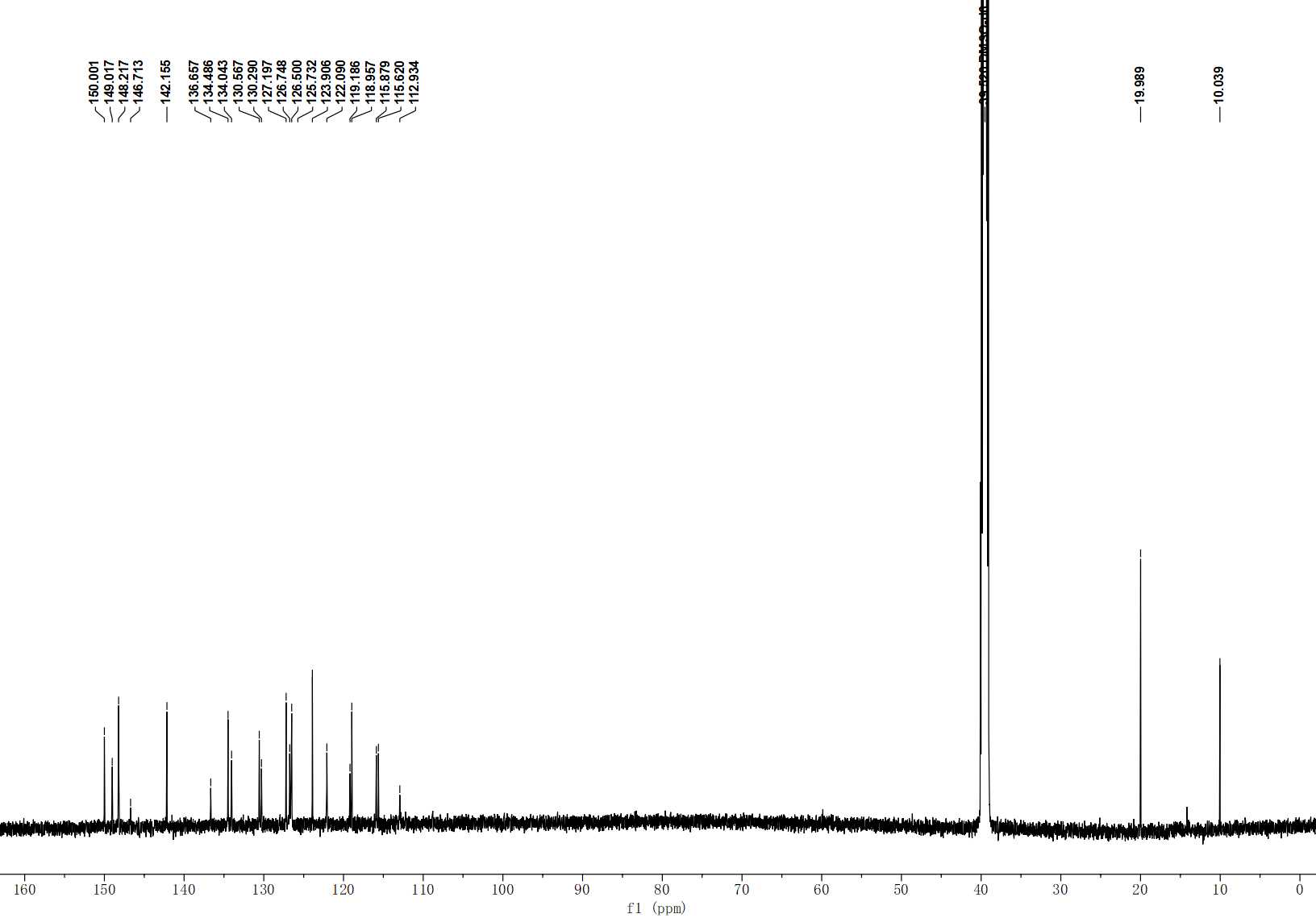


^1^H NMR, ^13^C NMR spectra of compound **a3**


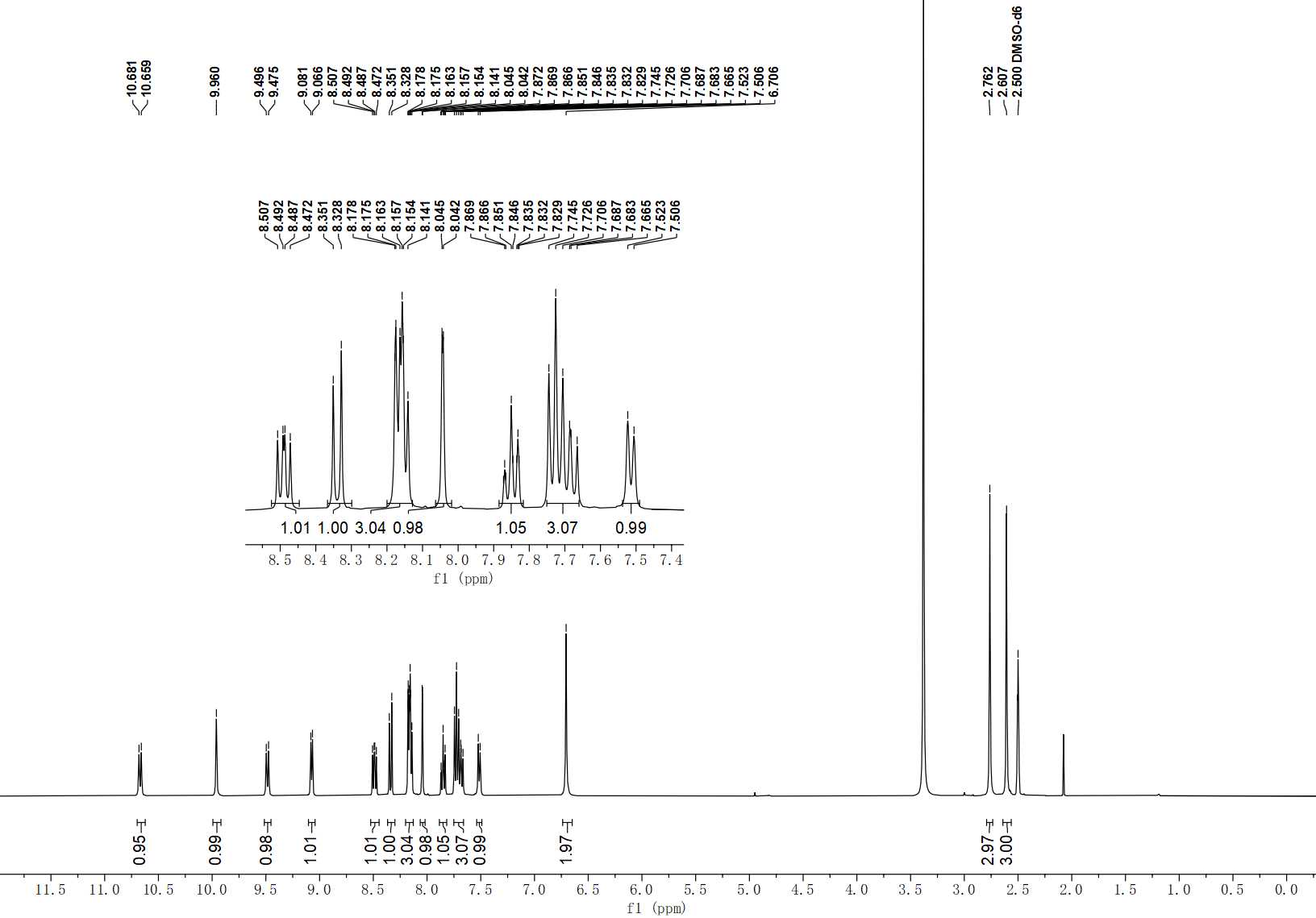


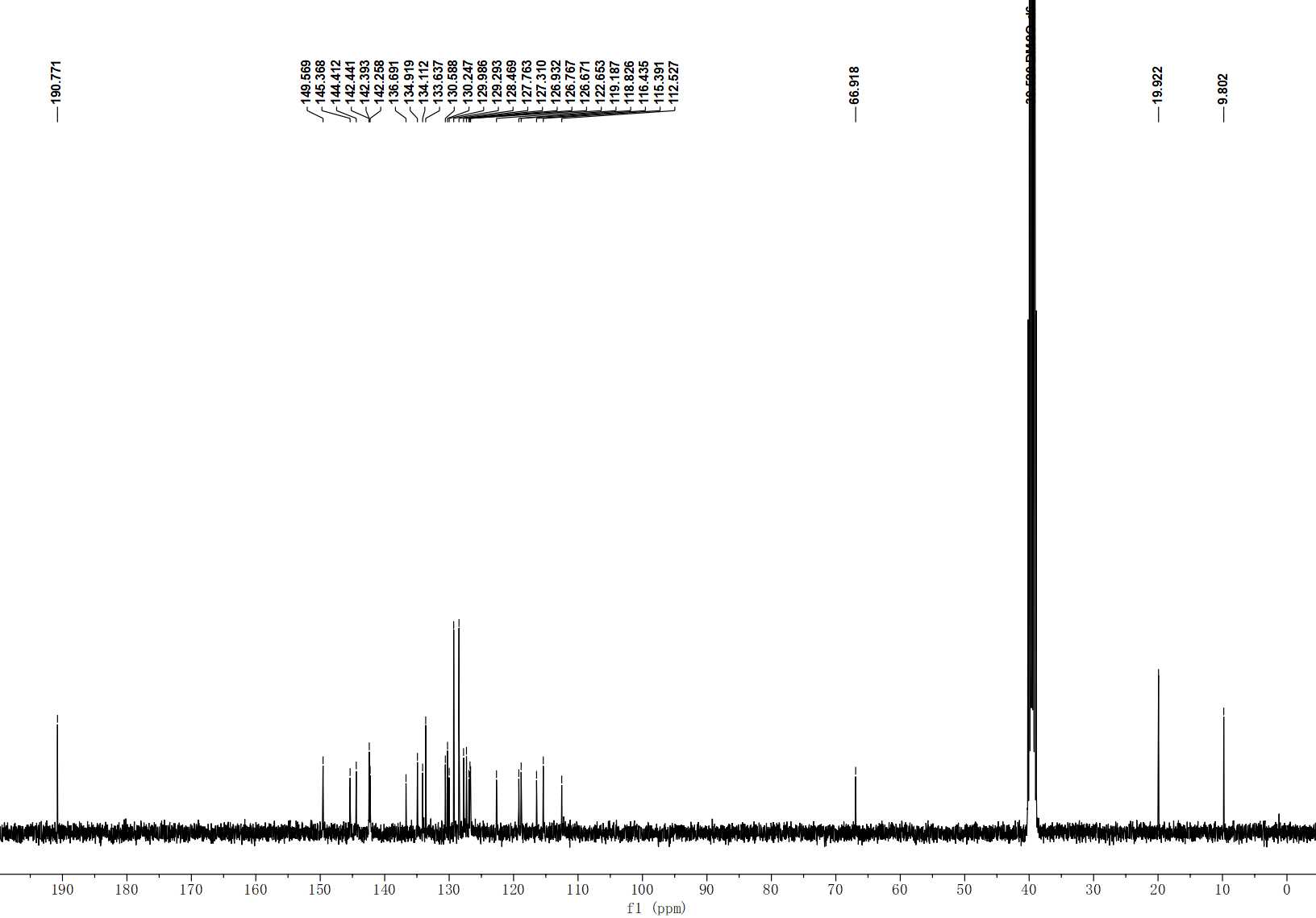


^1^H NMR, ^13^C NMR spectra of compound **a4**


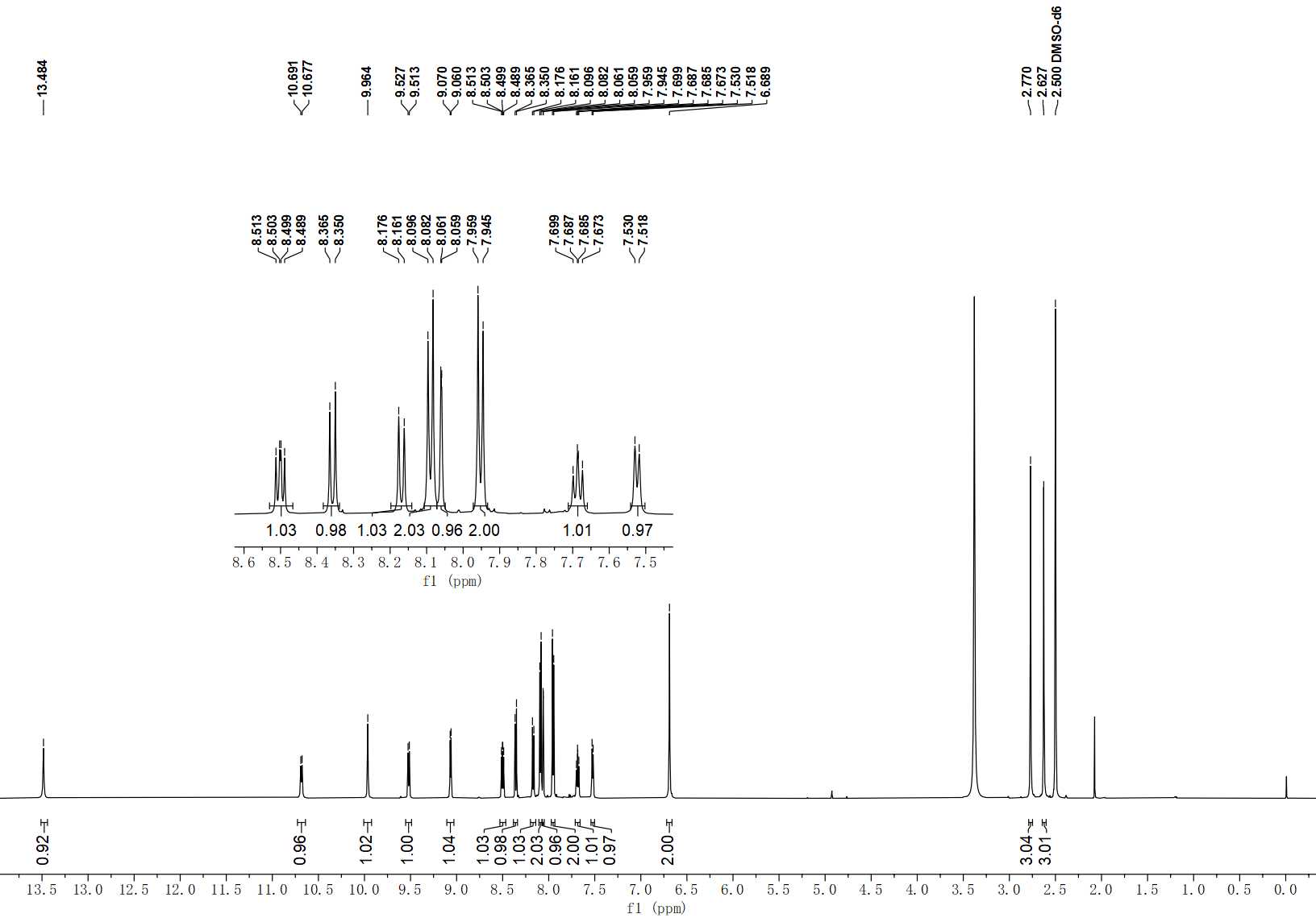


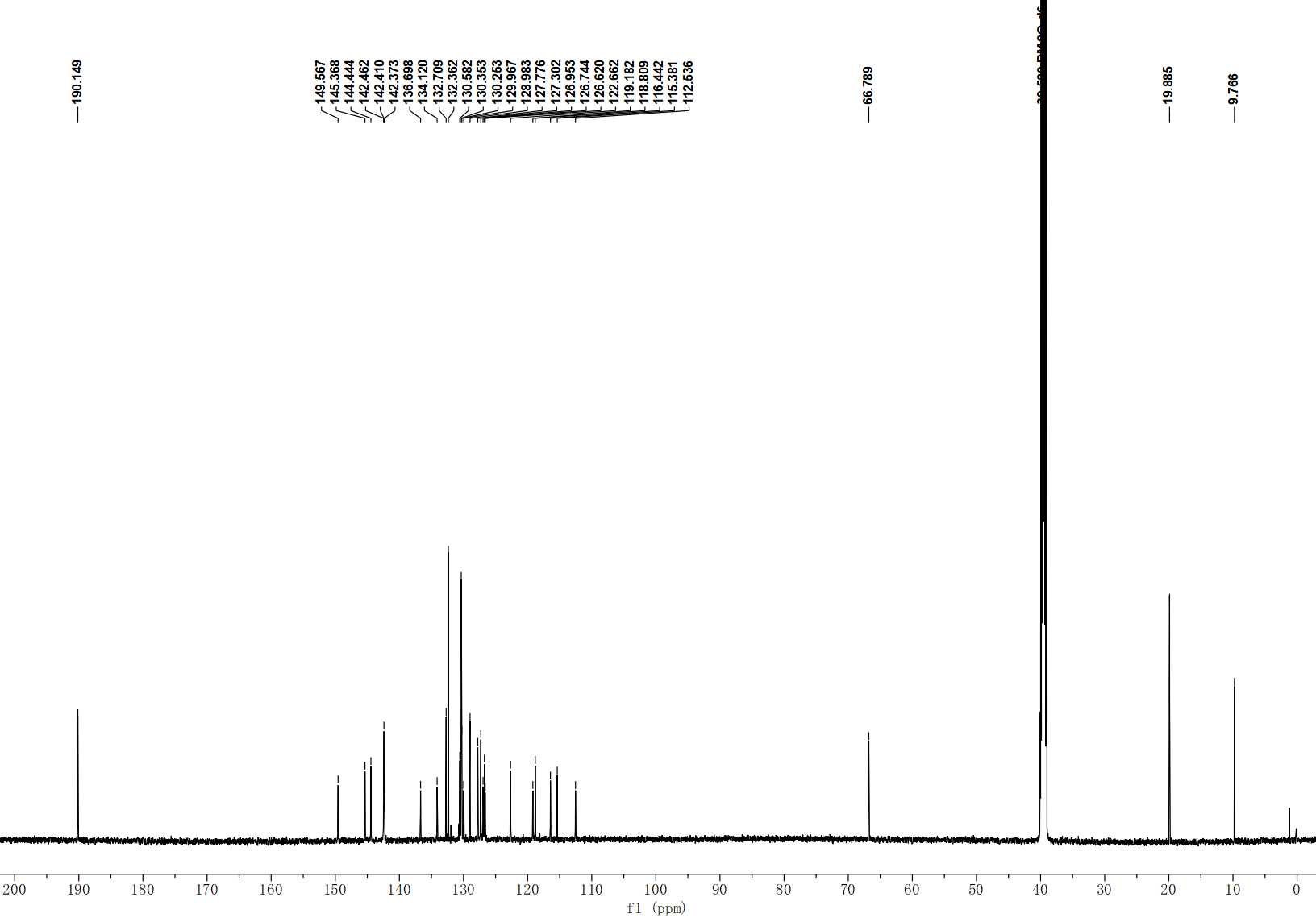


HR-MS (ESI) spectra and HPLC purity of compound **a4**


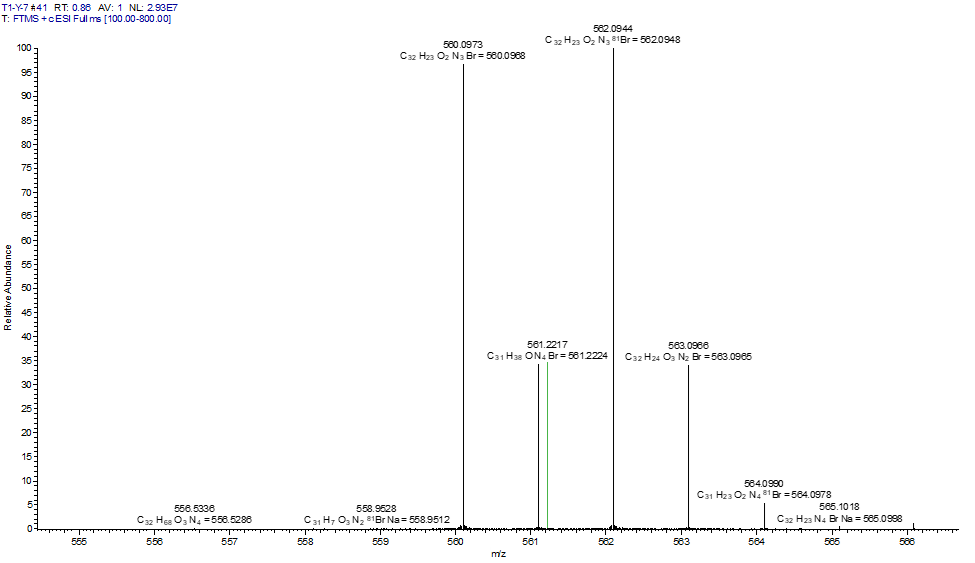


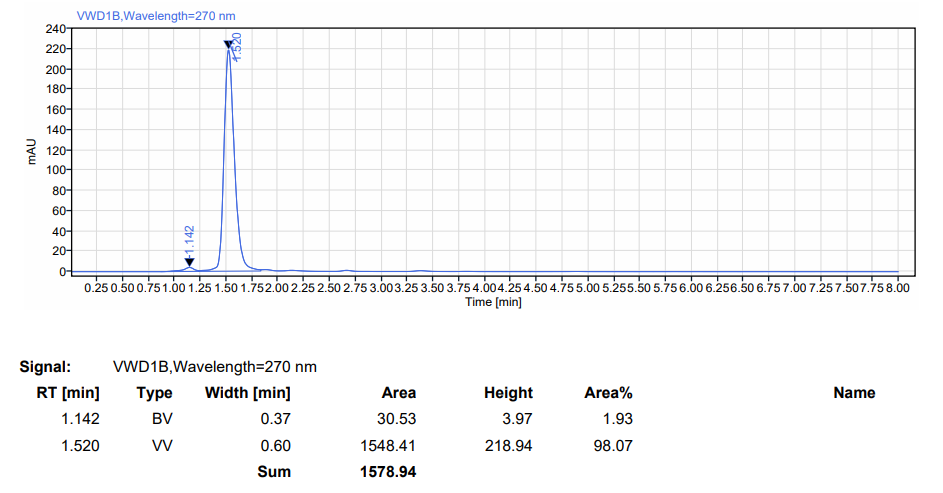


^1^H NMR, ^13^C NMR spectra of compound **a5**


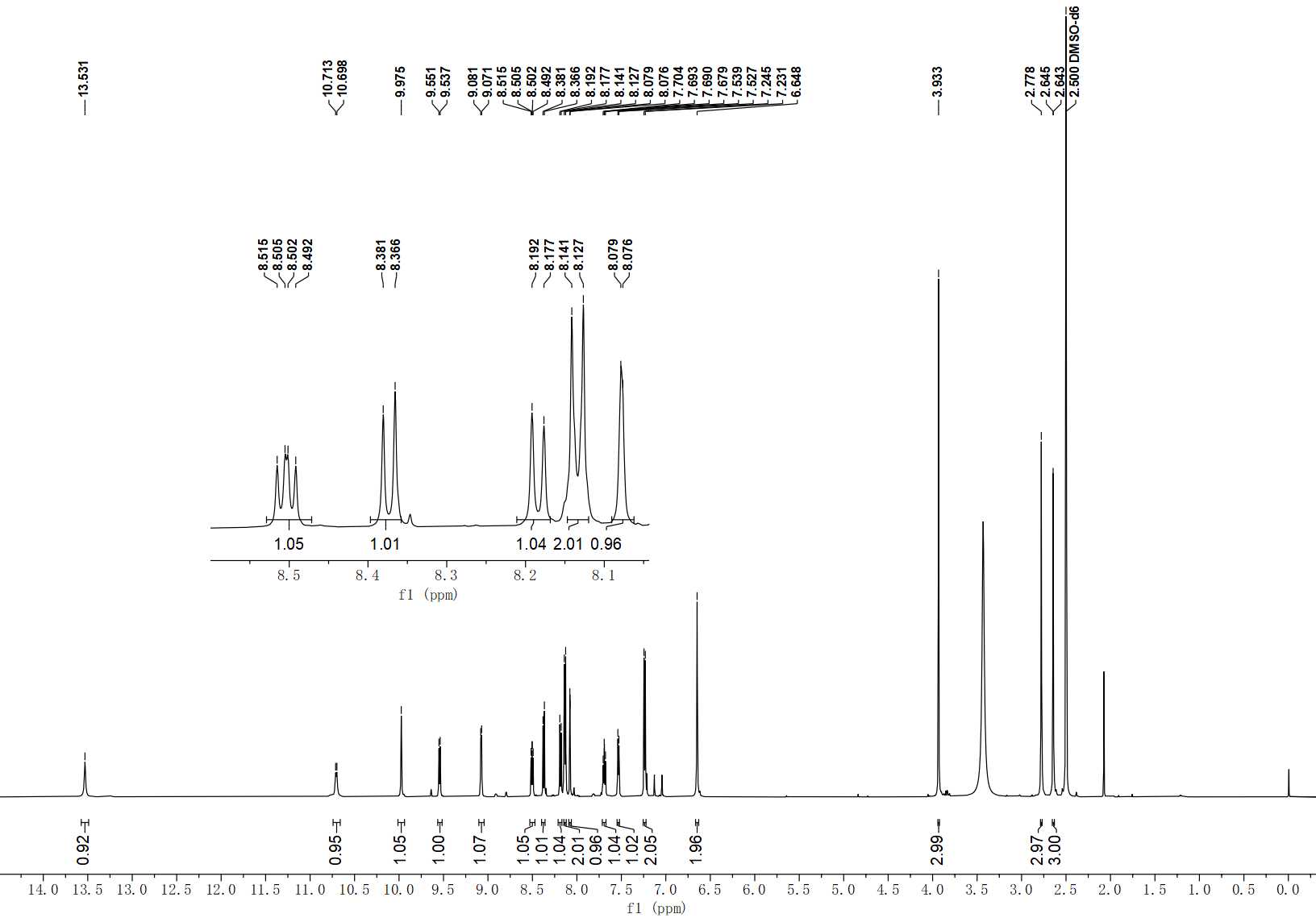


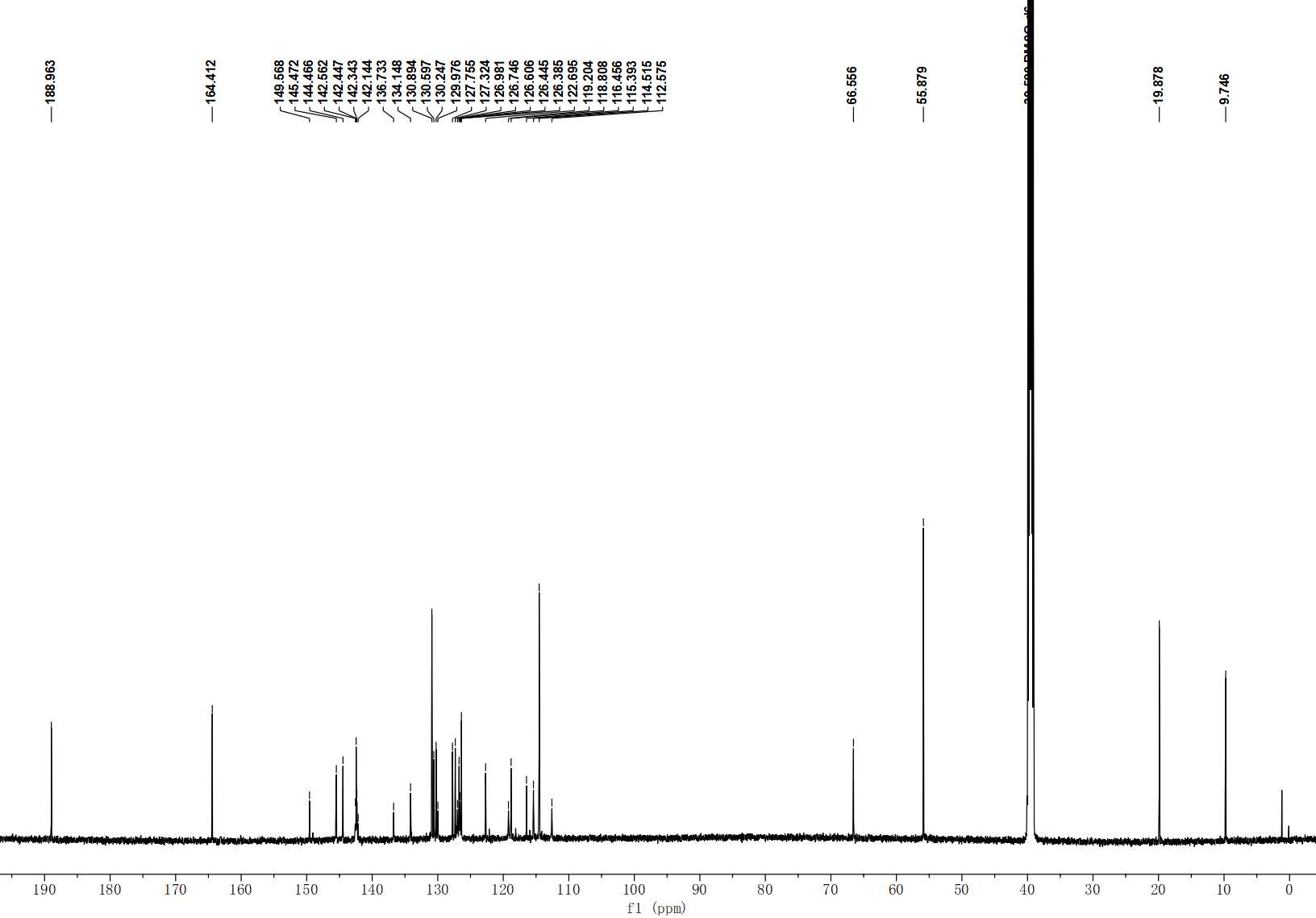


^1^H NMR, ^13^C NMR spectra of compound **a6**


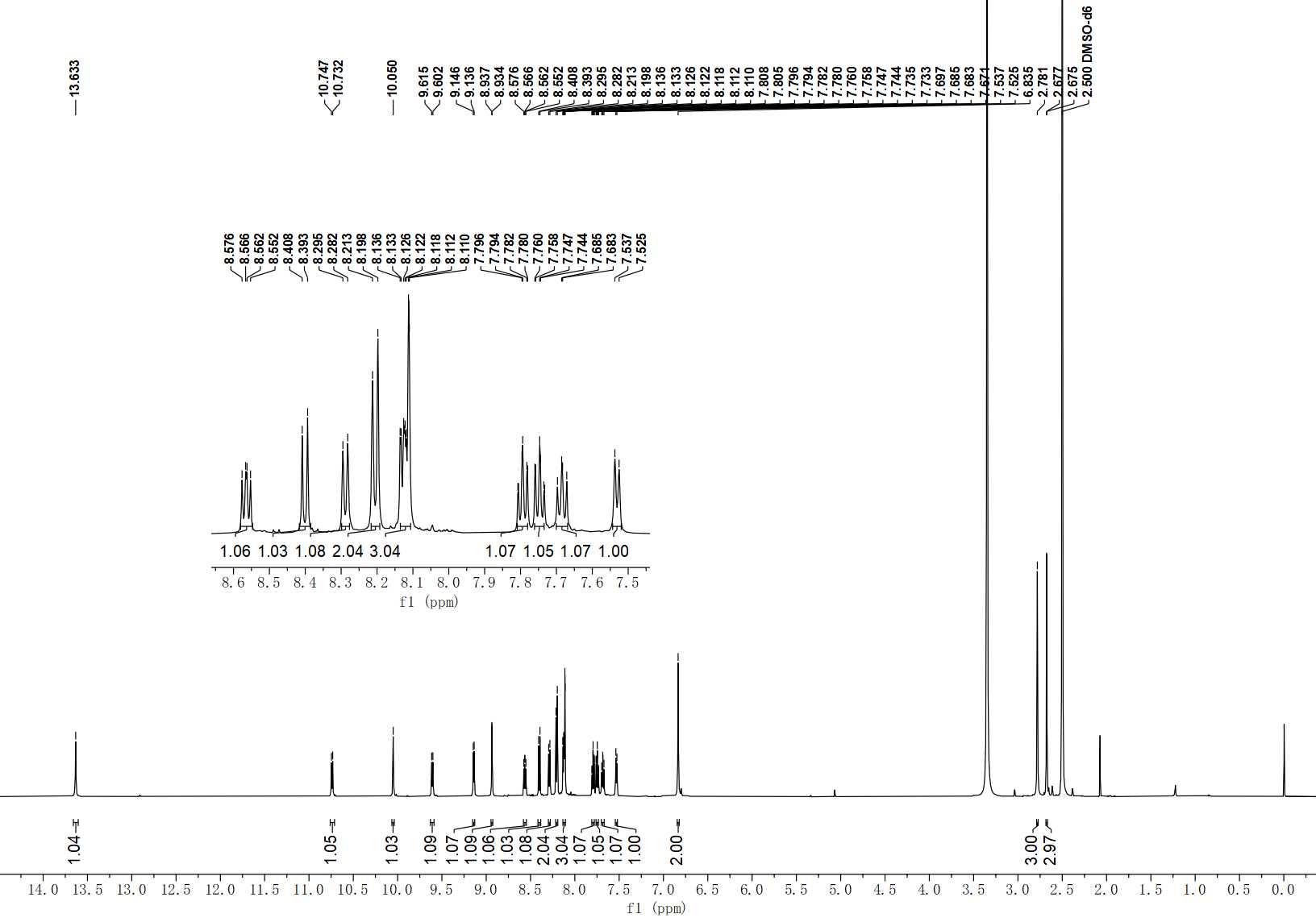


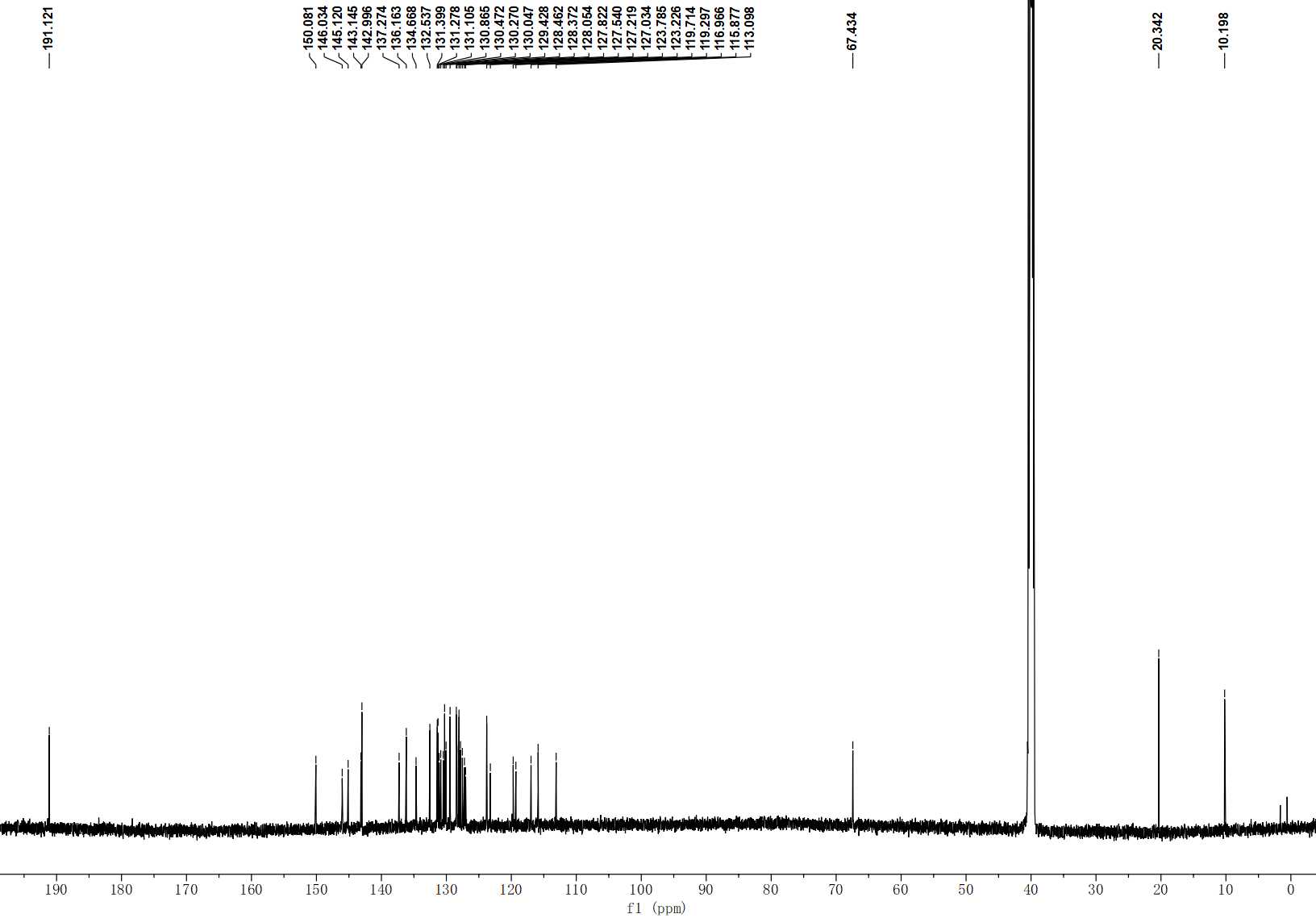


^1^H NMR, ^13^C NMR spectra of compound **a7**


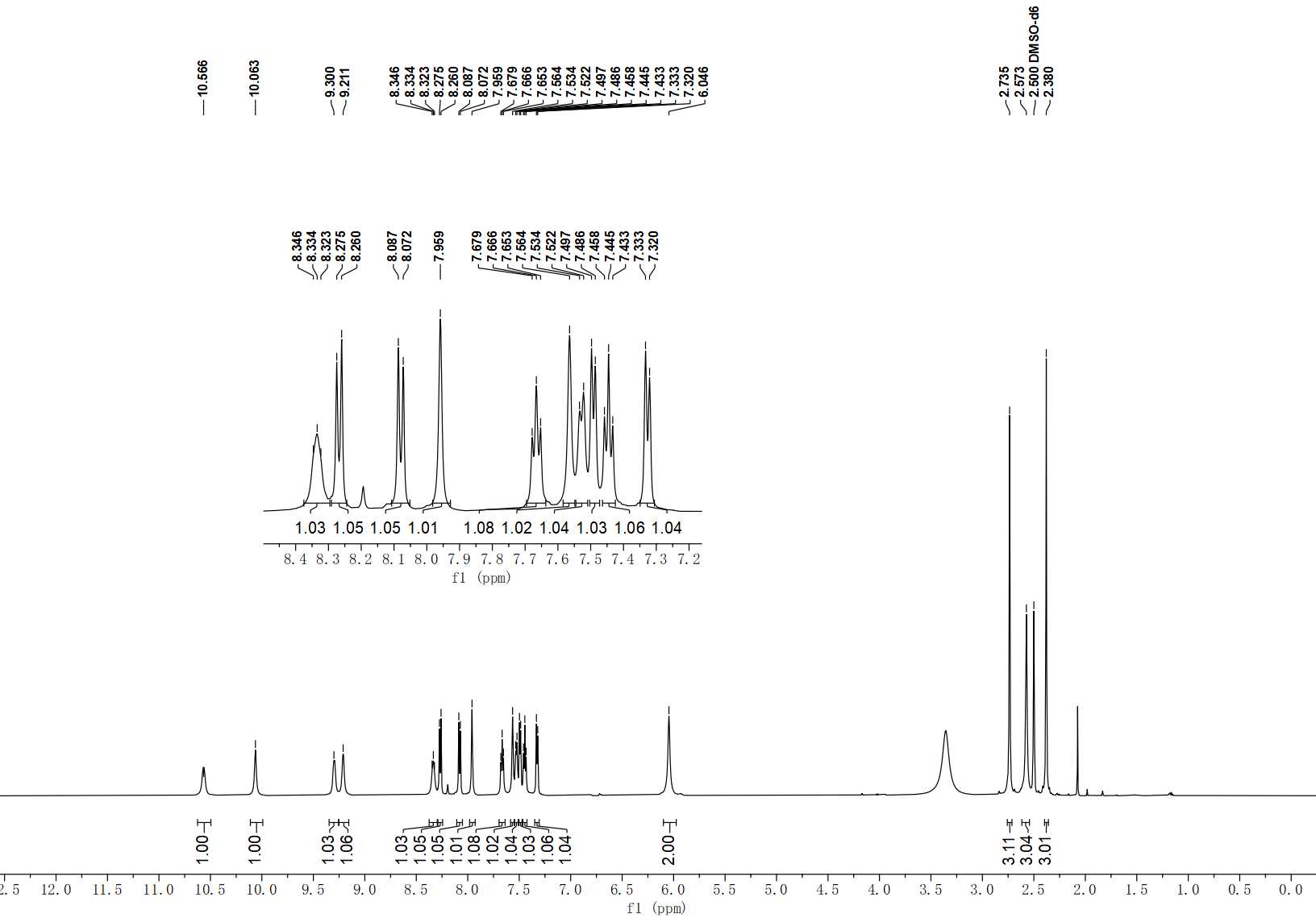


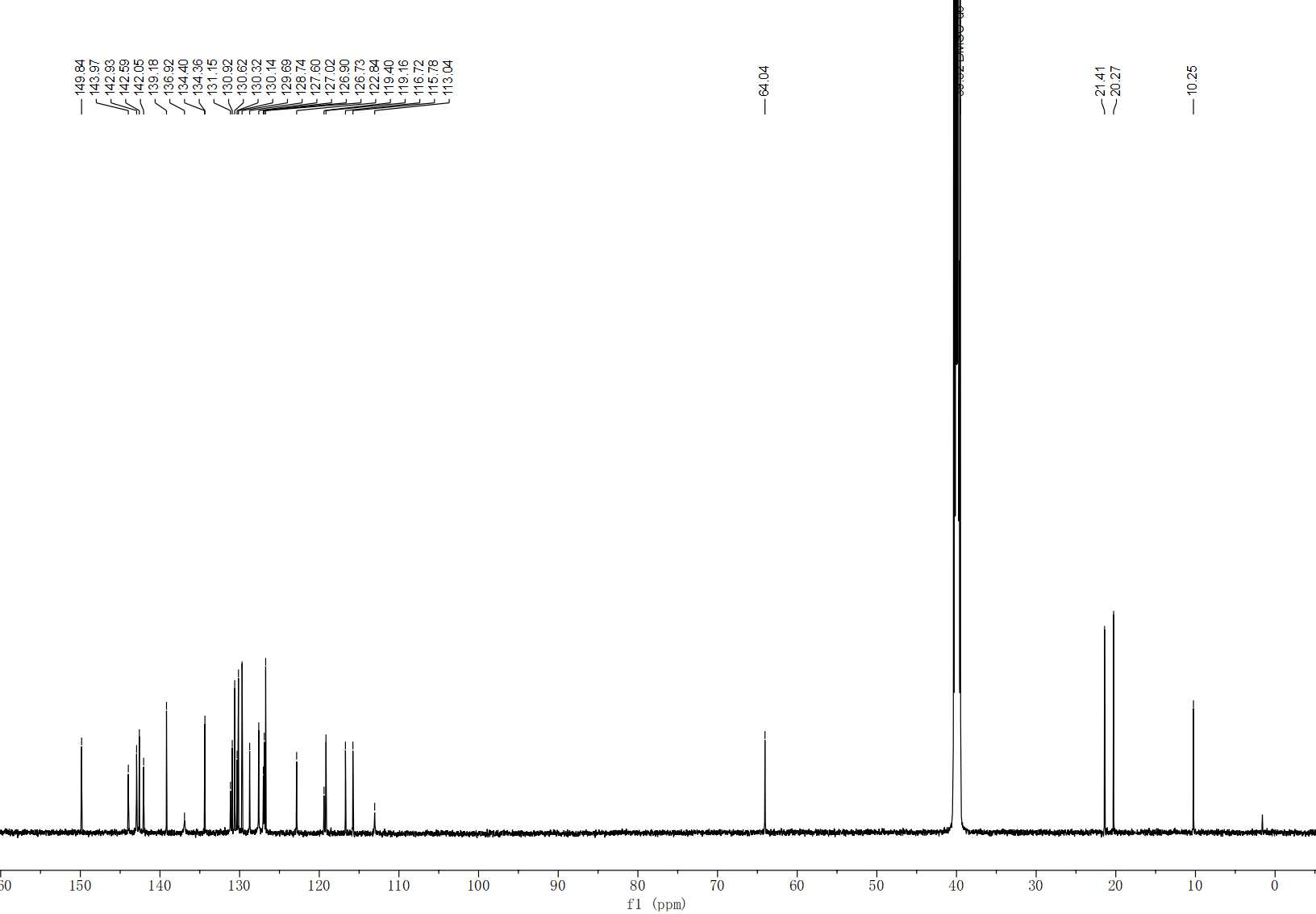


^1^H NMR, ^13^C NMR spectra of compound **a8**


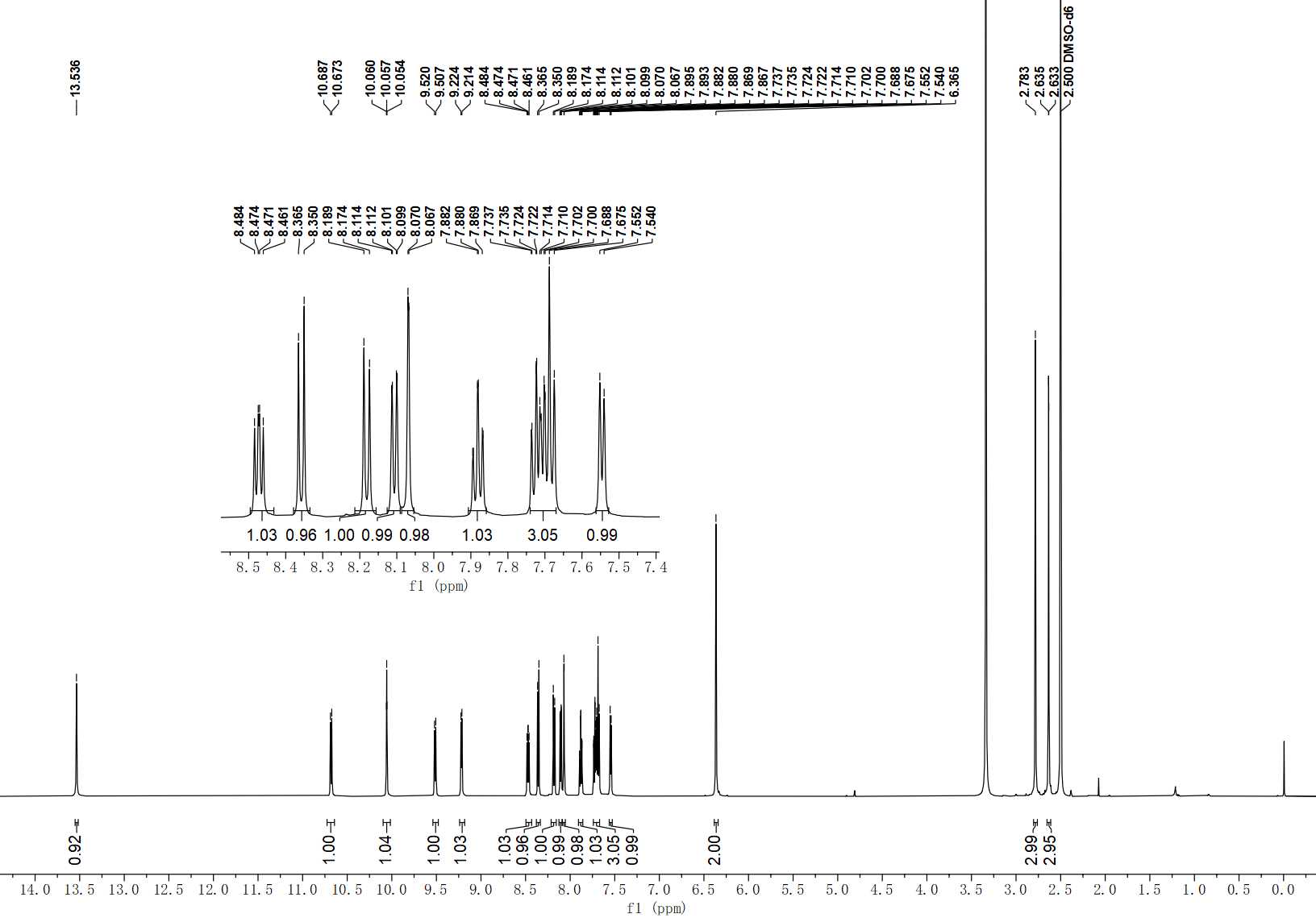


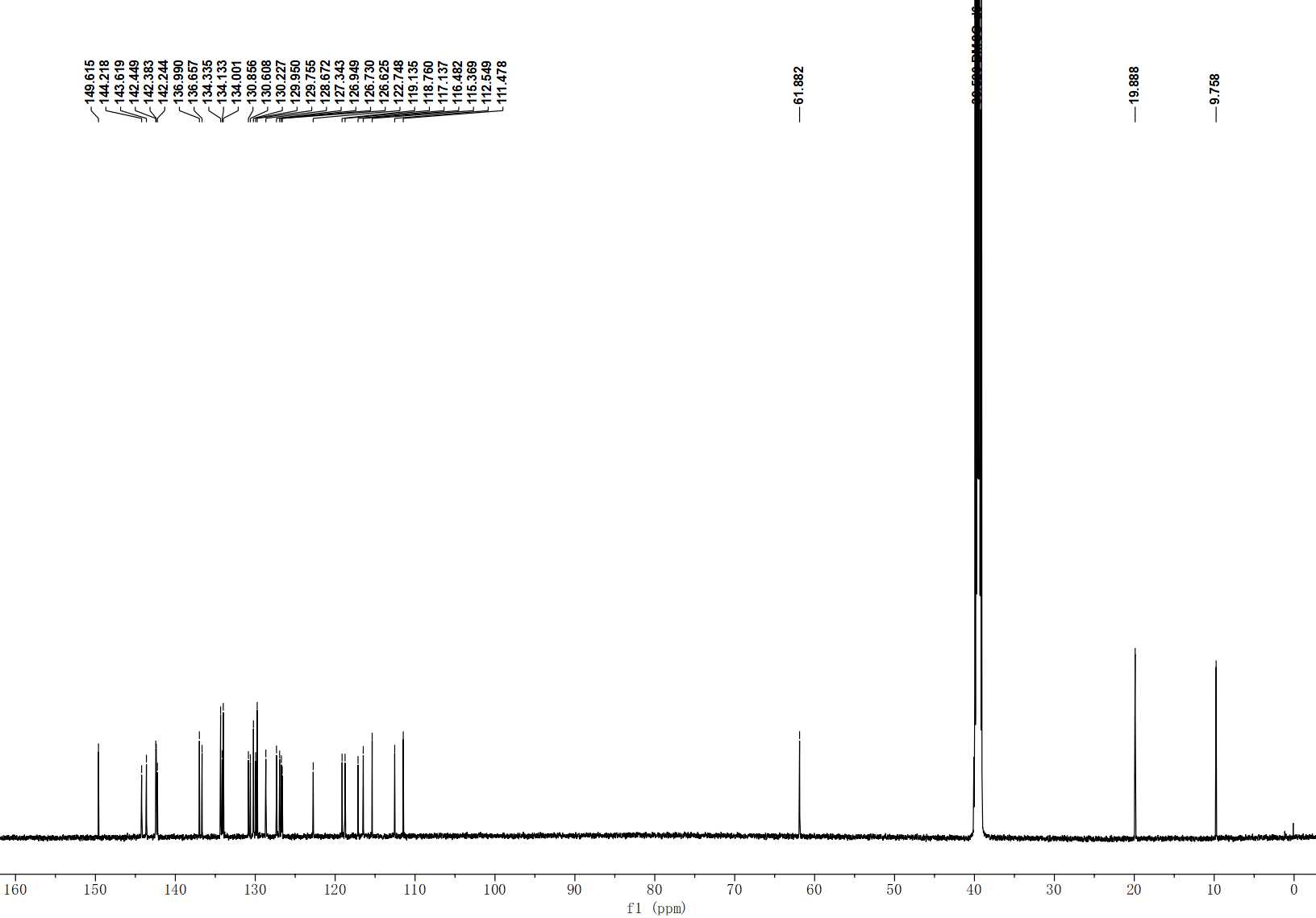


^1^H NMR, ^13^C NMR spectra of compound **a9**


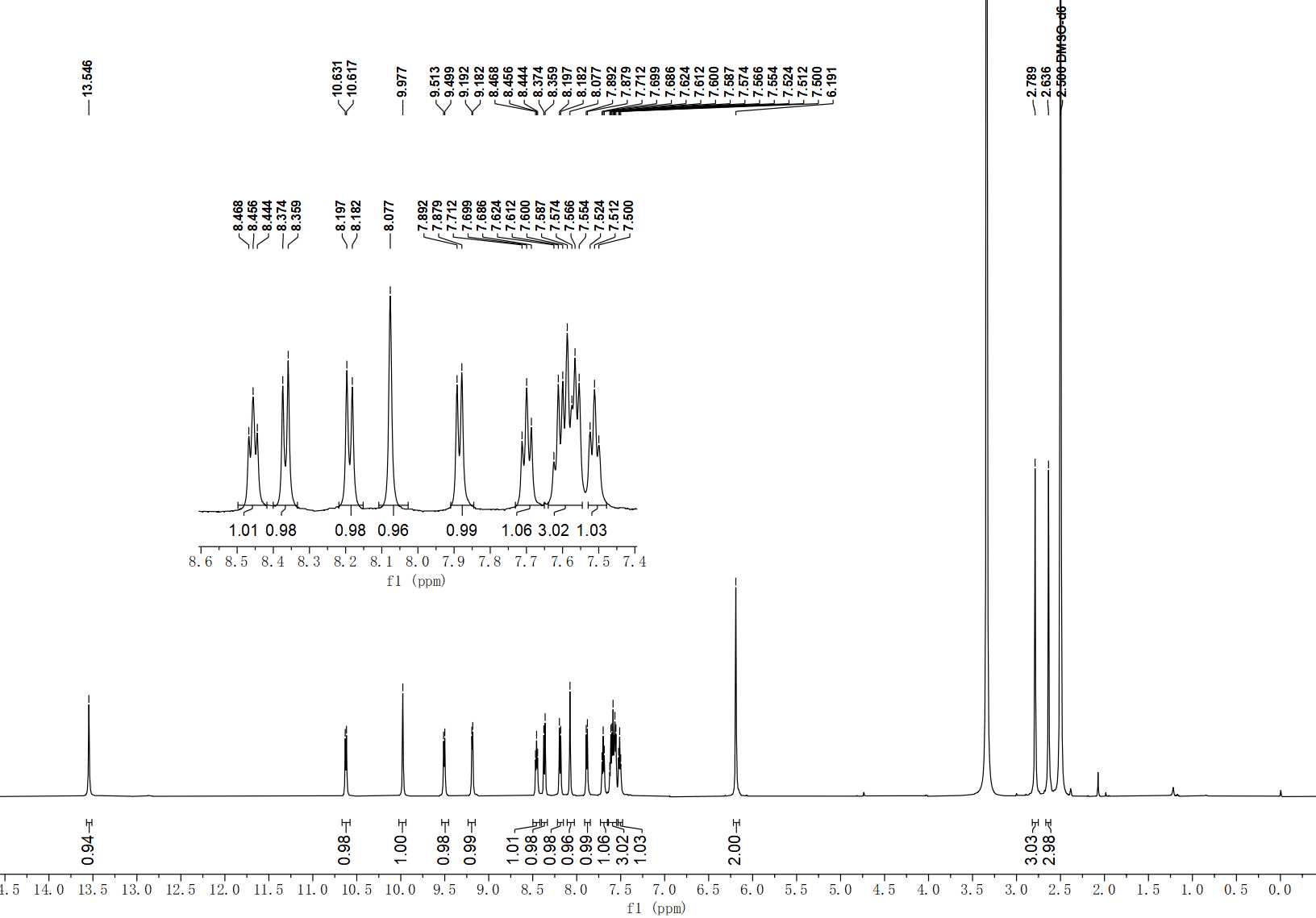


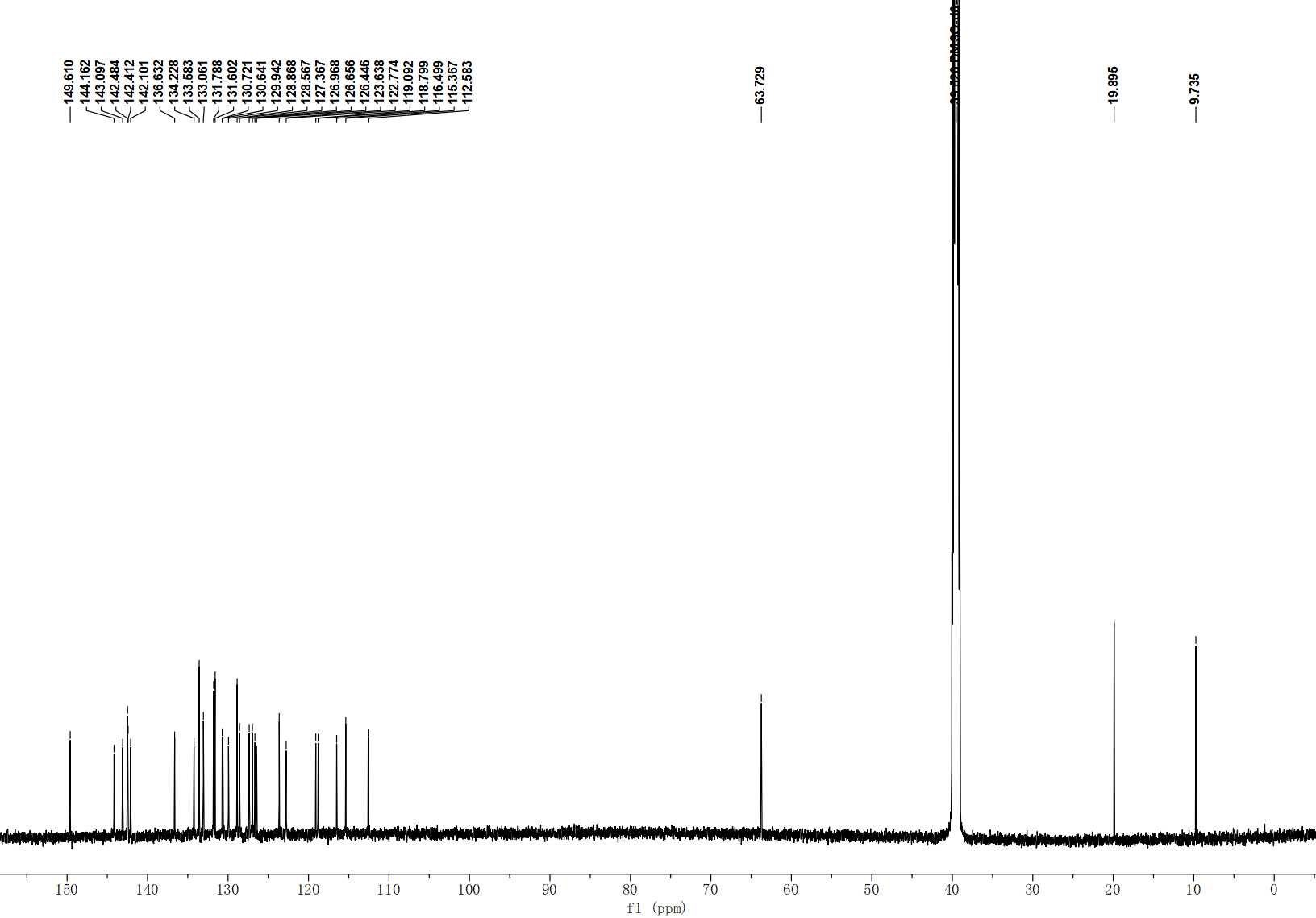


^1^H NMR, ^13^C NMR spectra of compound **a10**


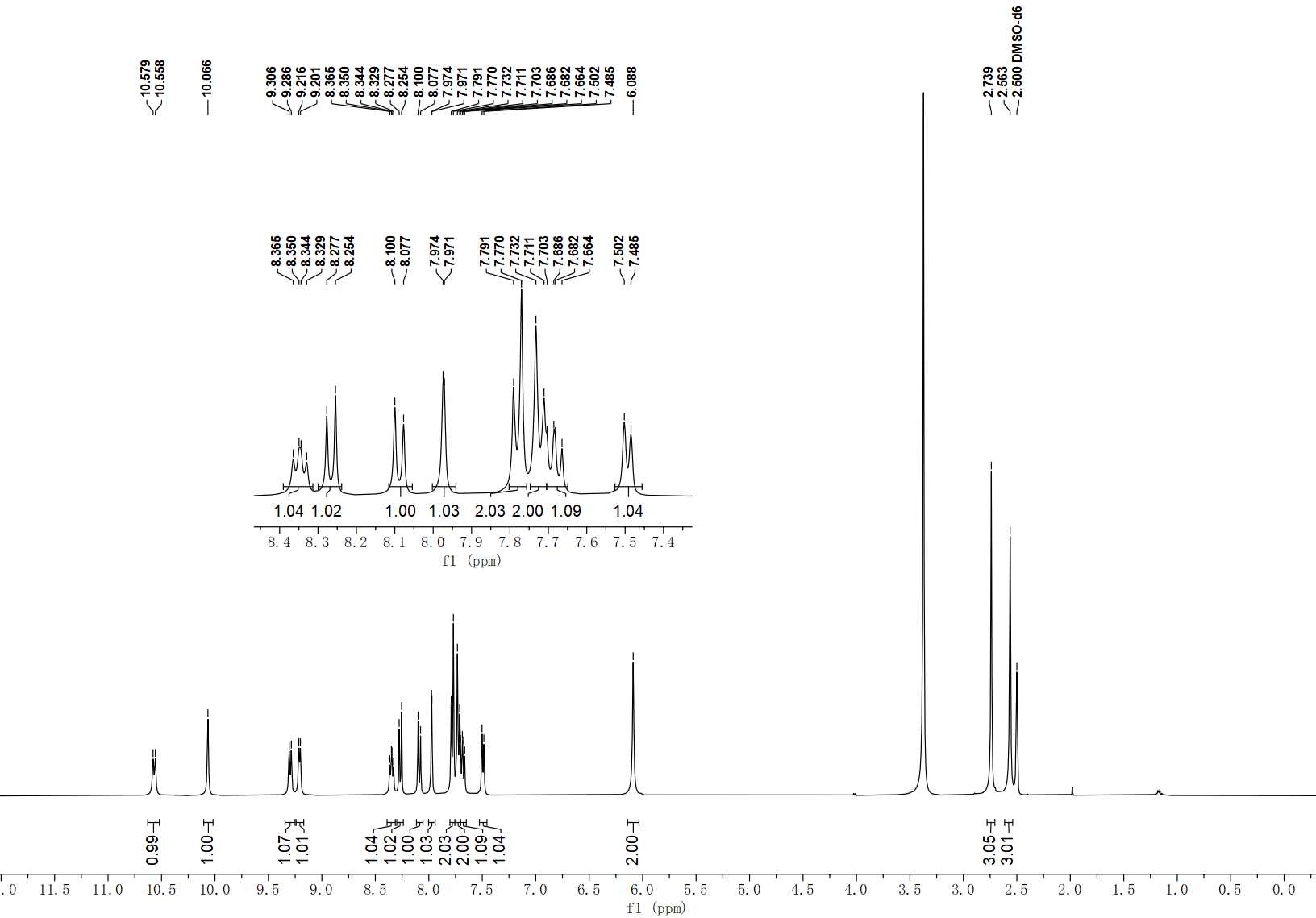


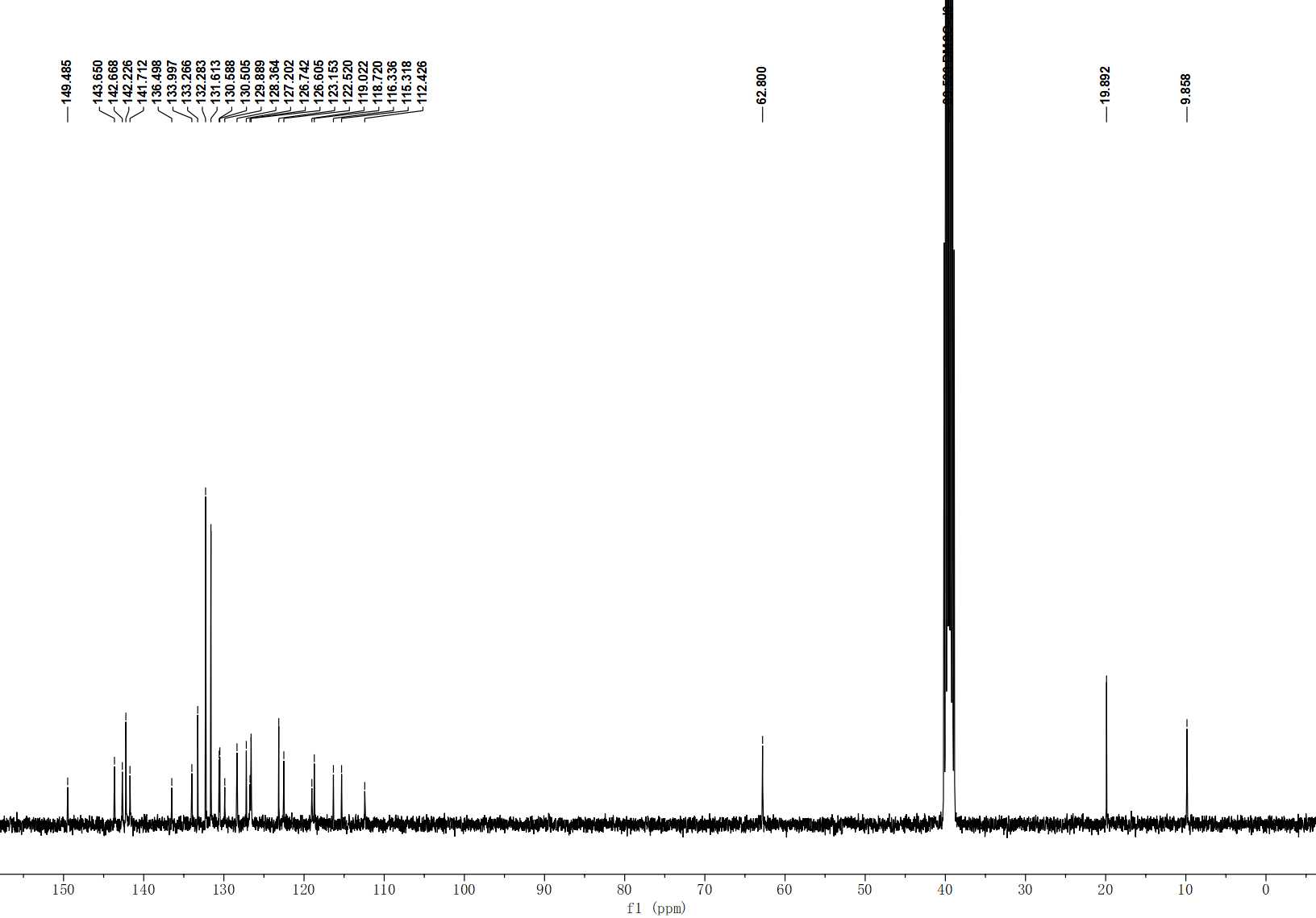


^1^H NMR, ^13^C NMR spectra of compound **a11**


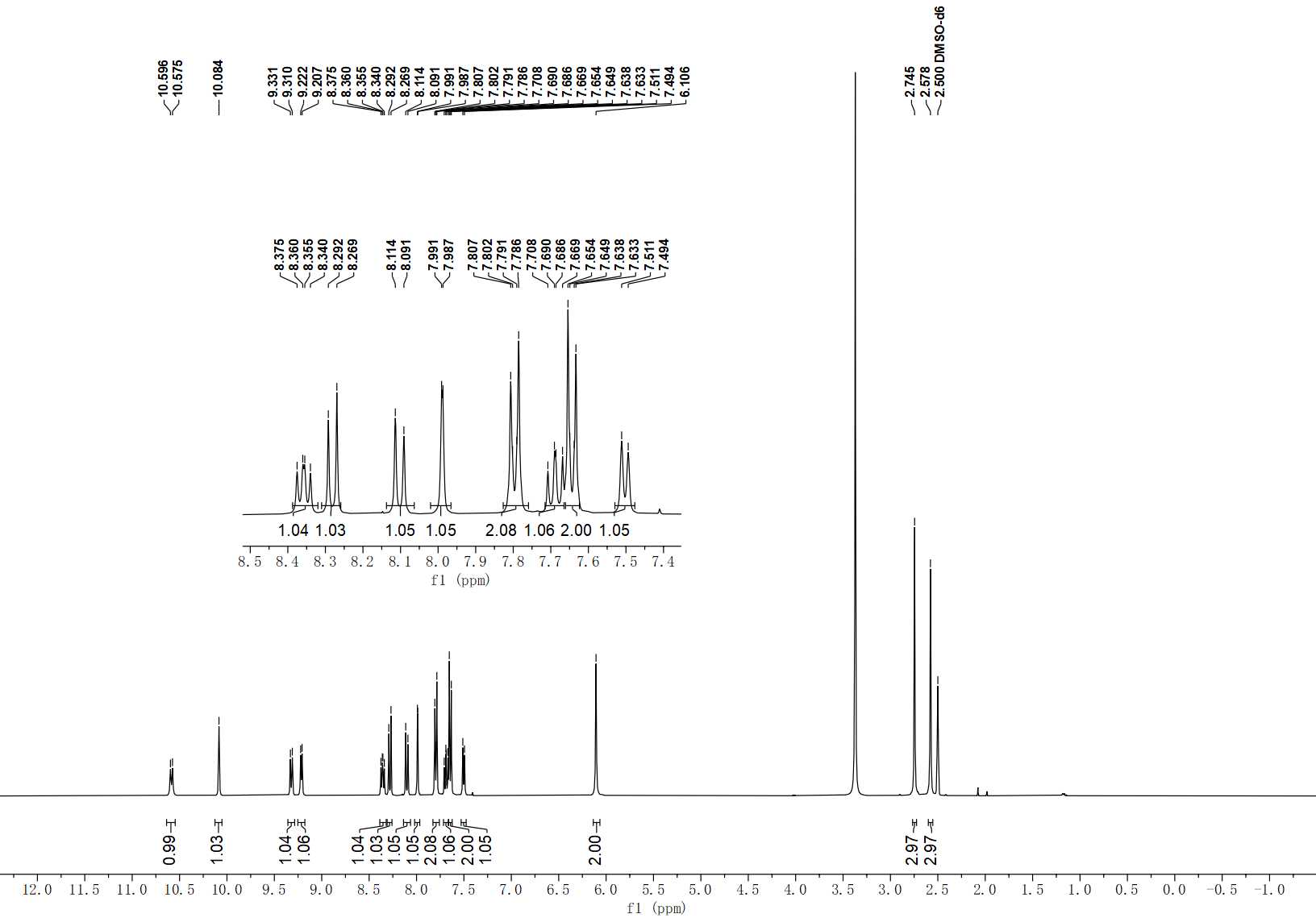


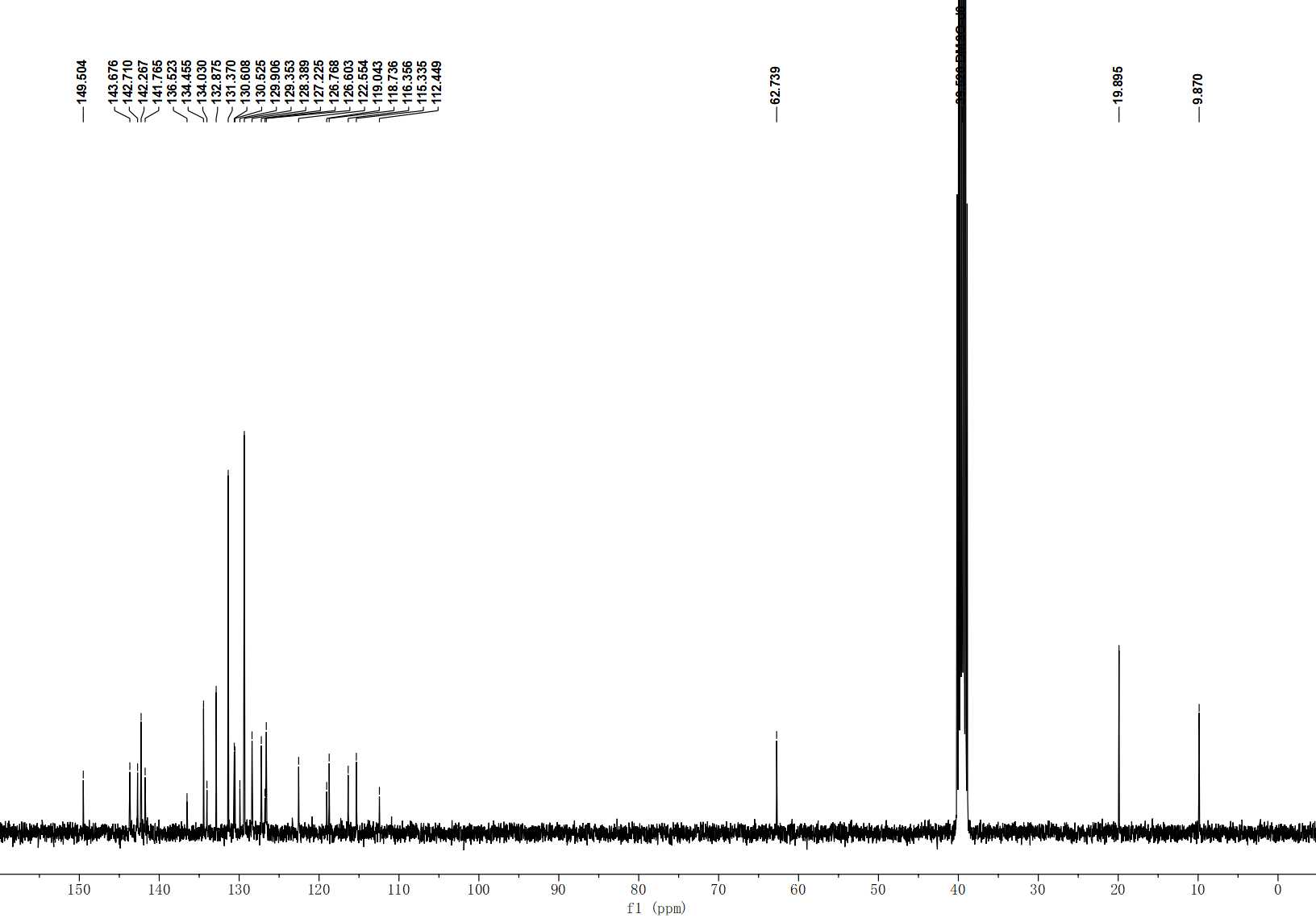


^1^H NMR, ^13^C NMR spectra of compound **a12**


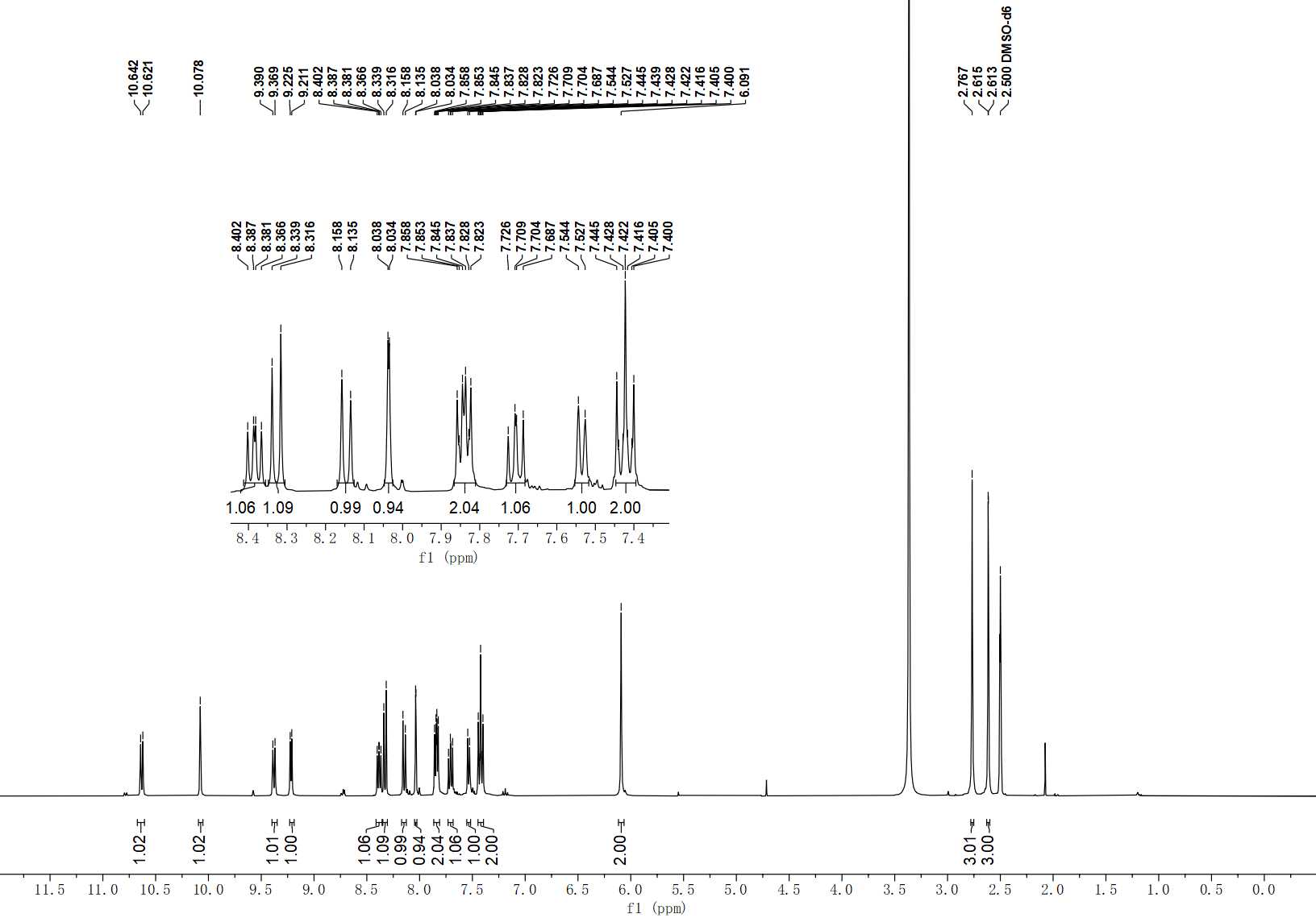


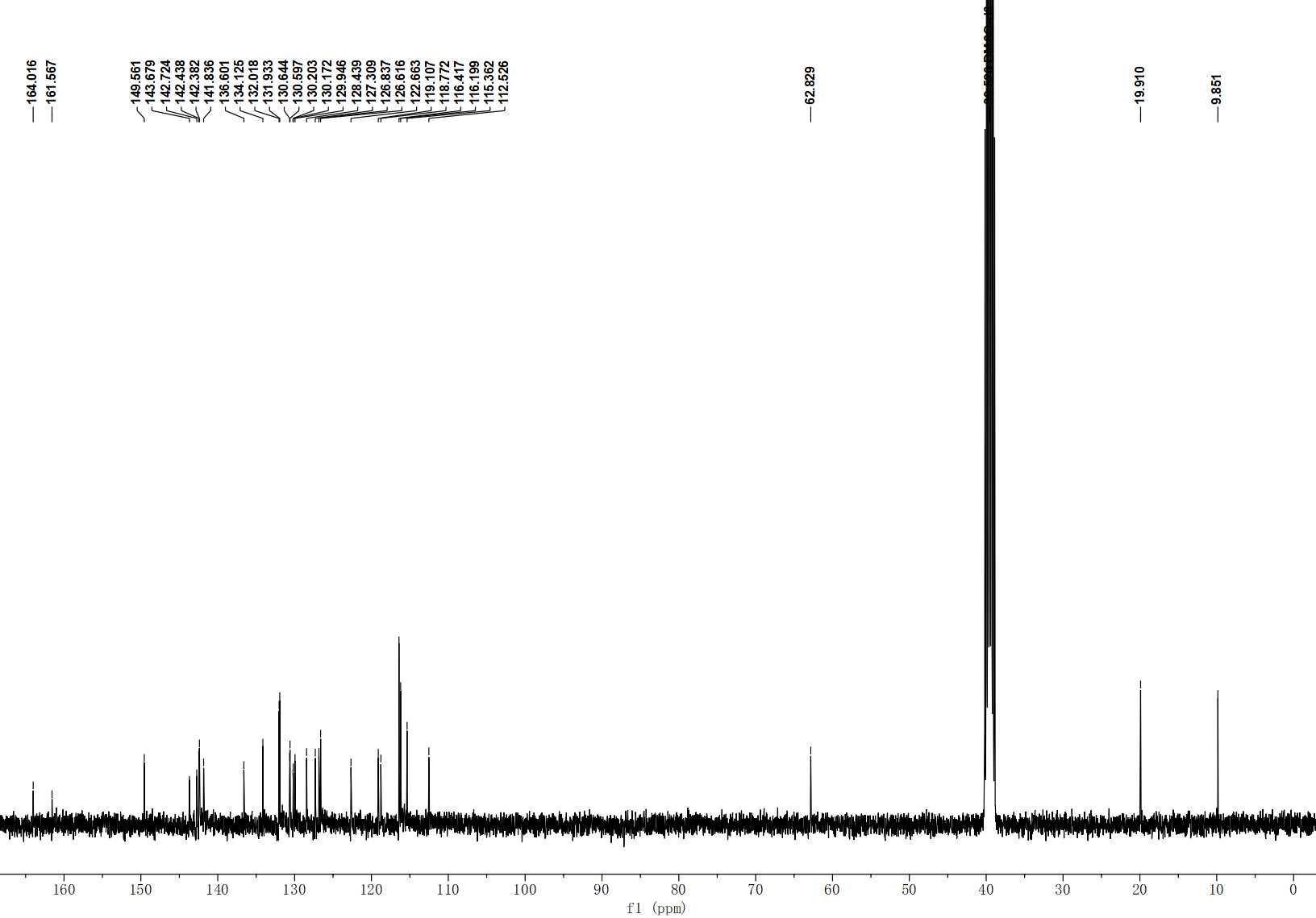


^1^H NMR, ^13^C NMR spectra of compound **a13**


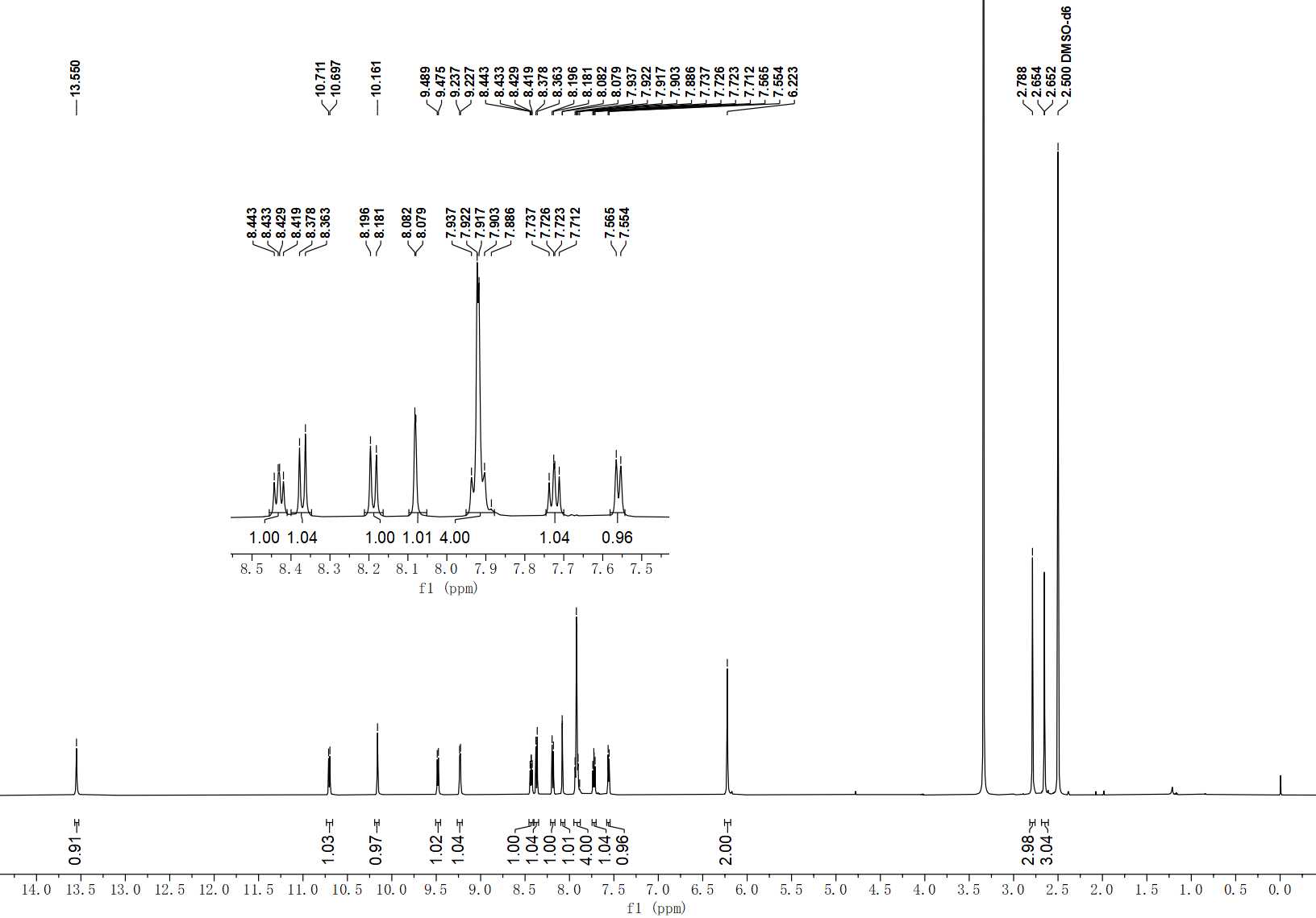


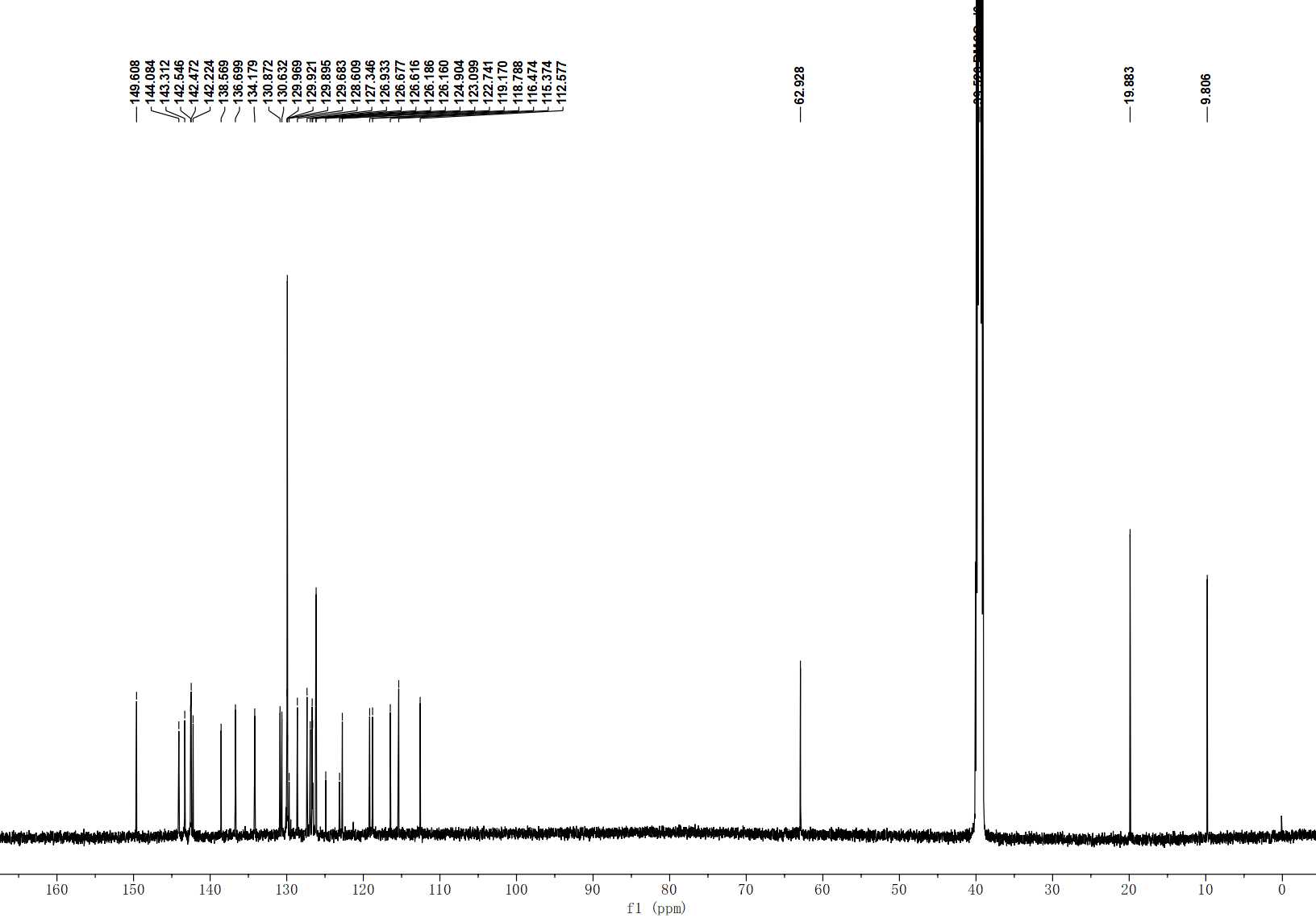


^1^H NMR, ^13^C NMR spectra of compound **a14**


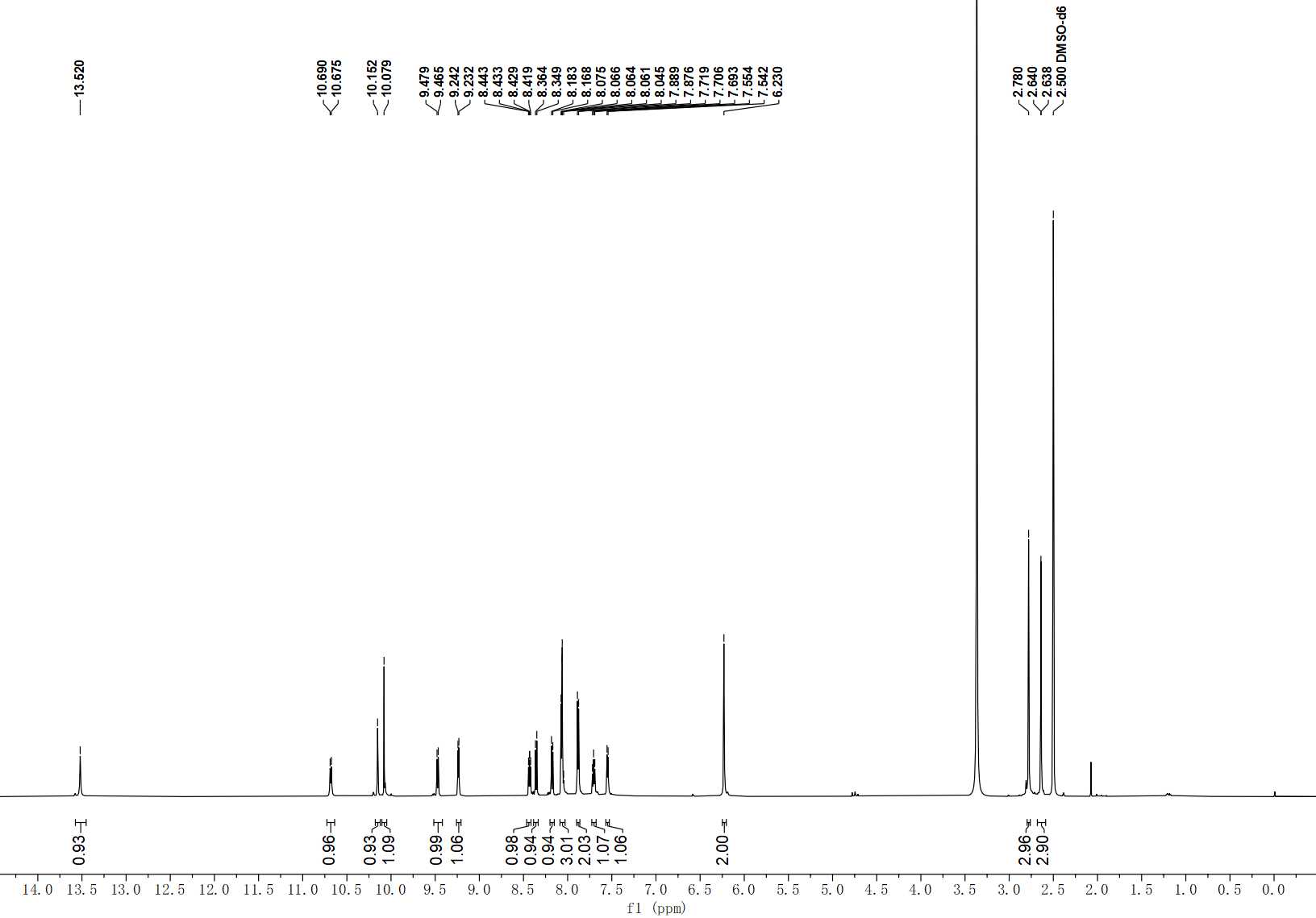


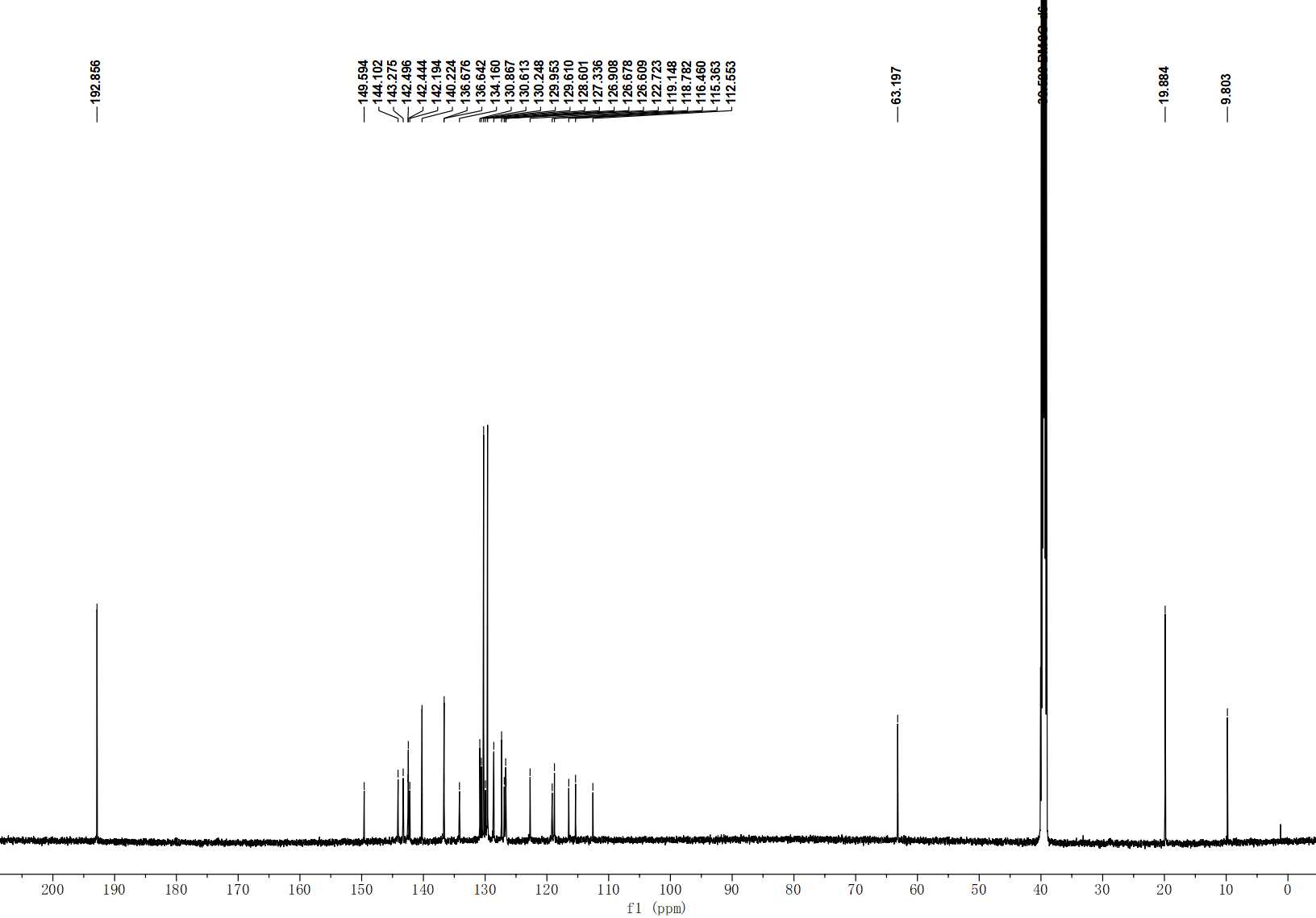


^1^H NMR, ^13^C NMR spectra of compound **a15**


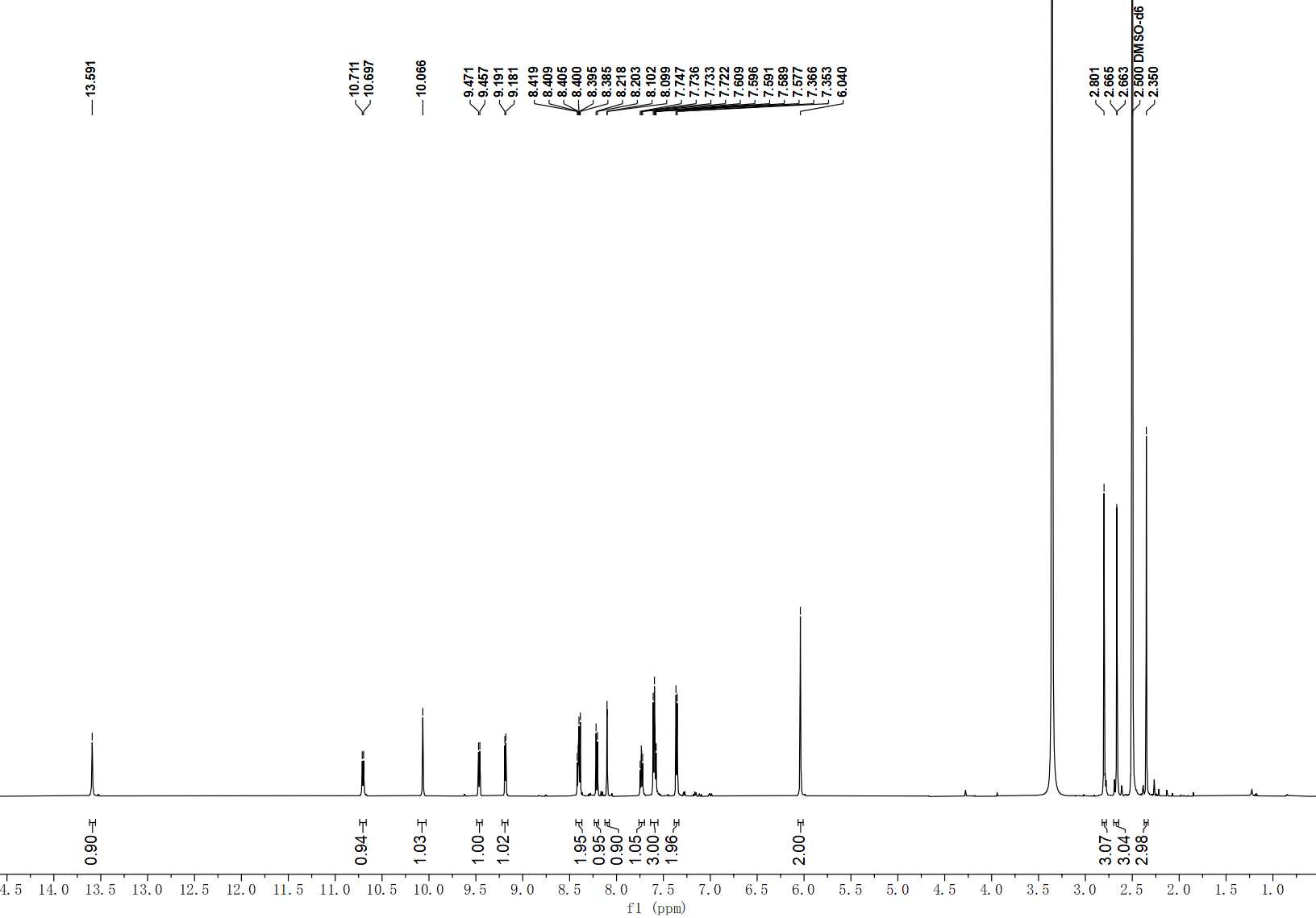


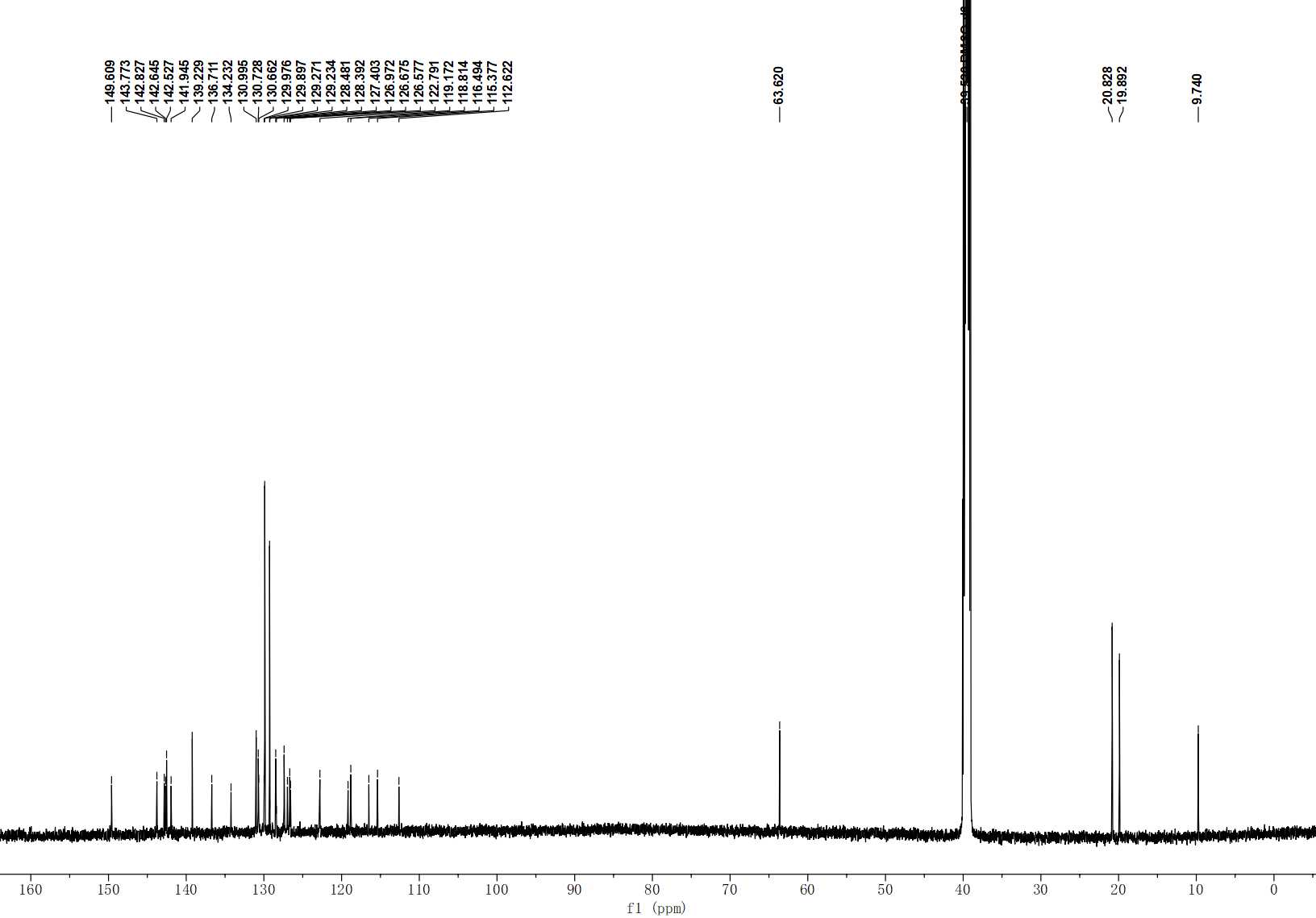


^1^H NMR, ^13^C NMR spectra of compound **a16**


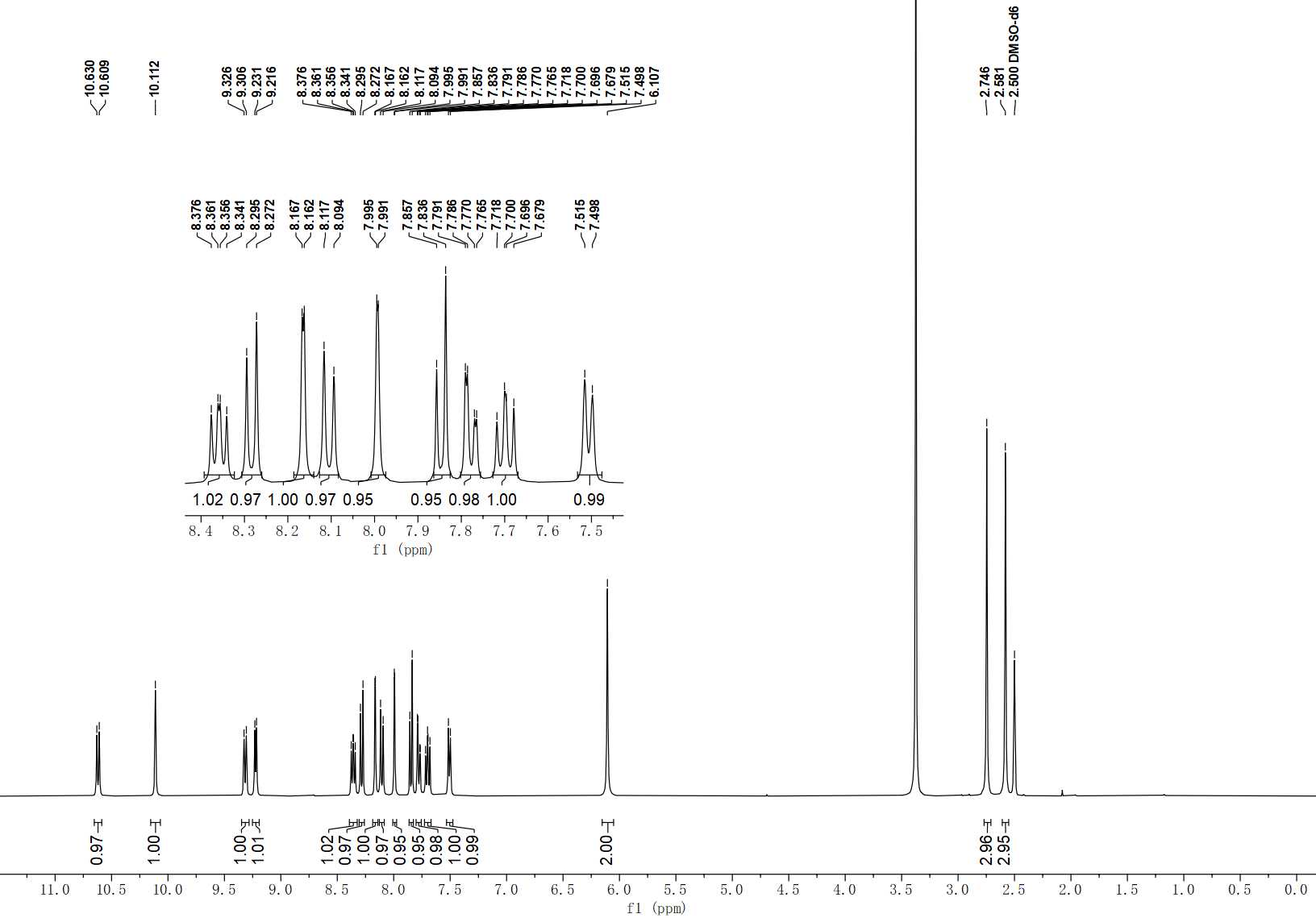


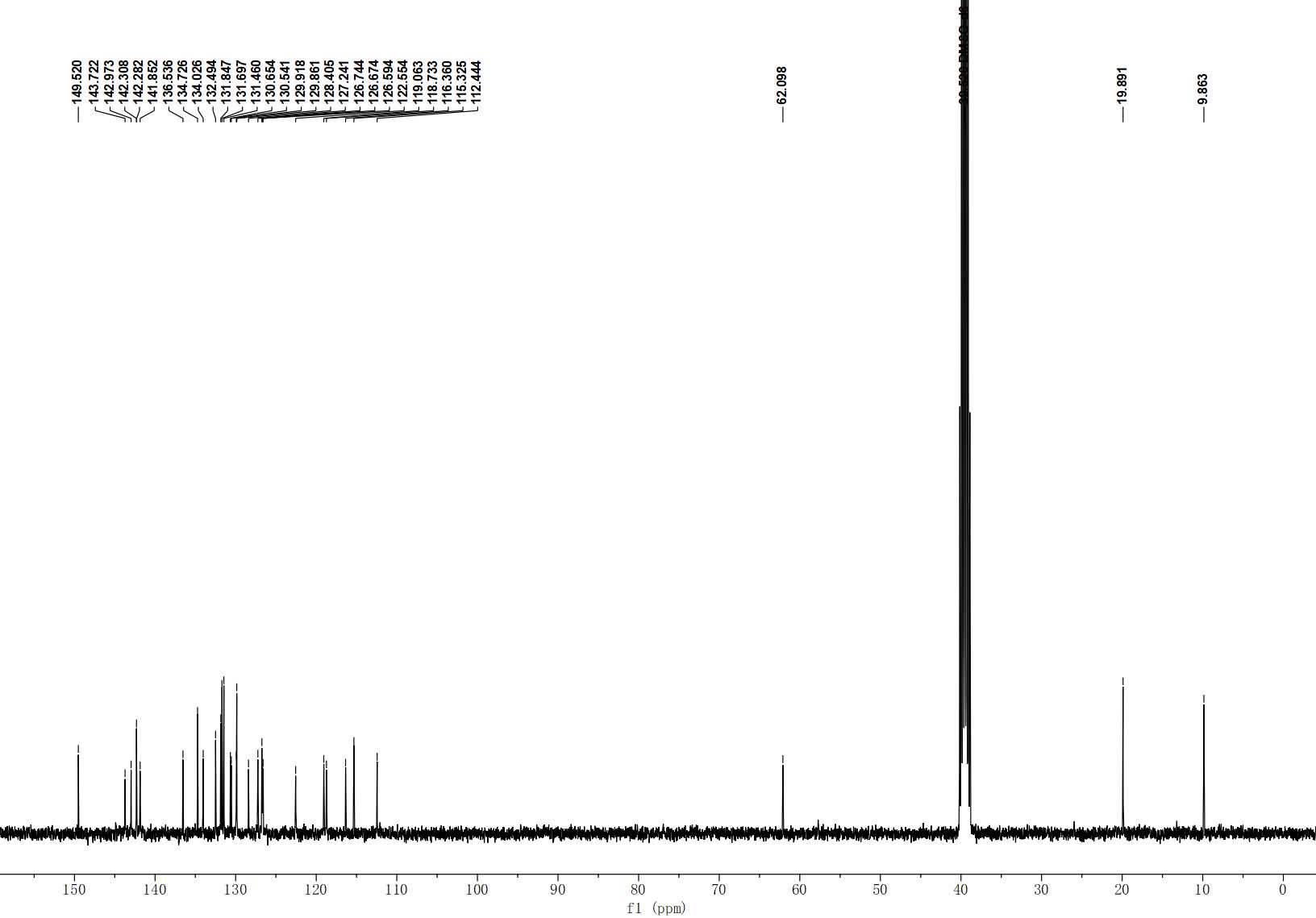


^1^H NMR, ^13^C NMR spectra of compound **a17**


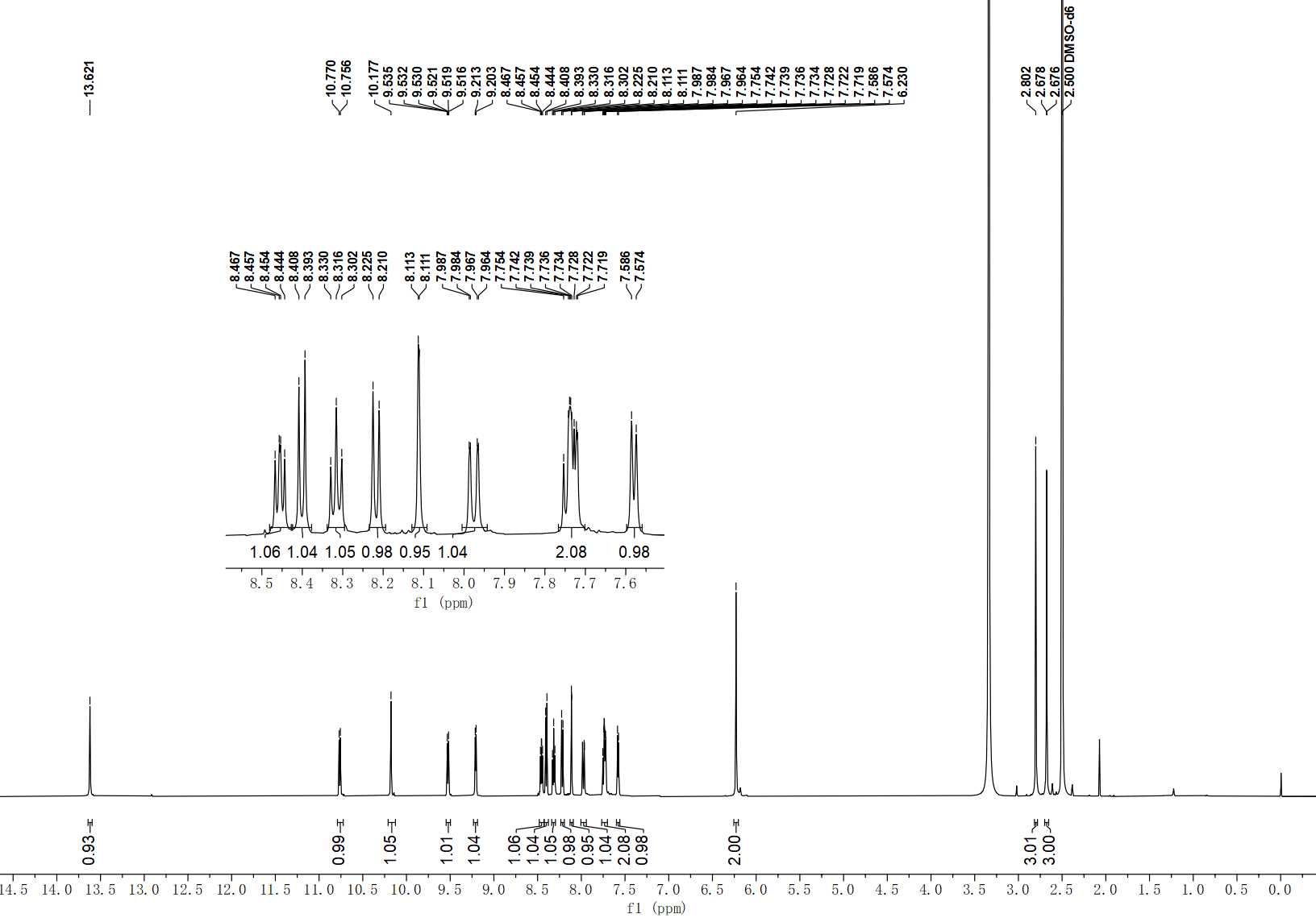


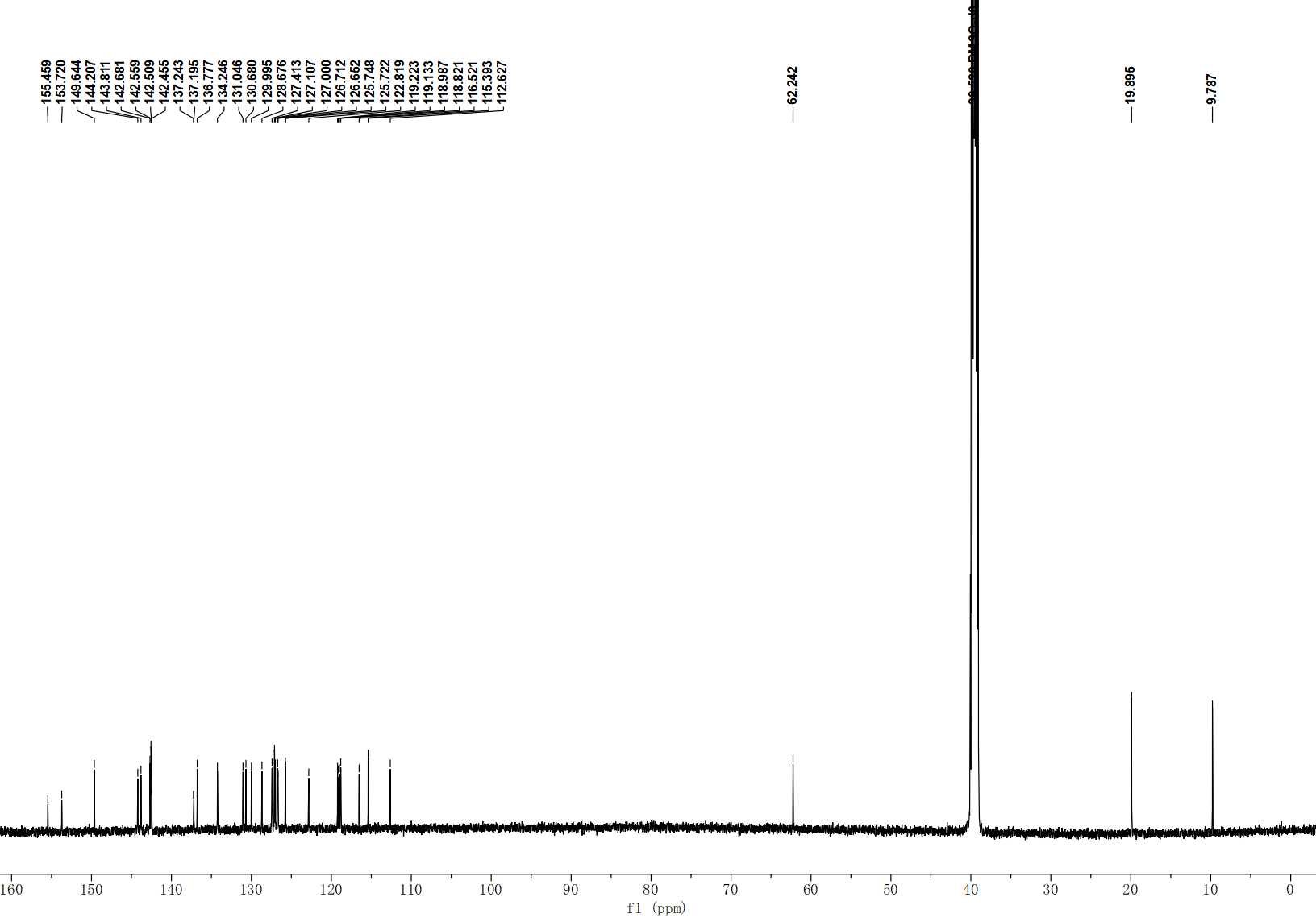


^1^H NMR, ^13^C NMR spectra of compound **a18**


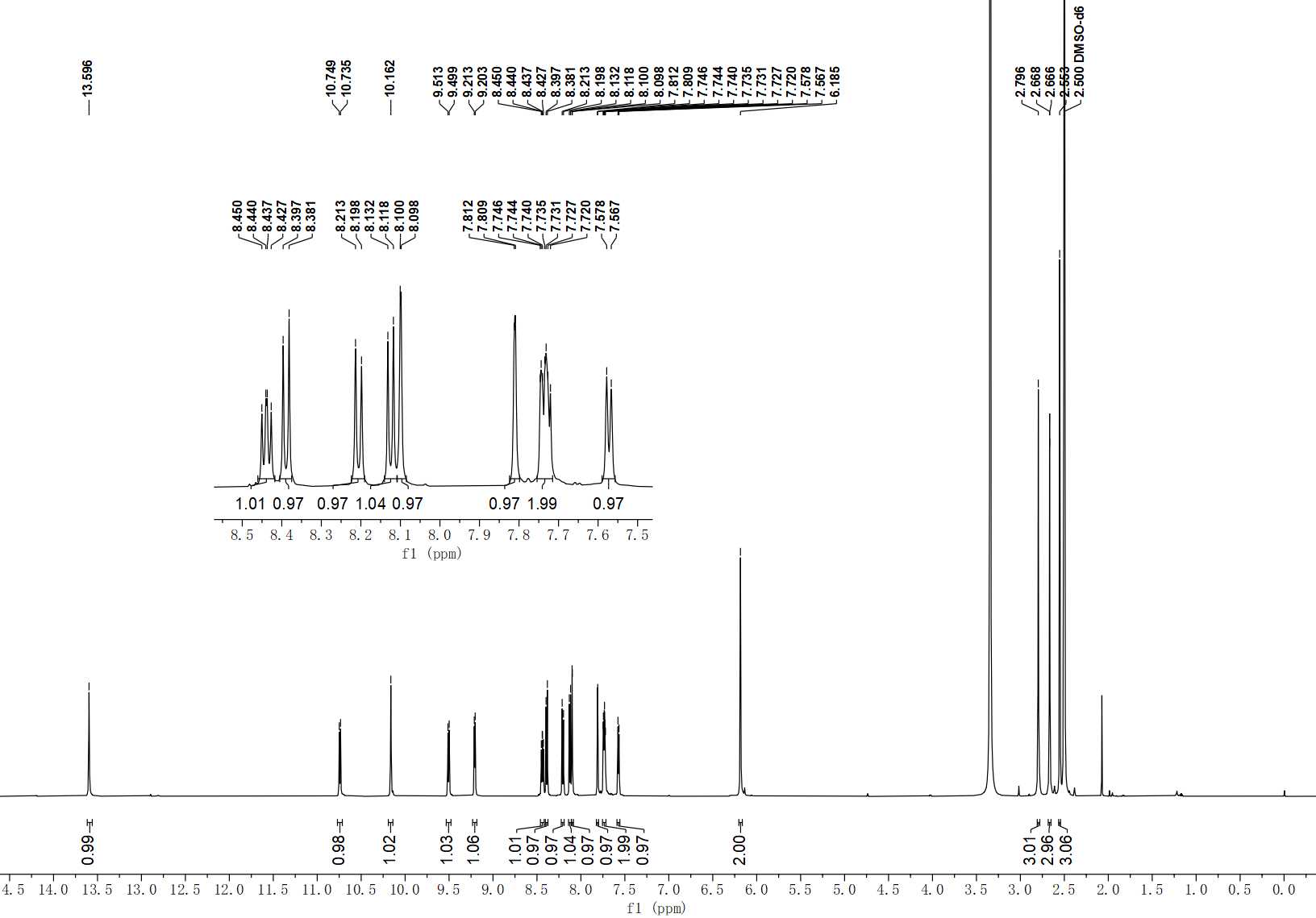


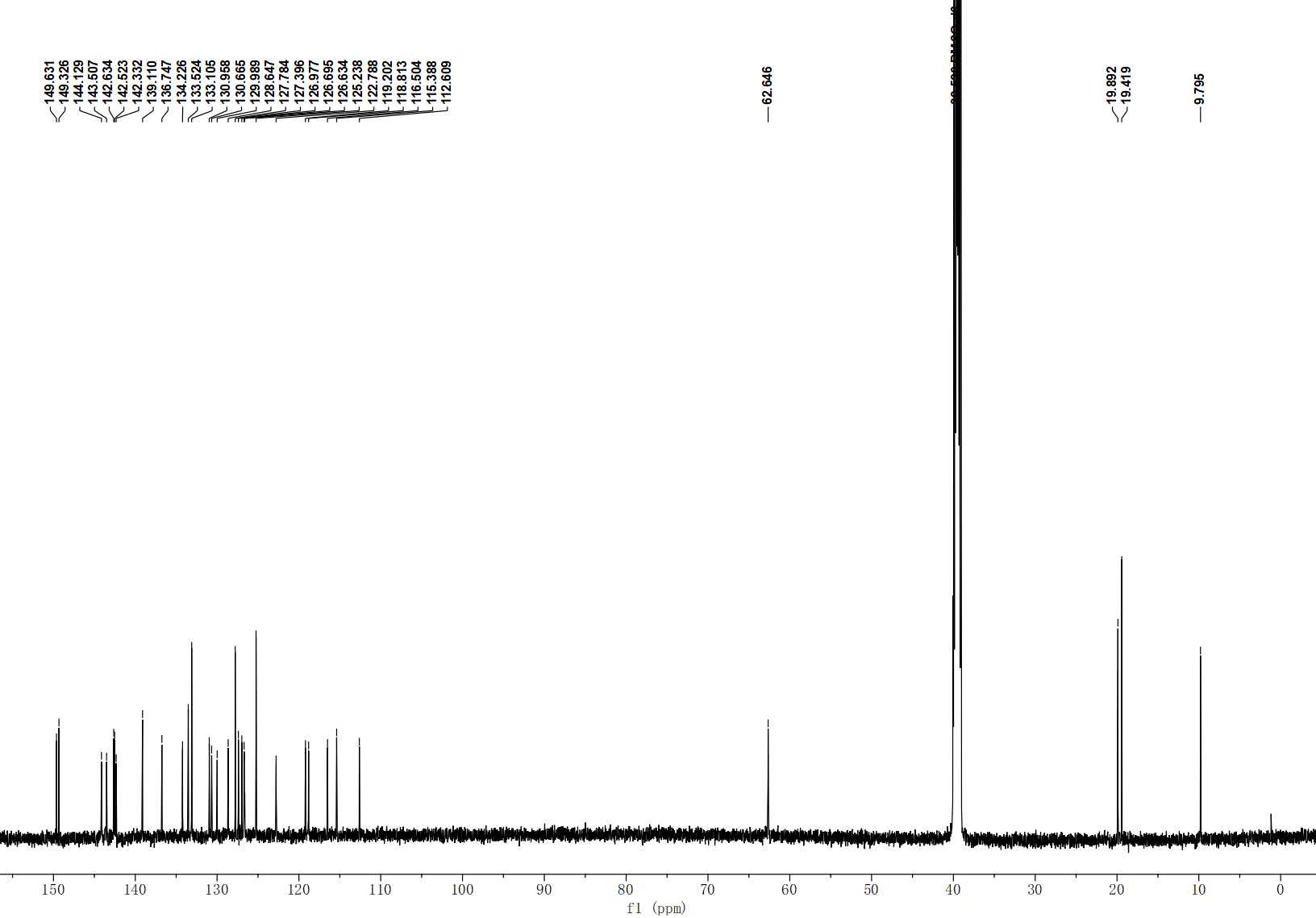


^1^H NMR, ^13^C NMR spectra of compound **a19**


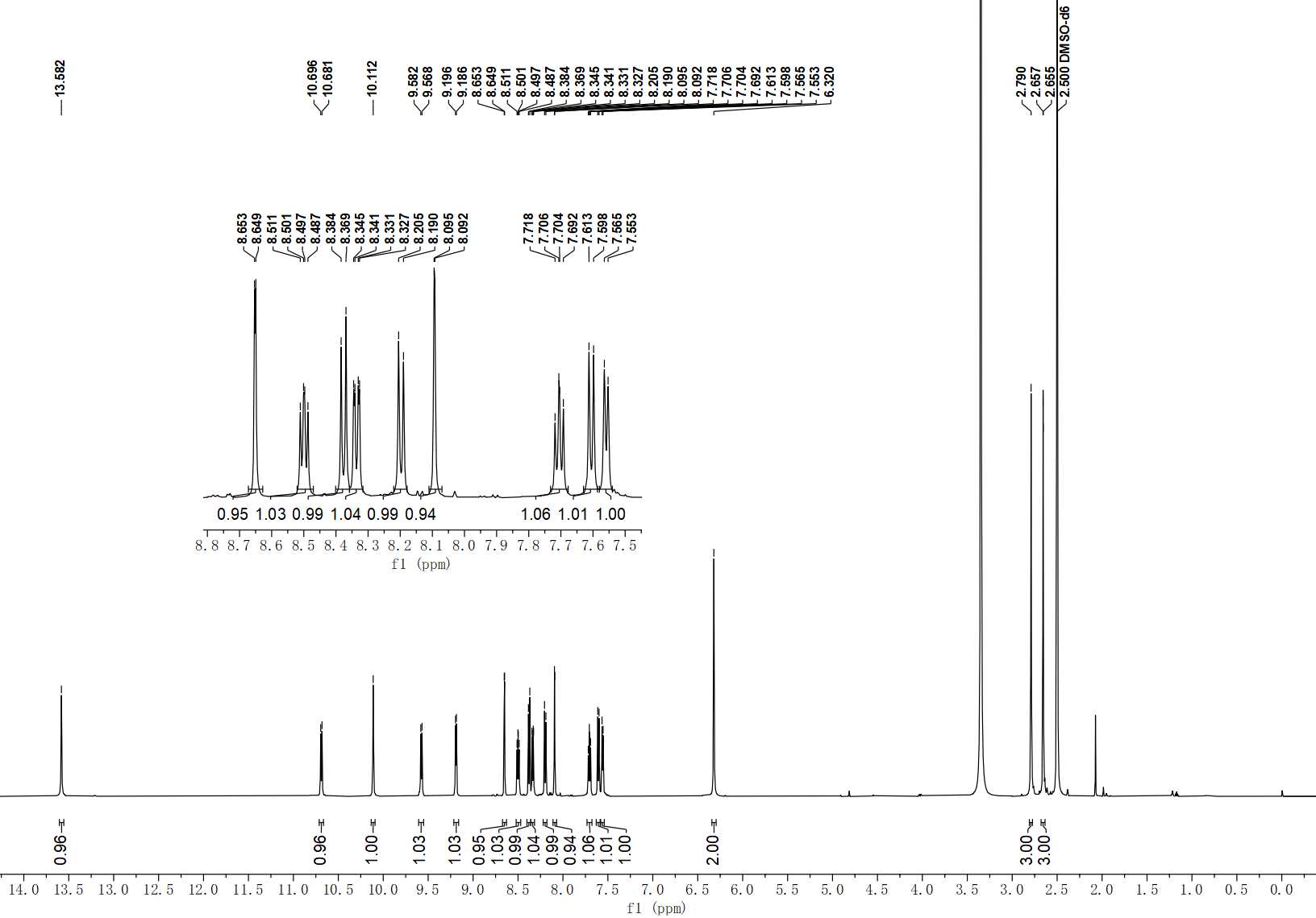


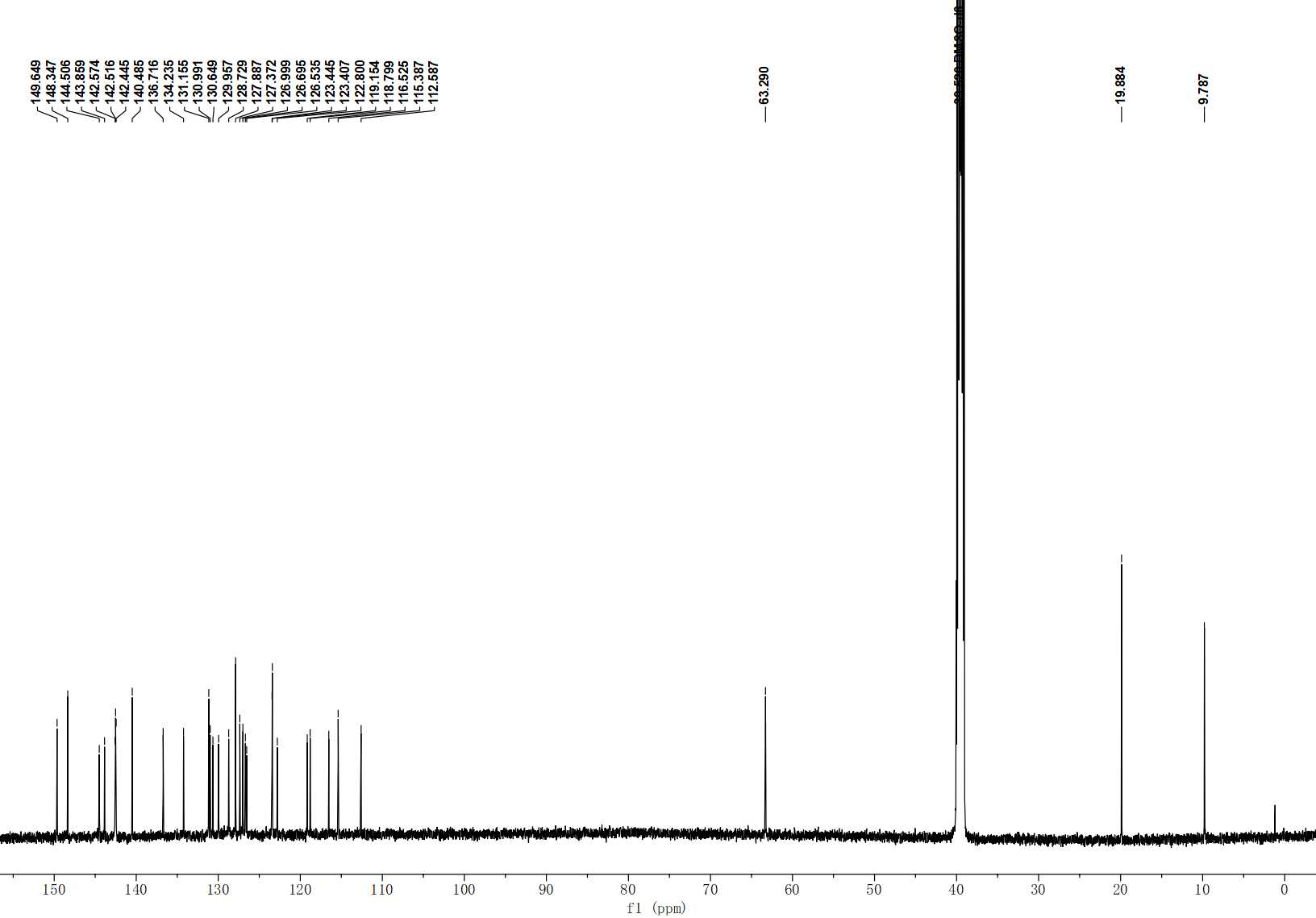


^1^H NMR, ^13^C NMR spectra of compound **a20**


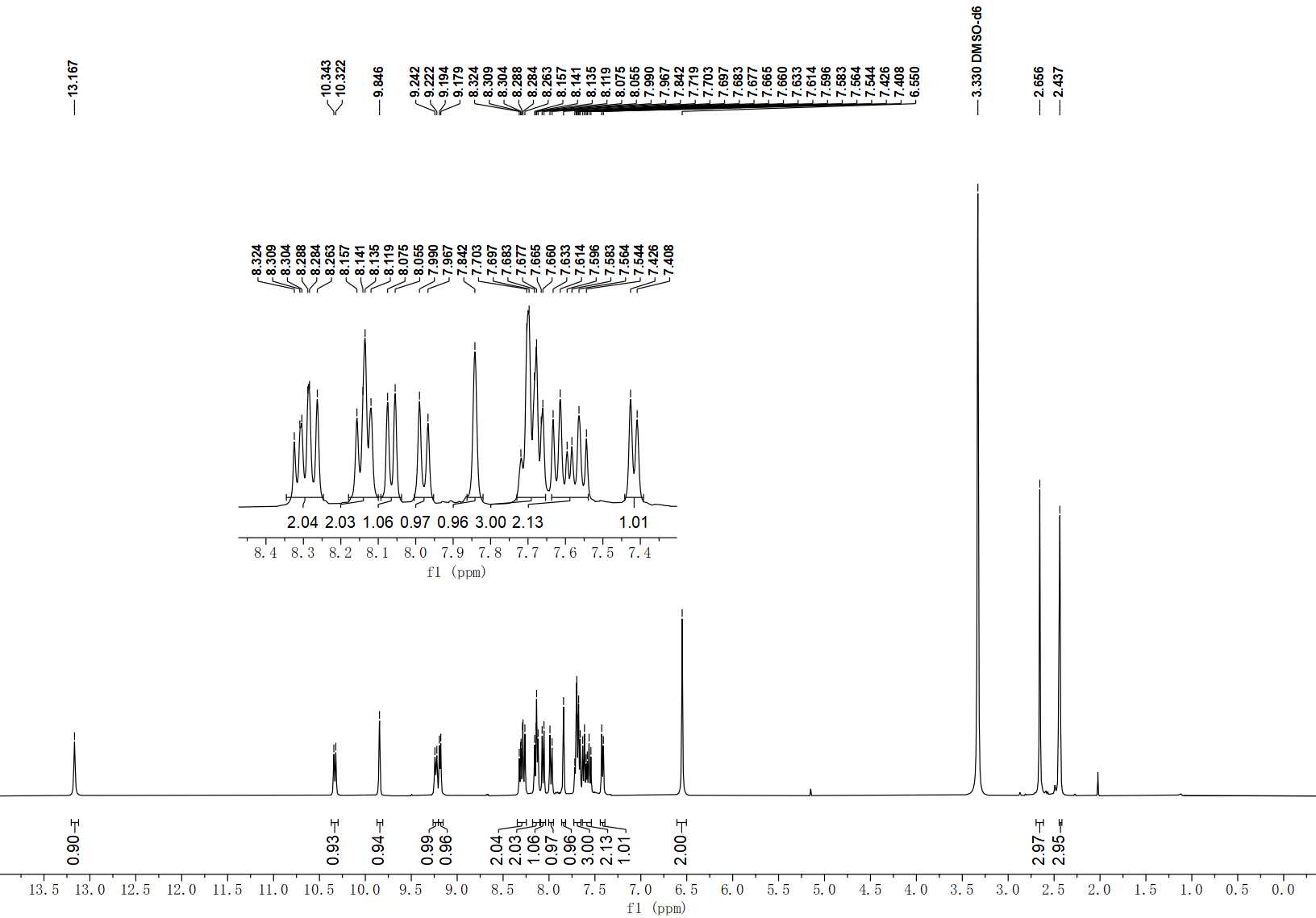


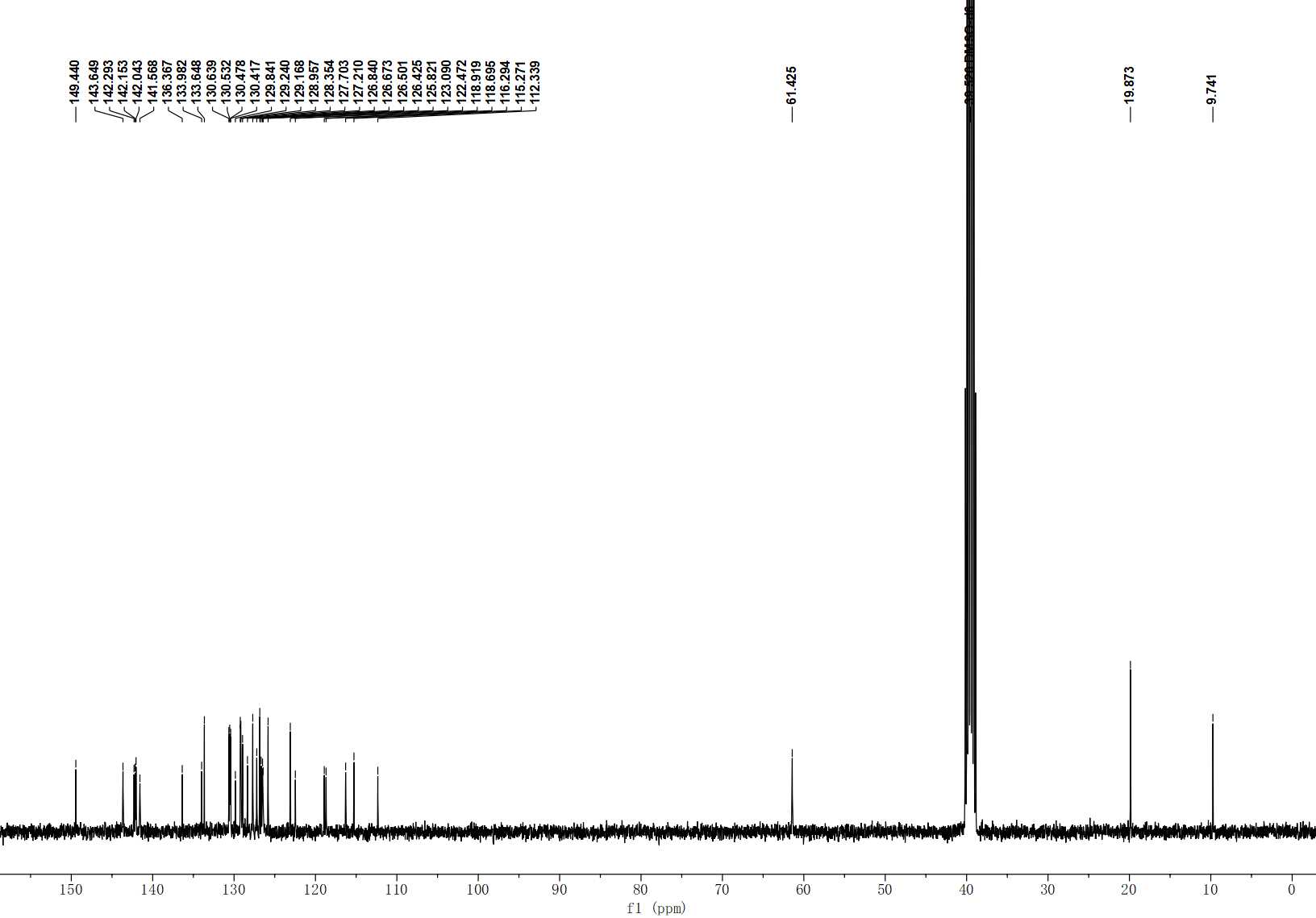


^1^H NMR, ^13^C NMR spectra of compound **a21**


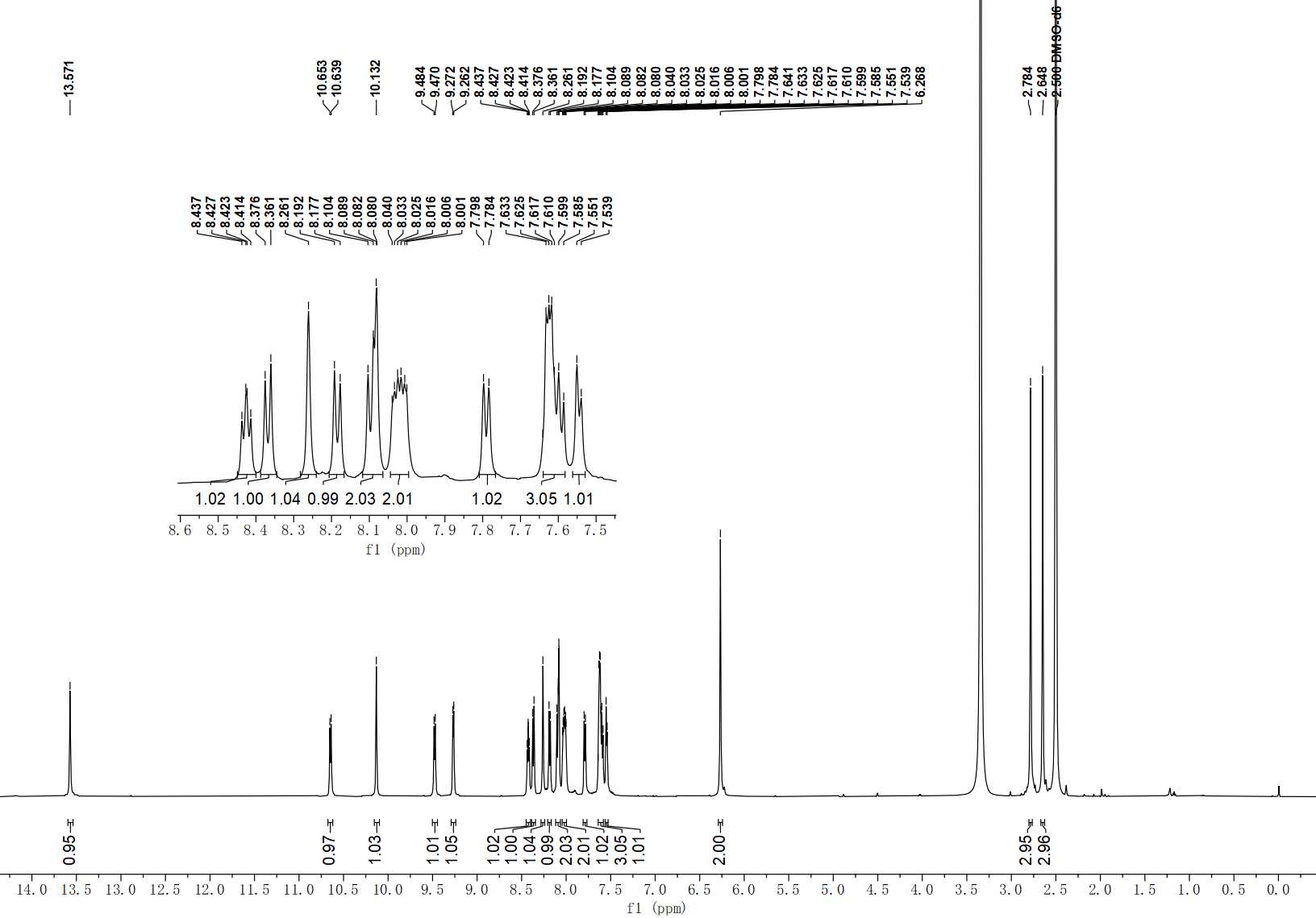


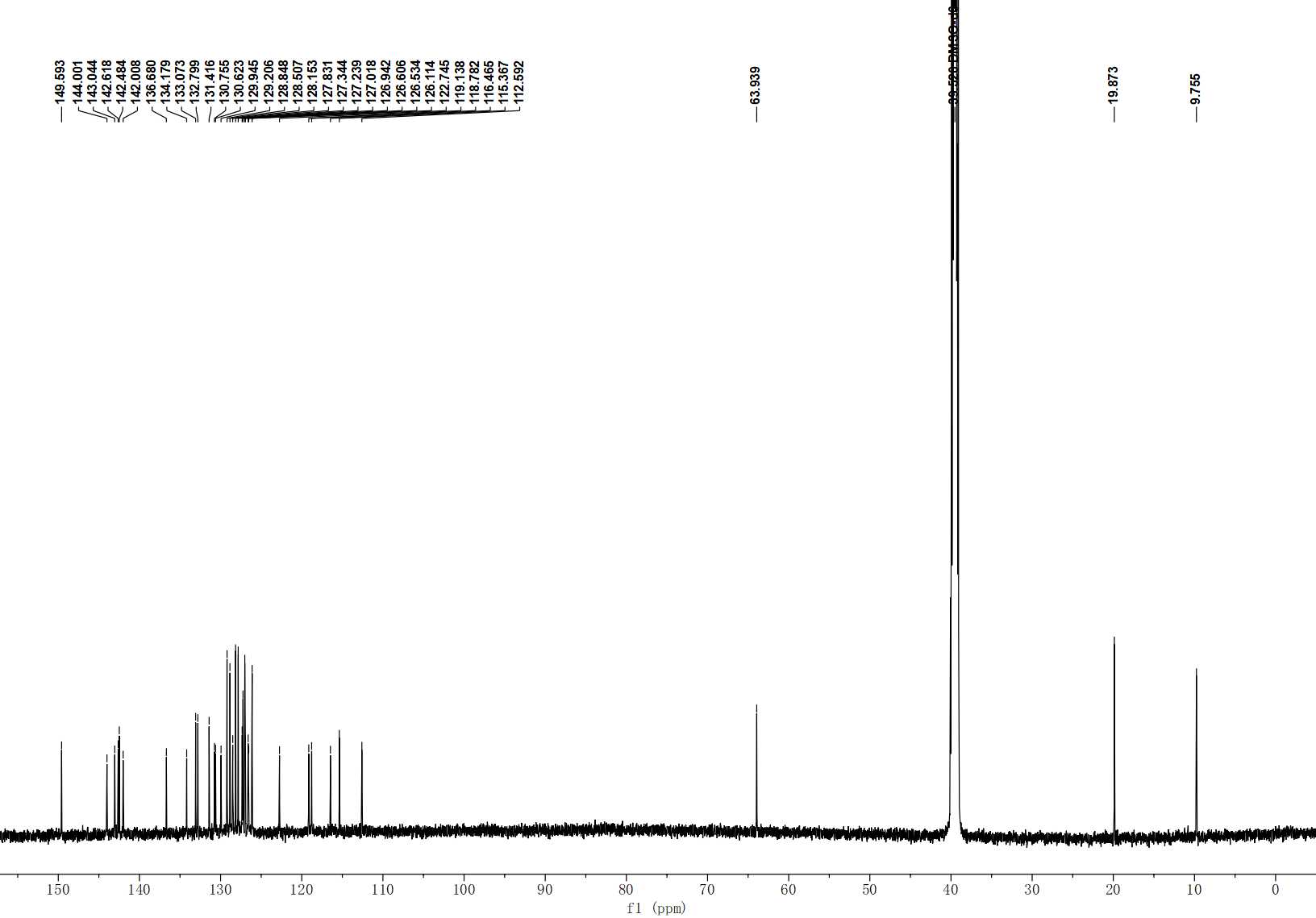


^1^H NMR, ^13^C NMR spectra of compound **a22**


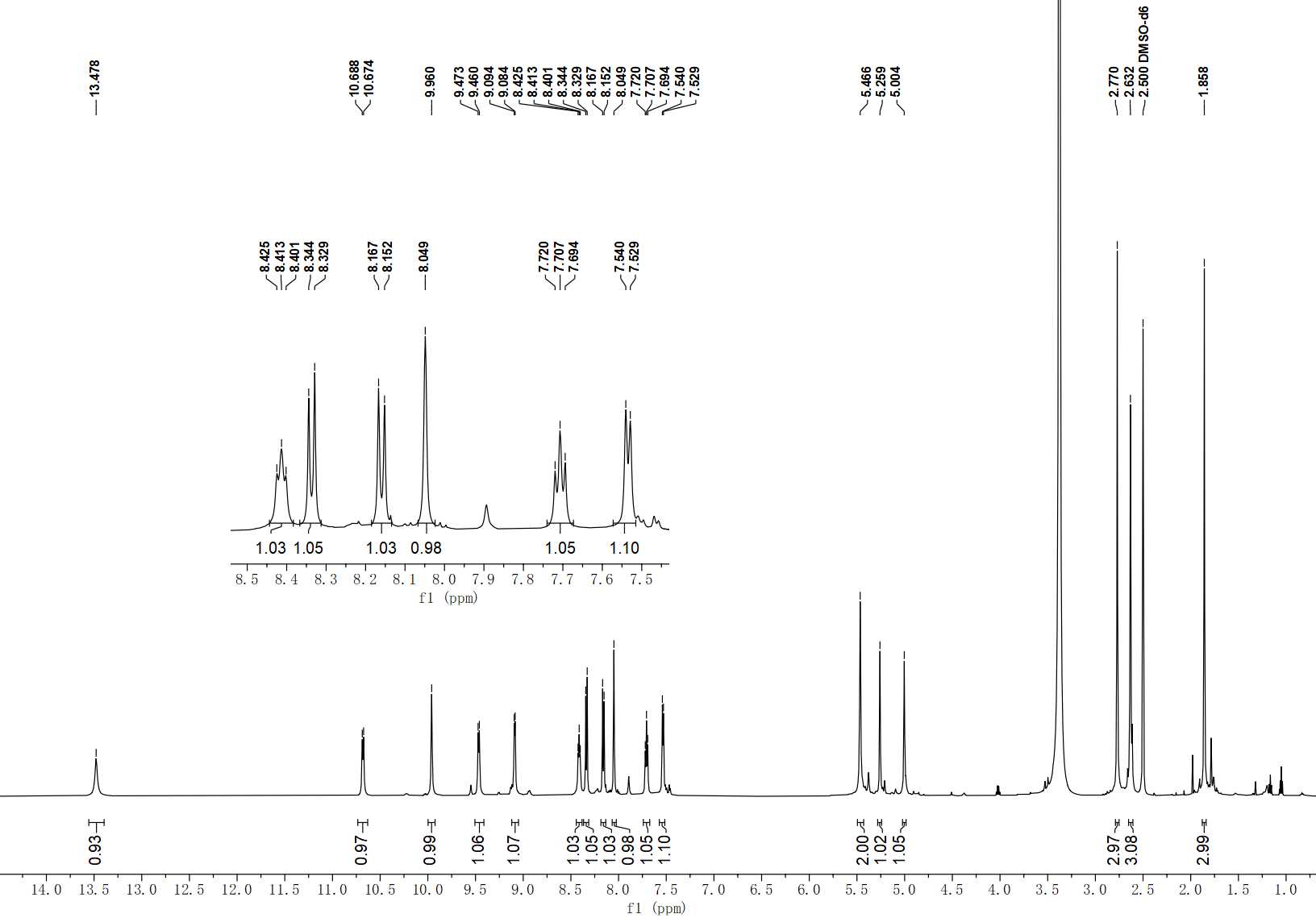


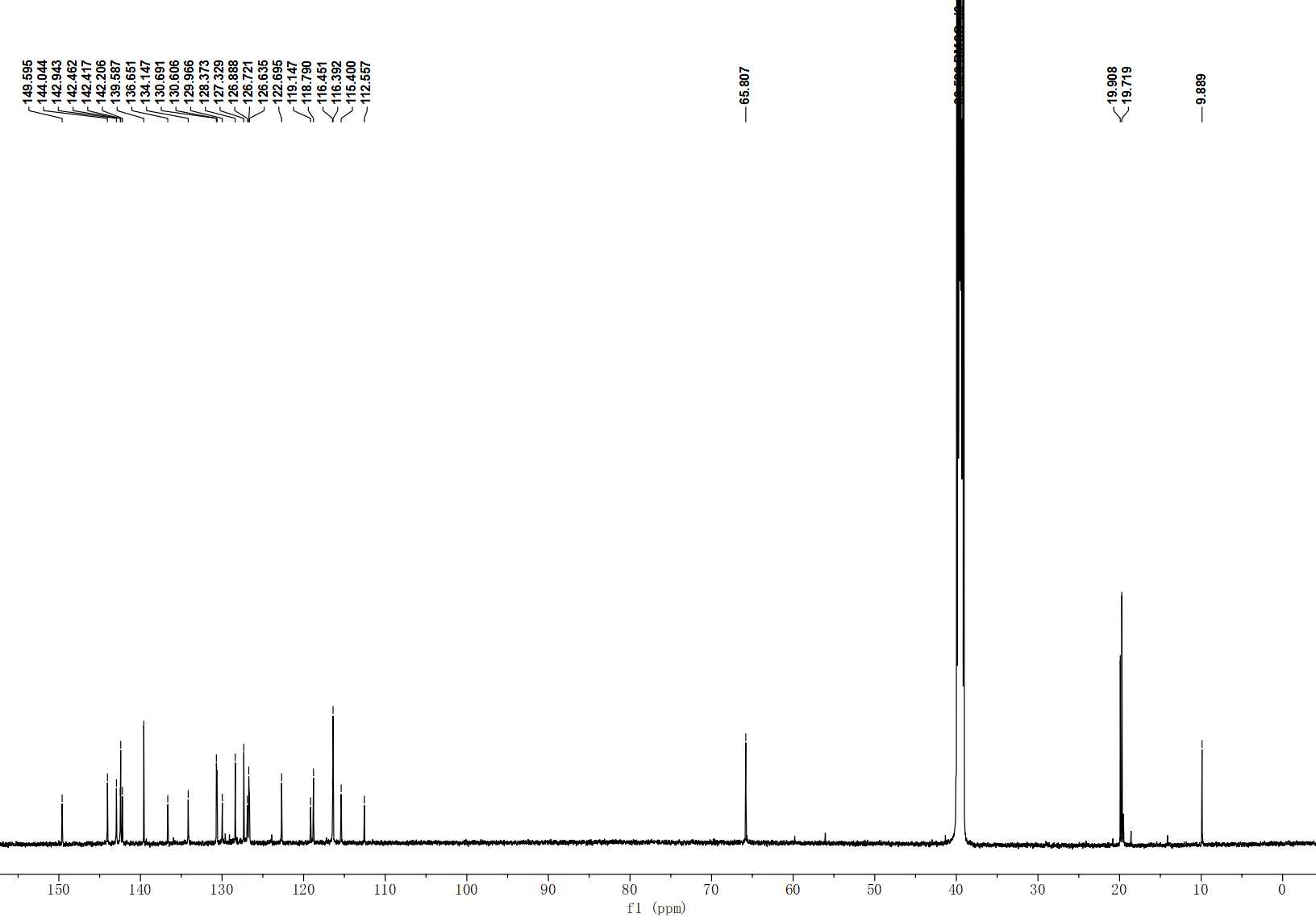

Supplement: Supplementary file 1 — Supplementary material 1. [file 13659_2025_534_MOESM1_ESM.doc]
